# Design, synthesis, molecular modeling and biological evaluation of novel Benzoxazole-Benzamide conjugates *via* a 2-Thioacetamido linker as potential anti-proliferative agents, VEGFR-2 inhibitors and apoptotic inducers

**DOI:** 10.1080/14756366.2022.2081844

**Published:** 2022-05-30

**Authors:** Ibrahim H. Eissa, Radwan El-Haggar, Mohammed A. Dahab, Marwa F. Ahmed, Hazem A. Mahdy, Reem I. Alsantali, Alaa Elwan, Nicolas Masurier, Samar S. Fatahala

**Affiliations:** a Pharmaceutical Medicinal Chemistry & Drug Design Department, Faculty of Pharmacy (Boys), Al-Azhar University, Cairo, Egypt; b Pharmaceutical Chemistry Department, Faculty of Pharmacy, Helwan University, Cairo, Egypt; c Institut des Biomolécules Max Mousseron (IBMM), UMR 5247, CNRS, Université de Montpellier, ENSCM, Montpellier, France; d Department of Pharmaceutical Chemistry, College of Pharmacy, Taif University, Taif, Saudi Arabia; e Pharmaceutical Organic Chemistry Department, Faculty of Pharmacy, Helwan University, Cairo, Egypt

**Keywords:** Benzoxazole-benzamide, anti-cancer, apoptosis, VEGFR-2 inhibitors, Bcl-2

## Abstract

A novel series of 2-thioacetamide linked benzoxazole-benzamide conjugates **1**–**15** was designed as potential inhibitors of the vascular endothelial growth factor receptor-2 (VEGFR-2). The prepared compounds were evaluated for their potential antitumor activity and their corresponding selective cytotoxicity was estimated using normal human fibroblast (WI-38) cells. Compounds **1**, **9**–**12** and **15** showed good selectivity and displayed excellent cytotoxic activity against both HCT-116 and MCF-7 cancer cell lines compared to sorafenib, used as a reference compound. Furthermore, compounds **1** and **11** showed potent VEGFR-2 inhibitory activity. The cell cycle progression assay showed that **1** and **11** induced cell cycle arrest at G2/M phase, with a concomitant increase in the pre-G1 cell population. Further pharmacological studies showed that **1** and **11** induced apoptosis and inhibited the expression of the anti-apoptotic Bcl-2 and Bcl-xL proteins in both cell lines. Therefore, compounds **1** and **11** might serve as promising candidates for future anticancer therapy development.

## 1. Introduction

Cancer is a lethal collection of diseases characterised by uncontrolled and overexcited cell differentiation and division mechanisms with the possibility to spread to or invade other parts of the body^1^. As a result, research work into anticancer medications that are highly effective and with minimal toxicity is still an important trend in anticancer drug research and development^2^^,^^3^. In this manner, many recent strategies targeting specific enzymes and/or biomarkers required for cancer cell proliferation and/or to control apoptosis such as mutated, deregulated, or overexpressed proteins^4^ and thus, specifically affect cancer cells and/or their propping environment with the least effects on normal cells, attract major attention^5^. Among these targets are the vascular endothelial growth factor receptor-2 (VEGFR-2) which is one of the key intermediates in tumour angiogenesis^6^, and the anti-apoptotic and pro-apoptotic proteins that regulate the cellular apoptosis^7–9^.

Cancer cells need oxygen and nutrients to survive and proliferate; hence they must be near blood vessels to have accessibility to the blood circulation system^10^. Angiogenesis, the production of new blood capillaries from already existing vessels, is therefore an essential part in cancer growth and proliferation^11–13^. Accordingly, blocking angiogenesis through several methods including VEGFR-2 inhibition has proved significant effectiveness in cancer therapy^6^. Many studies have shown that inhibiting the VEGFR-2 or minimising its response is an efficient method in the assessment of new drugs for treatment of several cancer types^14–17^.

Apoptosis, a mechanism of programmed cell death in multicellular organisms, is a chain of biochemical reactions that results in specific cell changes and cell death^18^. One of the main pathways of cell apoptosis induction is the mitochondria-dependent apoptotic pathway which is regulated by the B-cell lymphoma-2 (Bcl-2) protein family^19^^,^^20^. The Bcl-2 different family members could express opposite functions; some are pro-apoptotic proteins such as Bac and Bax, the two nuclear-encoded proteins that promote cell apoptosis, while others are anti-apoptotic proteins, such as Bcl-2 and Bcl-xL that inhibit cell apoptosis^9^. In this concern, it was reported that many cancer cells are characterised by the anti-apoptotic Bcl-2 protein overexpression that leads to apoptosis prevention as well as drug resistance^21^^,^^22^. Therefore, the production of Bcl-2 proteins inhibitors has become a significant target for introducing promising anti-cancer agents^23^^,^^24^.

**Figure 1.**
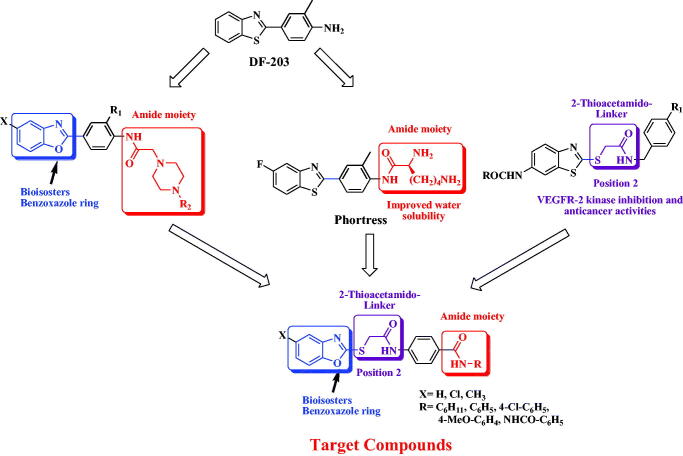
Design of the target benzoxazole-benzamide conjugates **1–15**.

Recently, numerous small molecules bearing diversified heterocyclic scaffolds have been proved as potential anticancer agents *via* different mechanisms, including inhibition of angiogenesis and/or cell apoptosis induction^17^. For the meantime, the bicyclic isosteric scaffolds, namely; benzothiazole, benzoxazole and benzimidazole are considered as vital leads for many pharmacological activities including anti-inflammatory^25–29^, antiviral^30–33^, and mainly antitumor^34–45^. As a privileged scaffold, benzothiazole was the main nucleus for several compounds, such as compound **DF-203** (Figure 1) that showed significant *in-vitro* anticancer activities. However, its low solubility was the main issue for further *in-vivo* investigation^35^. **Phortress**, a water-soluble analog bearing an amino-acid moiety and displaying both strong and selective anti-cancer activity was developed to overcome these solubility difficulties^35^ (Figure 1). An additional modification was conducted *via* replacing the benzothiazole ring with its benzoxazole bioisoster, which led to promising anticancer agents^43^ (Figure 1). On the other hand, analogs with two aryl moieties separated with a 2-thioacetamido linker have been reported to have VEGFR-2 kinase inhibition, antitumor activity and improved aqueous solubility comparable to their lead compound^44^.

**Figure 2.**
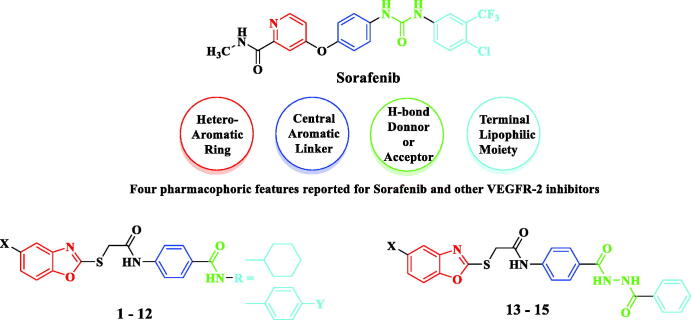
Target benzoxazoles fulfilled the pharmacophoric structural features of VEGFR-2 inhibitors.

Moreover, the novel benzoxazole series was designed to meet the four main pharmacophoric features reported for sorafenib and other VEGFR-2 inhibitors^46–48^. As illustrated in Figure 2 the proposed benzoxazole derivatives (**1–15**) exhibit pharmacophoric features similar to sorafenib, where the terminal benzoxazole ring could occupy the hinge region of the ATP binding site^47^. Also, the central aromatic benzene ring linked *via* a 2-thioacetamido group could occupy the area between the hinge region and the DFG domain of the activation loop^49^. In addition, the amide or diamide groups could act as H-bond donors and/or acceptors^50^ and finally, the cyclohexyl or phenyl ring represents the terminal hydrophobic moiety that could occupy the allosteric hydrophobic pocket through several hydrophobic interactions^51^ (Figure 2).

Considering the aforementioned findings, our group designed and synthesised a new series of benzoxazole-benzamide conjugates linked *via* a 2-thioacetamido moiety. All targeted compounds were evaluated *in-vitro* for their anticancer activity against both human breast (MCF-7) and colorectal (HCT-116) cancer cell lines and compared with their cytotoxicity in normal human fibroblasts (WI-38). For further investigation of the potential anticancer mechanism of the synthesised compounds, VEGFR enzymatic inhibition potential was determined, followed by DNA cell cycle analysis for the most active compounds. In addition, the ability of these conjugates to induce cell apoptosis was tested. The level of mitochondrial anti-apoptotic protein Bcl-2 and Bcl-xL in both HCT-116 and MCF-7 cancer cell lines was determined. Finally, molecular docking studies were performed for the synthesised compounds against VEGFR (PDB ID: 4ASD) with sorafenib as a reference ligand.

## 2. Results and discussion

### 2.1. Chemistry

**Scheme 1:**
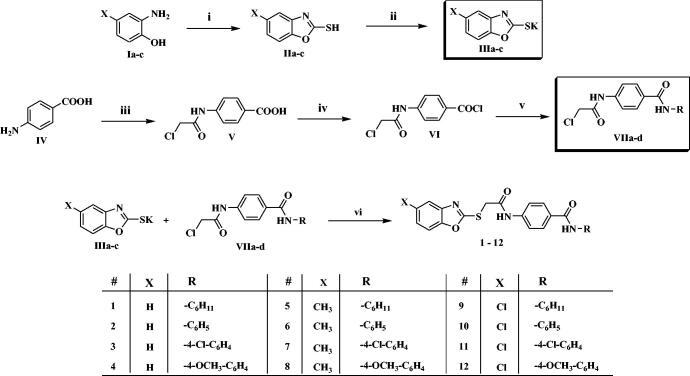
Synthesis of the compounds **1–12**; Reagents/conditions: (i) CS_2_/KOH/CH_3_OH/reflux 6 h, (ii) KOH/C_2_H_5_OH/reflux 4 h, (iii) ClCH_2_COCl, NaHCO_3_/DMF/r.t./1h, (iv) SOCl_2_/1,2-dichloroethane/reflux 4 h, (v) R-NH_2_/acetonitrile/TEA/r.t. 8 h, (vi) DMF/KI/60 °C/6h.

**Scheme 2.**
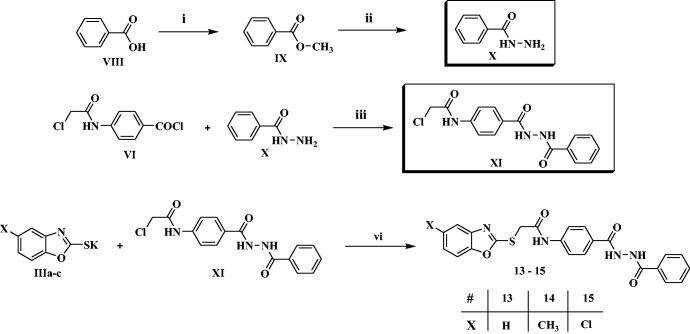
Synthesis of the compounds **13–15**; Reagents/conditions: (i) CH_3_OH/conc. H_2_SO_4_/reflux 2 h, (ii) NH_2_-NH_2_/C_2_H_5_OH/reflux 4 h, (iii) acetonitrile/TEA/r.t. 8 h, (vi) DMF/KI/60 °C/6h.

Benzoxazole derivatives **1–15** were synthesised following the general methodologies outlined in Schemes 1 and 2. The key starting materials, 2-mercaptobenzoxazoles **IIa-c** were synthesised by refluxing the corresponding 2-aminophenol derivatives **Ia-c**, carbon disulphide, and potassium hydroxide in methanol, according to the reported procedure^52^. Then, compounds **IIa-c** were treated with alcoholic KOH to give the corresponding potassium salts, **IIIa-c** (Scheme 1). On the other hand, 4-aminobenzoic acid **IV** was reacted with chloroacetyl chloride in DMF to afford the chloroacetamide intermediate **V**. Then, treatment of compound **V** by thionyl chloride afforded 4-(2-chloroacetamido)benzoyl chloride **VI**^53^^,^^54^, which was then successively reacted with a set of commercially available amines namely, cyclohexylamine, aniline, 4-chloroaniline, 4-methoxyaniline in acetonitrile and triethylamine (TEA), to get the key intermediates **VIIa-d**. Finally, compounds **VIIa-d** were heated with the formerly prepared potassium salts **IIIa-c** in dry DMF to afford the final target compounds **1–12** (Scheme 1).

On the other hand, methylbenzoate **IX** was prepared as reported, by refluxing benzoic acid **VIII** in methanol in presence of sulphuric acid^55^^,^^56^. Then, refluxing of **IX** with hydrazine hydrate afforded the corresponding acid hydrazide **X**^57^, which was further acylated by **VI** in acetonitrile and TEA to afford the corresponding derivative **XI**. As previously, compound **IX** was finally heated with the formerly prepared potassium salts **IIIa-c** in dry DMF to afford the final target compounds **13–15** (Scheme 2).

The proposed structures of the final conjugates reported here were in full agreement with their elemental and spectral analysis data. IR spectra of all compounds displayed the absorption bands for the (NH) and (C=O) groups in the 3356–3273 and 1673–1598 cm^−1^ regions, respectively. Also, compounds **1**, **5** and **9** showed additional C-H stretching bands at 2933–2927 cm^−1^, due to the presence of the aliphatic cyclohexyl group. In addition, ^1^HNMR spectra for compounds **1**, **5** and **9** displayed two signals exchangeable with D_2_O, referable to the two amidic NH groups at chemical shifts of *δ* 10.61–10.63 *ppm* and at *δ* 8.08 *ppm* for the acetamido group and benzamido group, respectively. For the remaining compounds, the signals of the two amidic NH groups were in the range of *δ* 10.68–10.73 *ppm* and at *δ* 10.00–10.24 *ppm* for the acetamido group and benzamido group, respectively. On the other hand, ^1^HNMR spectra displayed the presence of a singlet peak for the methylene protons of the 2-thioacetamido linker at *δ* 4.37–4.43 *ppm*, whereas compounds **5**–**8** revealed another singlet peak in the aliphatic region referable to the methyl group at *δ* 2.37–2.28 *ppm*. Moreover, compounds **4**, **8** and **12** displayed an extra singlet signal for the methoxy group at *δ* 3.72 *ppm*.

Also, the structures of compounds **13**–**15** were confirmed by their spectral and elemental analyses. The ^1^HNMR spectra for compounds **13**–**15** displayed three singlet signals exchangeable with D_2_O, one for the acetamido group in the range of *δ*10.70–10.72 *ppm*, and two for the acyl hydrazide group at *δ*10.44 *ppm* and *δ*10.39 *ppm*. Additionally, the spectra showed a singlet signal for the methylene protons of the 2-thioacetamido linker in the range of *δ* 4.40–4.42 *ppm* for compounds **13**–**15** and a singlet signal attributed to the methyl group at *δ* 2.39 *ppm* for compound **14**.

### 2.2. Biological evaluation

#### 2.2.1. Anti-proliferative activity against HCT-116 and MCF-7 human cancer cells lines

**Figure 3.**
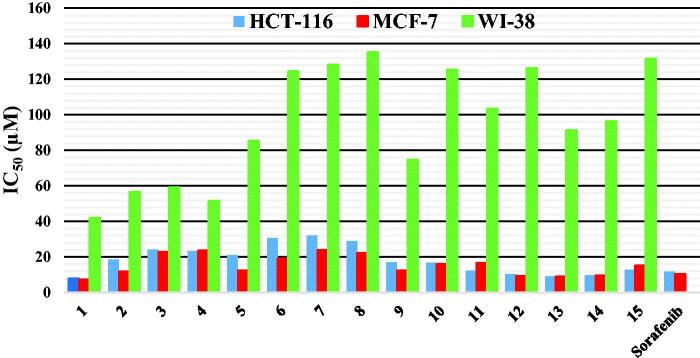
*In vitro* anti-proliferative activity of the target compounds **1–15**.

Recently, benzoxazole derivatives have attracted more attention in drug design, and notably to access compounds with anticancer activity. Several of these derivatives were reported as acting as competitive inhibitors of different tyrosine kinases, with potent cytotoxic activity against various cell lines^58^^,^^59^. Other series of benzoxazole derivatives showed significant potency against colon and breast cancer cell lines and their activity was explained by the potent inhibition of VEGFR enzymes^60–62^. Thus, in this study a novel series of benzoxazole-benzamide conjugates was initially evaluated for their potential anti-cancer activity against colon cancer cell line (HCT-116), breast cancer cell line (MCF-7) and normal human fibroblasts (WI-38), using the Sulforhodamine B colorimetric (SRB) assay^63^. Sorafenib as an FDA approved VEGFR-2 inhibitor was utilised as a positive reference compound. The cytotoxic activities were displayed in Table 1 and Figure 3 and expressed as the median growth inhibitory concentration (IC_50_).

**Table 1.** *In vitro* anti-proliferative activity of the compounds **1–15** against HCT-116, MCF-7 human cancer cell lines and W-180 normal cell line, and their corresponding selectivity indices.

Compounds	HCT-116		MCF-7		WI-38
	IC_50_ (µM)^a^	SI^b^	IC_50_ (µM)^a^	SI^b^	IC_50_ (µM)^a^
**1**	7.8 ± 0.015	5.3	7.2 ± 0.010	5.8	41.9 ± 0.27
**2**	18.5 ± 0.014	3.0	11.7 ± 0.014	4.8	56.7 ± 0.36
**3**	24.2 ± 0.019	2.4	22.7 ± 0.008	2.6	59.3 ± 0.45
**4**	23.2 ± 0.012	2.2	23.6 ± 0.015	2.2	51.5 ± 0.40
**5**	20.9 ± 0.025	4.0	12.4 ± 0.007	6.9	85.4 ± 0.55
**6**	30.7 ± 0.011	4.0	19.1 ± 0.006	6.5	124.6 ± 0.70
**7**	32 ± 0.002	4.0	24 ± 0.011	5.3	128.2 ± 0.75
**8**	28.8 ± 0.010	4.7	22.3 ± 0.004	6.0	135.4 ± 0.85
**9**	17.1 ± 0.008	4.4	12.3 ± 0.008	6.0	74.6 ± 0.53
**10**	16.7 ± 0.012	7.5	16.1 ± 0.011	7.8	125.5 ± 0.72
**11**	12.2 ± 0.007	8.5	16.6 ± 0.013	6.2	103.5 ± 0.70
**12**	10.4 ± 0.010	12.1	9.4 ± 0.016	13.4	126.2 ± 0.75
**13**	9.1 ± 0.005	10.0	9.0 ± 0.005	10.1	91.3 ± 0.60
**14**	9.7 ± 0.013	9.9	9.5 ± 0.009	10.1	96.5 ± 0.65
**15**	12.9 ± 0.014	10.2	15.3 ± 0.01	8.6	131.5 ± 0.80
**Sorafenib**	11.6 ± 0.012	–	10.5 ± 0.014	–	–

**^a^**IC_50_ values are the mean ± SD of three separate experiments.

^b^Selectivity index (SI) is the ratio of the IC_50_ value for normal cells (WI-38) to the IC_50_ values for HCT-116 and MCF-7 cells.

Analysing results towards both HCT-116 and MCF-7 cell lines revealed that generally compounds bearing a 5-chlorobenzoxazole moiety (**9–12** and **15**) showed better cytotoxic activity than their 5-methyl (compounds **5–8** and **14**) or their unsubstituted benzoxazole analogs (compounds **2–4** and **13**), with the exception of compound **1**, bearing an unsubstituted benzoxazole moiety and a cyclohexyl group in its amidic side, and which displayed the best inhibitory activity of these two series, with IC_50_ values of 7.2 ± 0.01 µM and 7.8 ± 0.015 µM against HCT-116 and MCF7 cell lines, respectively. Concerning the influence of the amide group, a cyclohexyl substituent led globally to more active compounds than a phenyl or a substituted phenyl group (compared compounds **1** to **2**–**4** or **5** to **6**–**8** or **9** to **10**), except for compound **12** bearing a 4-methoxybenzamide group, which was more active than its cyclohexyl analog **9**. Moreover, it is worthy to mention that generally the acyl hydrazide derivatives **13–15** showed higher inhibitory activity than their benzamide analogs towards both cancer cell lines. Thus, as an example, compound **13** showed an IC_50_ of 9.1 ± 0.005 µM against HCT-116 cells, compared to an IC_50_ of 18.5 ± 0.005 µM for compound **2**.

In addition, results against the MCF-7 cell line showed that compounds **1** and **12**–**14** exhibited excellent activity with single-digit micromolar IC_50_ values ranged between 7.2 ± 0.01 and 9.5 ± 0.009 µM, more potent than the reference drug, sorafenib. While compounds **2**, **5** and **9** showed good potency with IC_50_ of 11.7 ± 0.014−12.4 ± 0.007 µM, the remaining compounds had moderate to weak cytotoxic activity with IC_50_ of 15.3 ± 0.01−24.0 ± 0.011 µM. In a similar way, compounds **1**, **12–14** showed a single digit micromolar IC_50_ values against HCT-116 cells (IC_50_ range: 7.8 ± 0.015−10.4 ± 0.01 µM), higher than sorafenib that possessed IC_50_ value of 11.6 ± 1.00 µM. On the other hand, while compounds **11** and **15** showed good potency with IC_50_ values of 12.2 ± 0.007−12.9 ± 0.014 µM, the remaining compounds had moderate to weak cytotoxic activity with IC_50_ range of 16.7 ± 0.012−32.0 ± 0.002 µM.

Finally, all tested compounds showed weak cytotoxicity against normal human fibroblasts (WI-38), with IC_50_ range of 42−135 µM, representing a selectivity index of 2.2 to 13.4, compared to IC_50_ values against both HCT-116 and MCF-7 cancer cell lines.

These results revealed that some of the novel benzoxazole compounds are very promising candidates as relatively safe cytotoxic agents. Thus, the most active derivatives were submitted for further investigations regarding their potential anti-proliferative mode of action.

#### 2.2.2. VEGFR-2 inhibitory activity

**Figure 4.**
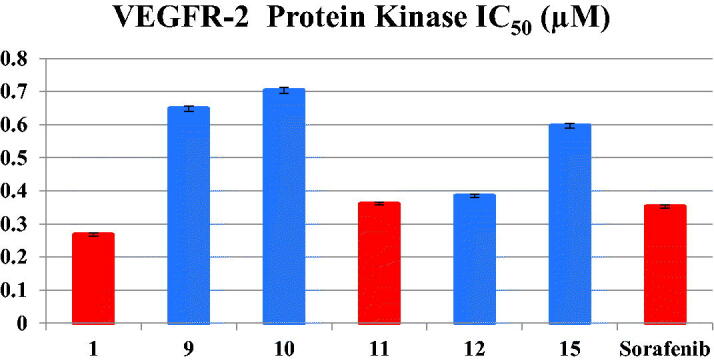
Inhibitory activity of **1, 9, 10, 11, 12** and **15** against VEGR-2 Protein Kinase.

The excellent cytotoxic effects of several benzoxazole derivatives, in particular compound **1**, motivated a further exploration of their potential inhibitory activities against VEGFR-2 protein kinase. Representative compounds **1**, **9**–**12** and **15** were selected to determine their potential inhibitory activity. As presented in Table 2 and Figure 4, the results revealed that all examined compounds exhibited sub-micromolar IC_50_ values of VEGFR-2 inhibitory activity. Among all tested compounds, the unsubstituted benzoxazole compound **1**, bearing a cyclohexyl group in the amidic side, was the best inhibitor of VEGFR-2 activity with IC_50_ value of 0.268 µM, more potent than the clinically used kinase inhibitor, sorafenib, which exhibited IC_50_ value of 0.352 µM, followed by conjugates **11** and **12** with comparable IC_50_ values of 0.361 µM and 0.385 µM, respectively. On the other hand, compounds **9**, **10** and **15** exhibited the least inhibitory activity with IC_50_ value ranged from 0.597 to 0.704 µM. Finally, the presented results revealed that the VEGFR-2 inhibitory activities were in excellent match with the cytotoxic activities of compounds **1**, **11** and **12** suggesting that the anti-proliferative activity might be attributable to VEGFR-2 enzyme inhibition.

**Table 2.** Inhibitory activity of **1, 9, 10, 11, 12** and **15** against VEGR-2 Protein Kinase.

No.	VEGFR-2 Protein Kinase IC_50_ (µM)
**1**	0.268 ± 0.005
**9**	0.649 ± 0.008
**10**	0.704 ± 0.009
**11**	0.361 ± 0.004
**12**	0.385 ± 0.005
**15**	0.597 ± 0.007
**Sorafenib**	0.352 ± 0.005

IC_50_ values are the mean of three individual experiments.

#### 2.2.3. Cell cycle analysis

**Figure 5.**
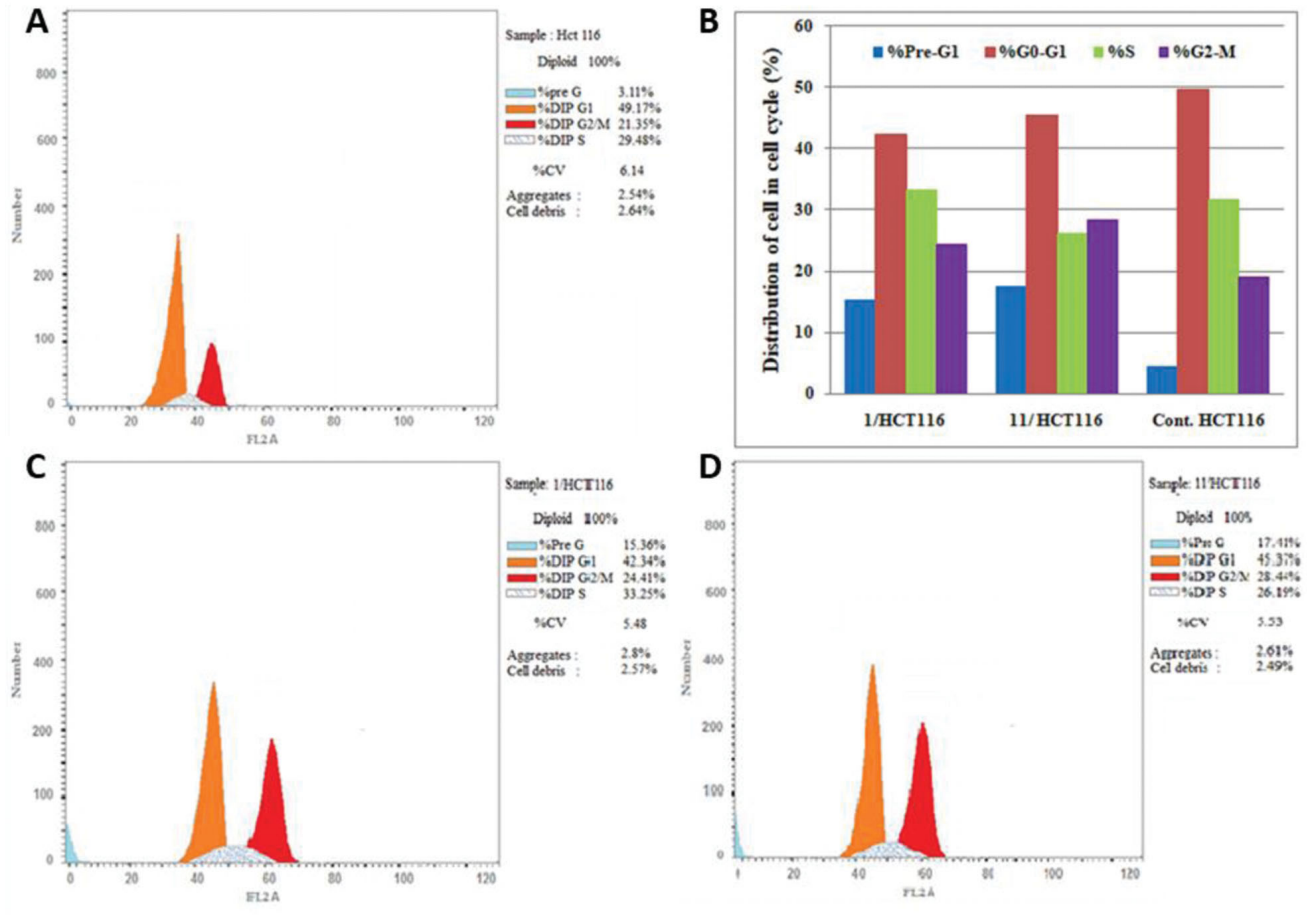
Cell distribution in the subG1, G0/G1, S and G2/M phases for HCT116 cells (**B**) treated with vehicle control (**A**), compounds **1** (**C**) and **11** (**D**).

**Figure 6.**
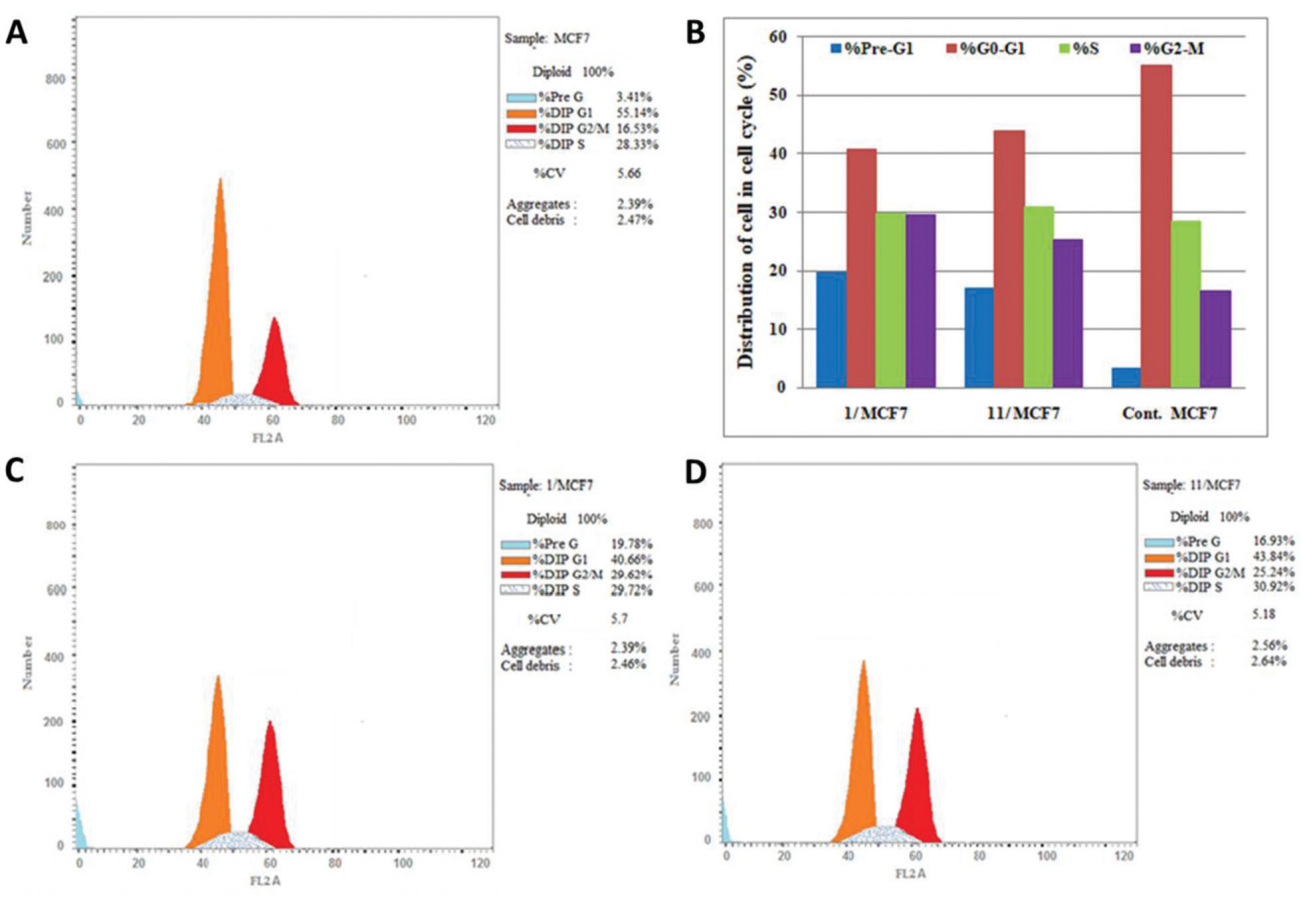
Cell distribution in the subG1, G0/G1, S and G2/M phases for MCF7 cells (**B**) treated with vehicle control (**A**), compounds **1** (**C**) and **11** (**D**).

It is clearly known that generally the cytotoxic agents exert their anti-proliferative effect *via* cell cycle arrest at a specific phase. In the present study, due to the excellent *in-vitro* anti-proliferative activity of conjugates **1** and **11** against both HCT-116 and MCF-7 cancer cell lines as well as their excellent VEGFR-2 inhibitory activities, cell cycle analysis have been carried out for both compounds. The effect of compounds **1** and **11** on the cell cycle progression in order to determine the phase at which cell cycle arrest takes place in both cancer cell lines was evaluated by a DNA flow cytometry analysis, upon incubation of HCT-116 and MCF-7 cancer cell lines with compounds **1** and **11** at their IC_50_ concentrations for 24 h (Table 3 and Figures 5 and 6).

**Table 3.** Effect of compounds **1**, **11** and vehicle control on the cell cycle phases of HCT-116 and MCF-7 cells lines.

Compound / Cell line	%G0-G1	%S	%G2-M	%Pre-G1
**1 / HCT116**	42.34	33.25	24.41	15.36
**11 / HCT116**	45.37	26.19	28.44	17.41
**Control / HCT116**	49.51	31.56	18.93	4.52
**1 / MCF7**	40.66	29.72	29.62	19.78
**11 / MCF7**	43.84	30.92	25.24	16.93
**Control / MCF7**	55.14	28.33	16.53	3.41

The results showed that, for HCT-116 cancer cells lines, the percentage of cells at G2/M phase relatively increased from 18.93% in control to 24.41% and 28.44% after incubation with compounds **1** and **11**, respectively. In addition, the percentage of HCT-116 cells in G1 phase was decreased from 49.51% to 42.34% for compound **1** and 45.37% for compound **11** (Figure 5).

Similarly, the results revealed that, for MCF-7 cancer cell line, the percentage of cells in the G2/M phase was significantly increased from 16.53% to 29.62% for compound **1** and 25.24% for compound **11**. In addition, the percentage of MCF-7 cells at G1 phase decreased from 55.14% in control to 40.66% and 43.84% after incubation with compounds **1** and **11**, respectively (Figure 6). These results indicated that compounds **1** and **11** induced cell cycle arrest at G2/M phase. Finally, the upsurge of cell populations in the pre-G1 phase along with the G2-M phase arrest were significant evidence that compounds **1** and **11** induced apoptosis in both HCT-116 and MCF-7 cancer cell lines.

#### 2.2.4. Annexin V-FITC/PI apoptosis test

**Figure 7.**
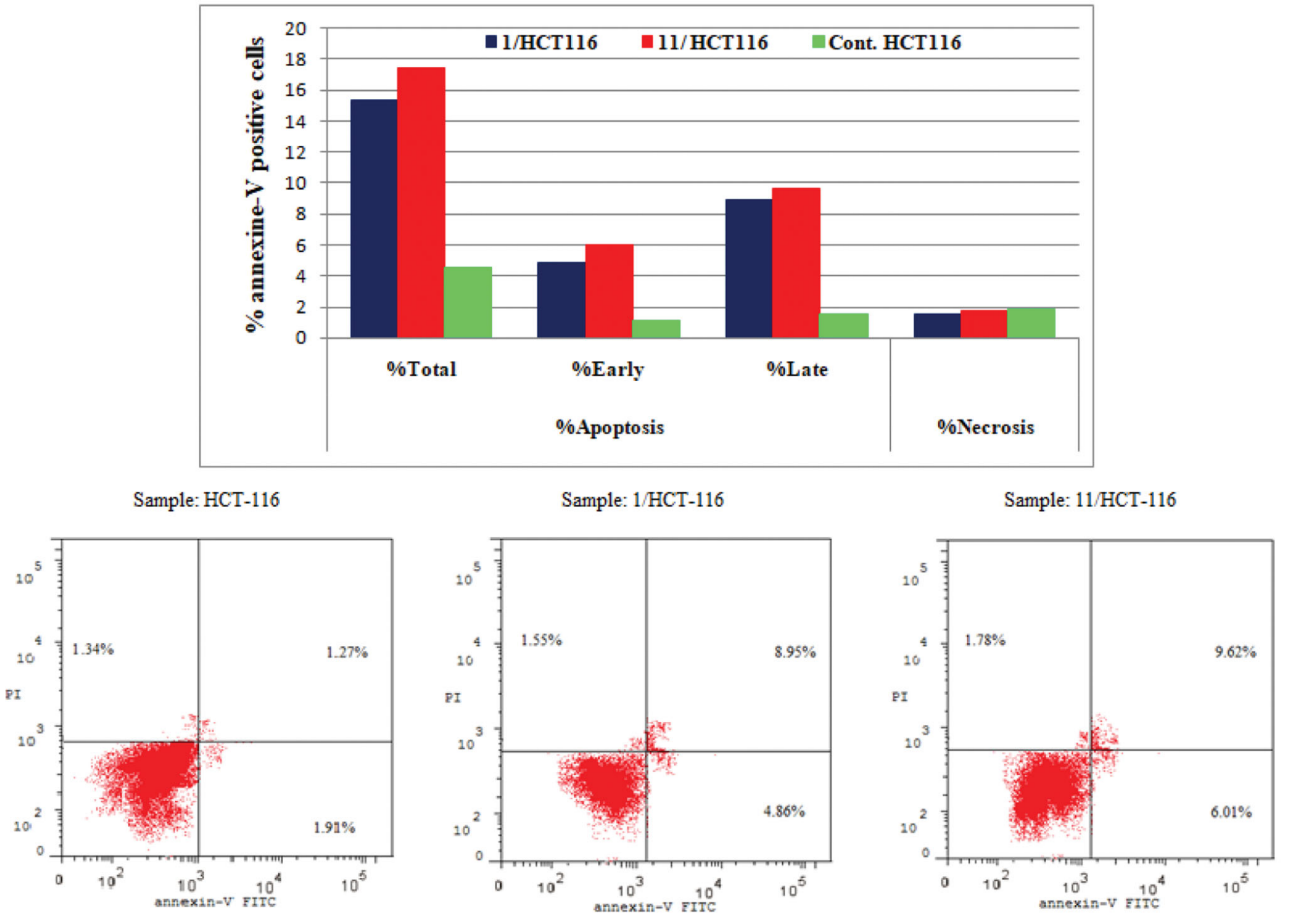
Effect of compounds **1**, **11** and vehicle control on the percentage of annexin V-FITC-positive staining in HCT-116 cell line. The experiments were done in triplicates. The four quadrants identified as: LL, viable; LR, early apoptotic; UR, late apoptotic; UL, necrotic.

**Figure 8.**
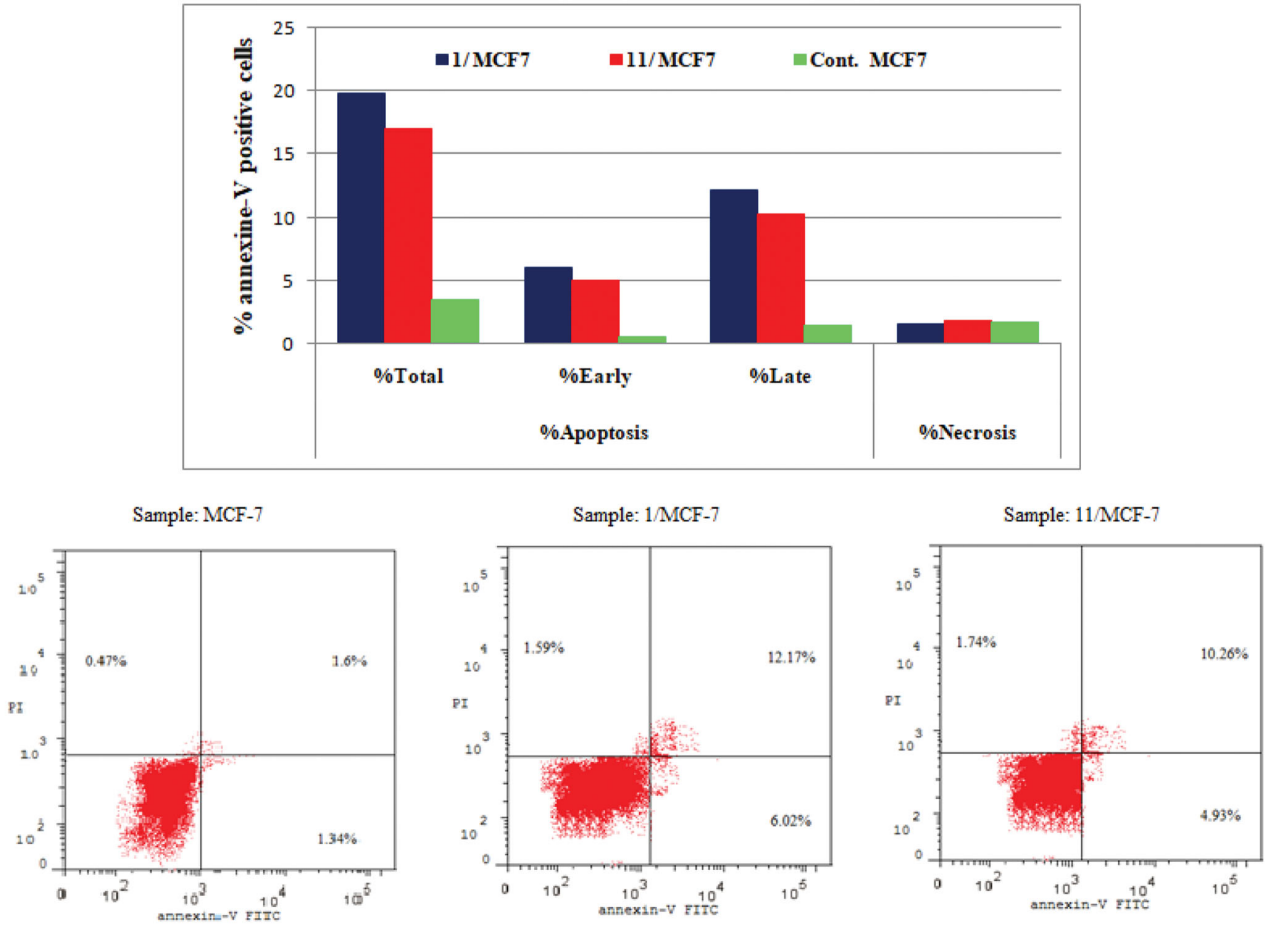
Effect of compounds **1**, **11** and vehicle control on the percentage of annexin V-FITC-positive staining in MCF-7 cell lines. The experiments were done in triplicates.

To determine whether the growth inhibitory action of compounds **1** and **11** is consistent with the induction of apoptosis suggested by the elevated population of pre-G1 in the treated HCT-116 and MCF-7 cells, Annexin V-FITC/PI double staining (AV/PI) apoptosis assay was carried out. The results of this assay revealed that compounds **1** and **11** induced both early and late apoptosis in both HCT-116 and MCF-7 cell lines and the results were outlined in Table 4 and Figures 7 and 8.

**Table 4.** Percent of apoptosis and necrosis induced by compounds **1**, **11** and vehicle control in HCT-116 and MCF-7 cell lines.

Compound / Cell line	%Apoptosis			%Necrosis
	%Total	%Early	%Late	
**1 / HCT-116**	15.36	4.86	8.95	1.55
**11 / HCT-116**	17.41	6.01	9.62	1.78
**Control / HCT-116**	4.52	1.1	1.57	1.85
**1 / MCF-7**	19.78	6.02	12.17	1.59
**11 / MCF-7**	16.93	4.93	10.26	1.74
**Control / MCF-7**	3.41	0.47	1.34	1.6

The results revealed that treatment of HCT-116 cells with compound **1** and **11** resulted in an increase in the apoptotic cells percentage for the early apoptosis, from 1.1% for control untreated cells to 4.89% and 6.01%, respectively. In addition, the percentage of apoptotic cells in the late stage was 8.95% to 9.62% compared to control (1.57%). These results revealed that compounds **1** and **11** were able to induce an approximately 3.4-folds and 3.9-folds, respectively, increase in total apoptosis compared to the control for HCT-116 cell line (Figure 7).

On the other hand, for MCF-7 cancer cell line, the results showed that conjugates **1** and **11** led to an increase in the apoptotic cells percentage for the early apoptosis, from 0.47% for control untreated cells to 6.02% and 4.93%, respectively. In addition, for the late stage, the percentage of apoptotic cells increased from 1.34% for control cells to 12.17% and 10.26%, for compounds **1** and **11,** respectively. These results showed that the tested compounds were able to induce an approximately 5.8-fold and 5.0-fold total increase in apoptosis compared to the control, for compounds **1** and **11** respectively (Figure 8). These results persuaded us to further investigate the effect of compound **1** and **11** on mitochondrial anti-apoptotic biomarkers Bcl-2 and Bcl-xL.

#### 2.2.5. Impact of compounds 1 and 11 on the level of Bcl-2 and Bcl-xL

**Figure 9.**
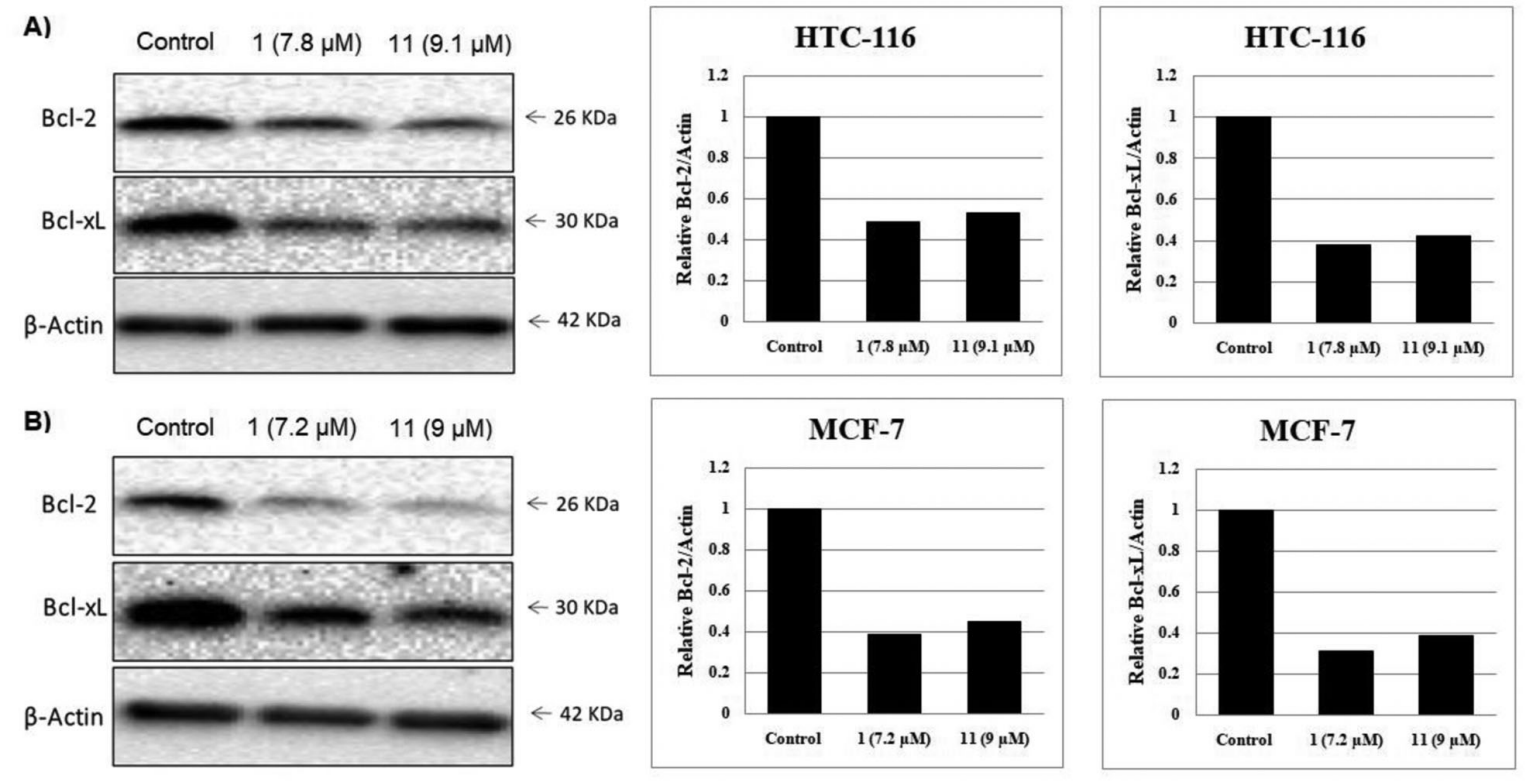
Effect of compounds **1**, **11** and vehicle control on anti-apoptotic proteins (Bcl-2 and Bcl-xL) in (**A**) HCT-116 cancer cells and (**B**) MCF-7 cancer cells.

It is well known that the anti-apoptotic proteins Bcl-2 and Bcl-xL are mainly overexpressed in various types of cancer, causing survival of cancer cells and/or drug resistance^7–9^. Therefore, inhibition of these proteins expression leads to cancer cell death and has been used as a strategy for anticancer drug development^23^. In this study, the impact of compounds **1** and **11** on Bcl-2 and Bcl-xL expression in HCT-116 and MCF-7 cancer cell lines was examined using Western blot analysis and all the data were normalised to β-actin (Figure 9). The presented results revealed that benzoxazoles **1** and **11** inhibited Bcl-2 and Bcl-xL expression in both HCT-116 and MCF-7 cancer cell lines in a corresponding manner to their cytotoxic activity and their apoptosis induction ability.

#### 2.2.6. Molecular docking

Molecular docking studies are considered as an influential method for interpretation of molecular interactions between the synthesised compounds and the main amino acid residues at the specific binding site of the target receptor^64^. The activity of the newly synthesised ligands and VEGFR protein interactions at the active binding site was compared according to the docking score values calculated using MOE 2015.10. In the current work, all the synthesised benzoxazole compounds were put through molecular docking studies using MOE software on the VEGFR 3D-structure and using sorafenib as a reference ligand.

**Figure 10.**
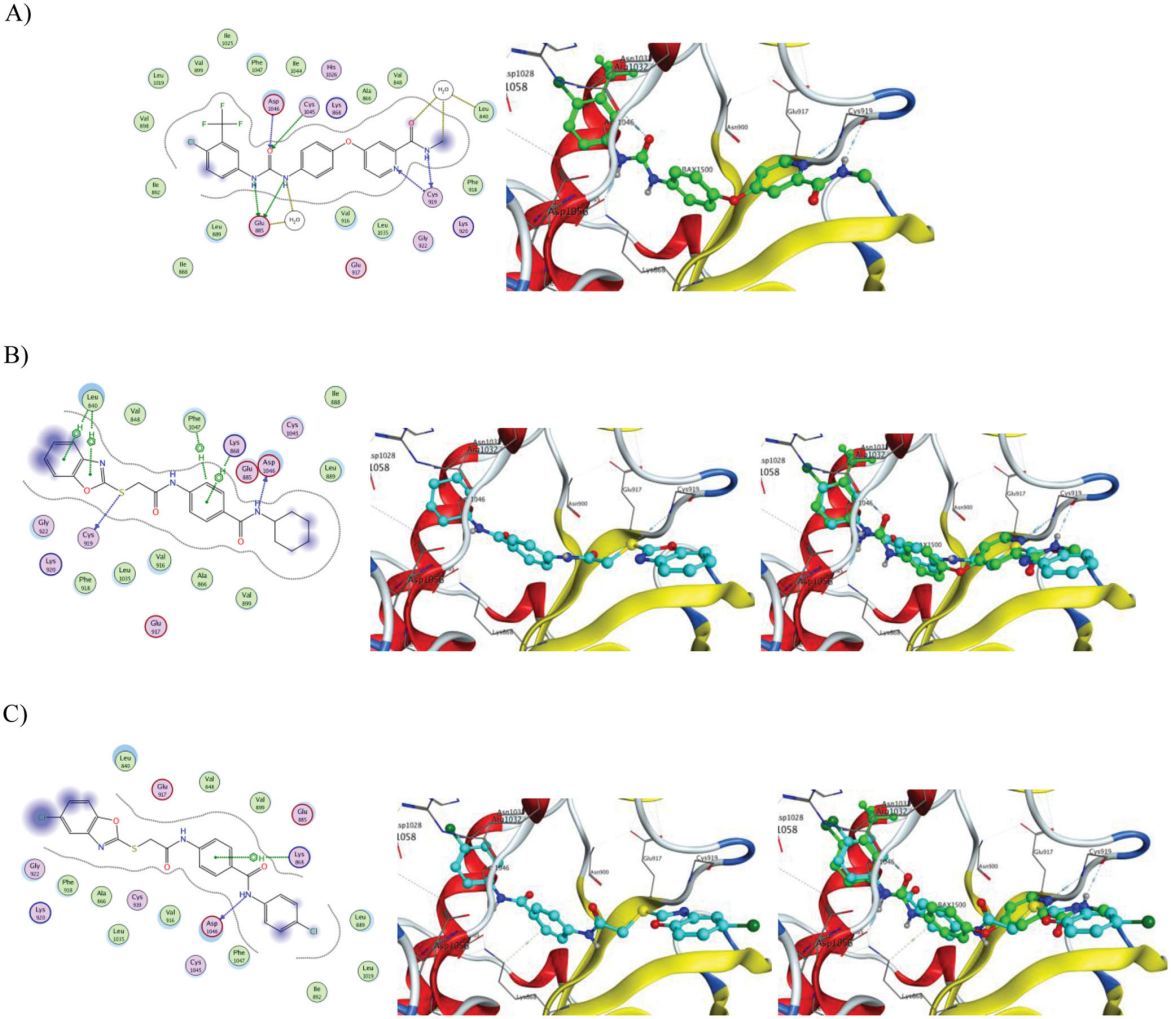
Docking of compounds **1**, **11** and sorafenib into the VEGFR active site. (A) Interaction of Sorafenib with amino-acids Leu840, Glu885, Lys920 and Asp1046. (B) Interaction of **1** with amino-acids Leu840, Lys868, Cys919, Asp1046 and Phe1047 and superimposition of **1** (shown as cyan sticks) with sorafenib (shown as green sticks). (C) Interaction of **11** with amino-acids Lys868 and Asp1046 and superimposition of **11** (shown as cyan sticks) with sorafenib (shown as green sticks).

The results revealed that, the most biologically active compounds **1** and **11** displayed an excellent docking score (−8.45083 kcal/mol and −8.15044 kcal/mol, respectively) compared to sorafenib docking score (−6.98449 kcal/mol), and both compounds formed direct interactions with many of the amino-acids that sorafenib interacted with (Table 5). As shown in Figure 10, sorafenib had direct interactions with amino-acids Leu840, Glu885, Lys920 and Asp1046 in the active site (Figure 10(A)). Compound **1** shared sorafenib interactions with amino-acids Leu840 and Asp1046, and additionally exhibited other interactions with amino-acids Lys868, Cys919 and Phe1047 (Figure 10(B)). On the other hand, compound **11** shared the interaction with only Asp1046 and showed another interaction with amino-acid Lys868 (Figure 10(C)). Also, compounds **1** and **11** displayed high degrees of superimposition with sorafenib into the VEGFR active site (Figure 10(B,C)). Finally, the more interaction formed with amino-acids at the active site by compound **1** than compound **11** strongly support the results of VEGFR enzyme inhibition assay where compound **1** was the most active with IC_50_ of 0.268 µM compared to that of compound **11** and sorafenib that had IC_50s_ of 0.361 µM and 0.352 µM, respectively (Table 2).

**Table 5.** Docking energy scores (kcal/mol) obtained from the MOE software for compounds **1–15** and sorafenib.

Compound No.	Score	rmsd_refine	E_conf	E_place	E_score1	E_refine	E_score2
**1**	−8.45083	3.46348	7.13944	−61.18144	−10.73645	−15.61404	−8.450831
**2**	−8.07490	4.14820	24.33731	−65.02733	−11.56395	−16.29065	−8.074901
**3**	−7.18548	1.39598	29.55076	−82.09853	−11.23328	−8.114554	−7.185479
**4**	−8.41887	1.47159	27.91452	−89.37426	−11.65076	−19.99584	−8.418868
**5**	−7.63854	2.33253	2.78024	−88.87581	−12.35492	−4.167041	−7.638539
**6**	−7.88400	1.76917	20.26365	−77.02324	−11.26872	−15.04636	−7.884004
**7**	−9.36350	1.7113	17.05548	−84.28175	−11.35441	−22.20009	−9.363497
**8**	−9.38409	1.46039	21.96994	−79.49125	−12.83700	−22.23286	−9.384088
**9**	−7.93492	0.97358	−1.62790	−108.53313	−12.54650	−5.27634	−7.934922
**10**	−8.69645	2.34028	9.40272	−69.44931	−11.03539	−21.76292	−8.696453
**11**	−8.15044	3.47698	16.16184	−104.55356	−11.93168	−16.78374	−8.150444
**12**	−9.30653	2.37350	11.61155	−100.80922	−11.70867	−21.75587	−9.306530
**13**	−8.82812	1.14095	63.19030	−79.27531	−10.92340	−23.19646	−8.828117
**14**	−8.71854	2.22981	70.10210	−86.90809	−10.84697	−21.77860	−8.718533
**15**	−9.27842	1.94674	53.63672	−69.43458	−10.36066	−21.94845	−9.278418
**Sorafenib**	−6.98449	1.78143	−3.12607	−79.69516	−10.28793	−7.641235	−6.984490

**Score**: lower scores are more favourable; **rmsd_refine**: the root mean square deviation of the pose; **E_conf**: free binding energy (**FBE**) of the conformer; **E_place**: **FBE** from the placement stage; **E_score 1**: **FBE** from the first rescoring stage; **E_refine**: **FBE** from the refinement stage; **E_score 2**: **FBE** from the second rescoring stage.

## 3. Conclusions

In the current study, a novel series of novel benzoxazole-benzamide conjugates linked *via* a 2-thioacetamido group (**1−15**) was designed and synthesised as potential anti-cancer agents with probable inhibitory activity on the VEGFR-2 enzyme and on the expression of anti-apoptotic Bcl-2 and Bcl-xL proteins. The tested compounds were relatively safe against normal human fibroblasts (WI-38) and the cell proliferation of two examined cancer cell lines (HCT-116 and MCF-7) has been notably inhibited by all synthesised compounds with IC_50_ ranges from 7.8 to 32.0 µM against HCT-116 and from 7.2 to 24.0 µM against MCF-7, as compared to IC_50_ of 11.6 and 10.5 µM for sorafenib, respectively. In addition, compounds **1, 9, 10, 11, 12** and **15** showed excellent VEGFR-2 inhibitory activity. In particular, benzoxazoles **1** and **11** revealed to be slightly more or equally potent than sorafenib, with IC_50_ of 0.27, 0.36 µM and 0.35 µM, respectively. Moreover, docking studies showed that the compounds are positioned in a very similar manner to sorafenib into the VEGFR active site. Further mechanistic studies showed that compounds **1** and **11** induced apoptosis and inhibited the expression of anti-apoptotic Bcl-2 and Bcl-xL proteins in both HCT-116 and MCF-7 cancer cell lines. Finally, the high potency of this benzoxazole series suggested that conjugates **1** and **11** could avail as lead compounds for further investigation and optimisation to develop novel anti-proliferative agents, apoptotic inducers and inhibitors of Bcl-2/Bcl-xL expression.

## 4. Experimental

### 4.1. Chemistry

#### 4.1.1. General

Melting points (°C) of the synthesised compounds were uncorrected and were measured using Electrothermal Stuart 5MP3. Follow-up of reactions was performed using TLC plates of silica gel 60 F254 (Merck). The NMR spectrometric analyses have been recorded using Bruker-Avance 400 NMR spectrometer (400 MHz for ^1^HNMR and100 MHz for ^13^CNMR) in deuterated dimethylsulphoxide (DMSO-*d6*). Chemical shifts (*δ_H_*) were reported relative to the solvent (DMSO-*d_6_*). Mass spectra were recorded on Finnigan Mat SSQ 7000 mode EI 70 eV at the micro analytical unit, Cairo University, Cairo, Egypt. Schimadzu FT-IR 8400S spectrophotometer has been used for functional group analysis at the micro analytical unit, Cairo University, Cairo, Egypt. Elemental analyses were performed at the Regional Centre for Microbiology and Biotechnology, Al-Azhar University, Cairo, Egypt.

#### 4.1.2. General methodology for preparation of the target compounds 1-12

In DMF (10 ml), a mixture of potassium salts **IIIa-c** (0.001 mol) and the convenient 4-(2-chloroacetamido)-*N*-(substituted) phenyl benzamide **VIIa-d** (0.001 mol), and KI (0.001 mol) was heated at 60 °C for 6 h. After completion of the reaction, the mixture was poured on crushed ice. The formed precipitates were filtered, dried, and recrystallized from methanol to afford the corresponding final target compounds **1–12**.

#### 4.1.3. General methodology for preparation of the target compounds 13-15

In DMF (10 ml), a mixture of potassium salts **IIIa-c** (0.001 mol) and *N*-(4-(2-benzoyl-hydrazine-1-carbonyl)phenyl)-2-chloroacetamide **XI** (0.001 mol), and KI (0.001 mol) was heated at 60 °C for 6 h. After completion of the reaction, the mixture was poured on crushed ice. The formed precipitates were filtered, dried, and recrystallized from methanol to afford the corresponding final target compounds **13–15**.

Full characterisation (^1^HNMR, ^13^CNMR, IR, Mass spectrum and elemental analysis) data for novel compounds **1–15** have been presented in the Supplementary Materials.

### 4.2. Biological evaluation

All the procedures of the experiment utilised for biological evaluation in this article were performed as previously described; cytotoxicity^63^^,^^65^, VEGFR-2 inhibitory activity^14^, cell cycle analysis^66^, Annexin V-FITC/PI apoptosis assay^67^, and anti-apoptotic markers (Bcl-2, and Bcl-xL)^68^^,^^69^. All procedures were mentioned in detail in the Supplementary Materials.

### 4.3. Molecular docking

Virtual Molecular Docking studies were carried out using Molecular Operating Environment (MOE®) version 2015.10. The RCSB: Protein Data Bank was utilised to retrieve the crystal structure of Vascular Endothelial Growth Factor Receptor (VEGFR) co-crystallized with sorafenib (PDB ID: 4ASD) ^70^. The downloaded protein was used for the docking study as a receptor and sorafenib was used as a reference drug.

## Supplementary Material

Supplemental Material
