# Supplementary material for: Design, synthesis, molecular modeling and biological evaluation of novel Benzoxazole-Benzamide conjugates via a 2-Thioacetamido linker as potential anti-proliferative agents, VEGFR-2 inhibitors and apoptotic inducers
Source: J Enzyme Inhib Med Chem. 2022 May 30;37(1):1587–99. doi: 10.1080/14756366.2022.2081844 (PMC9176662; doi:10.1080/14756366.2022.2081844)
Supplement: Supplemental Material [file IENZ_A_2081844_SM8898.pdf]

Supplementary Materials

Design, Synthesis, Molecular Modeling and Biological Evaluation of Novel Benzoxazole-Benzamide Conjugates *via* a 2-Thioacetamido Linker as Potential Anti-proliferative Agents, VEGFR-2 Inhibitors and Apoptotic Inducers.

Ibrahim H. Eissa[1], Radwan El-Haggar[2,3,*], Mohammed A. Dahab[1], Marwa F. Ahmed[2,4], Hazem A. Mahdy[1], Reem I. Alsantali[4], Alaa Elwan[1,*], Nicolas Masurier[3,*], Samar S. Fatahala[5]

[1]*Pharmaceutical Medicinal Chemistry & Drug Design Department, Faculty of Pharmacy (Boys), Al-Azhar University, Cairo 11884, Egypt.*

[2]*Pharmaceutical Chemistry Department, Faculty of Pharmacy, Helwan University, 11795 Cairo, Egypt.*

[3]*Institut des Biomolécules Max Mousseron (IBMM), UMR 5247, CNRS, Université de Montpellier, ENSCM, Montpellier, France.*

[4]*Department of Pharmaceutics, College of Pharmacy, Taif University, P.O. Box 11099, Taif 21944, Saudi Arabia.*

[5]*Pharmaceutical Organic Chemistry Department, Faculty of Pharmacy, Helwan University, 11795 Cairo, Egypt.*

* Corresponding authors.

e-mail addresses: radwan_elhaggar@pharm.helwan.edu.eg (Radwan El-Haggar), alaaelwan34@azhar.edu.eg (Alaa Elwan), nicolas.masurier@umontpellier.fr (Nicolas Masurier)

Supplementary Materials

Characterization and Spectral analysis of compounds **1-15** 2

Elemental Analysis 62

Biological evaluation 63

Molecular docking 64

1. Characterization and Spectral analysis of compounds 1-15

1.1. 4-(2-(benzo[d]oxazol-2-ylthio)acetamido)-N-cyclohexylbenzamide (1):

Buff powder (yield 70%); m.p. 266-268 °C; IR ($\upsilon_{max.}$/cm^{-1}) 3300 (N-H), 2928 (C-H aliphatic), 1666 and 1629 (2*C=O); ^{1}H NMR (400 MHz, DMSO-d_6) δ 10.61 (s, 1H, exchangeable with D_2O, (*$NHCOCH_2S$-)), 8.06 (d, *J* = 7.91 Hz, 1H, exchangeable with D_2O), 7.80 (d, *J* = 8.79 Hz, 2H), 7.57 - 7.66 (m, 4H), 7.28 - 7.35 (m, 2H), 4.39 (s, 2H), 3.66 - 3.76 (m, 1H), 1.78 (br. s., 2H), 1.69 - 1.75 (m, 2H), 1.58 (d, *J* = 11.43 Hz, 1H), 1.27 (t, *J* = 9.45 Hz, 4H), 1.04 - 1.16 (m, 1H). ^{13}C NMR (101 MHz, DMSO-d_6) δ 165.3, 164.6, 163.8, 151.3, 141.2, 141.0, 129.8, 128.2, 124.7, 124.3, 118.2, 118.2, 110.2, 48.2, 36.7, 32.4, 25.3, 24.9. MS (m/z (R.I. %)): [M]$^{+}$ 409 (19.5), 249 (33.9), 192 (100), 161 (51.5), 120 (88.7); Anal. Calcd. For $C_{22}H_{23}N_3O_3S$ (409.50); % C, 64.53; H, 5.66; N, 10.26, Found: % C, 64.31; H, 5.89; N, 10.45.

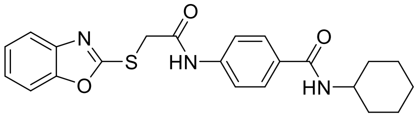

IR

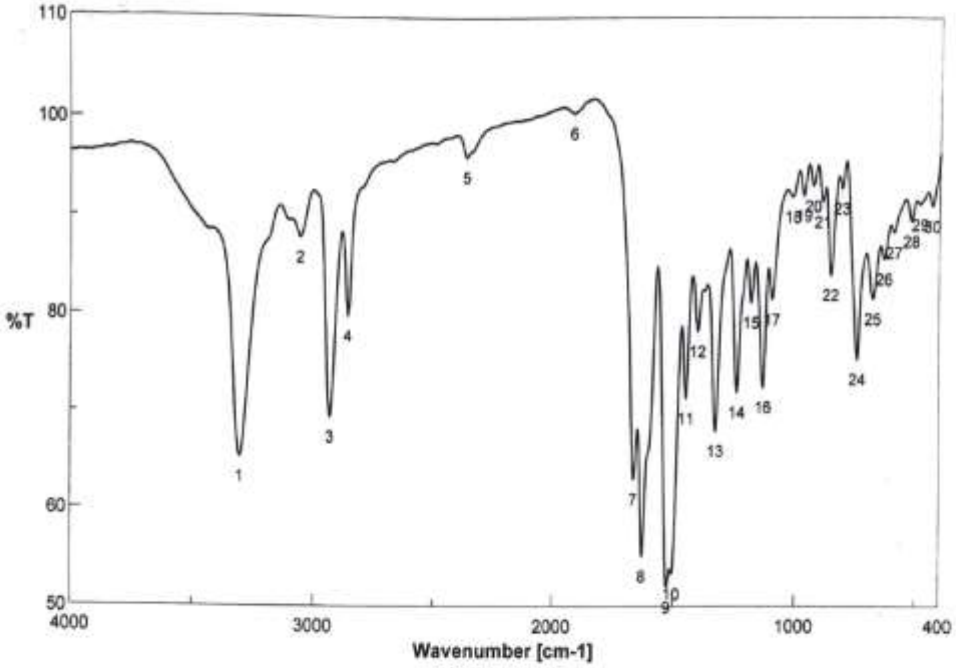

Accumulation	Auto (32)
Resolution	4 cm-1
Zero Filling	ON
Apodization	Cosine
Gain	Auto (2)
Scanning Speed	Auto (2 mm/sec)
Date/Time	5/9/2021 0:10PM
Update	5/9/2021 0:11PM
Operator	IR
File Name	Memory#89
Sample Name	PBA-8
Comment	

Mass

Cairo University
Micro Analytical Center

DI Analysis
Shimadzu Qp-2010 Plus

Sample Information

Analyzed by	: Dr. Mai Younis
Analyzed	: 01/01/2007 06:09:57 ص
Sample Name	: 8
Sample ID	:
Customer Name	: Dr.Radwan Saeed - Pharmacy - Helwan
Data File	: C:\GCMSsolution\Data\Project1\8.QGD
Org Data File	: C:\GCMSsolution\Data\Project1\8.QGD
Method File	: C:\GCMSsolution\Data\Project1\High Temperature Op
Org Method File	: C:\GCMSsolution\Data\Project1\High Temperature Op
Report File	:
Tuning File	: C:\GCMSsolution\System\Tune1_default.qgt
$EndIf$Modified by	: Dr. Mai Younis
Modified	: 01/01/2007 06:12:48 ص

Method

—— Analytical Line 1 ——

IonSourceTemp	:250.00 °C
[MS Table]	
--Group 1 - Event 1--	
Start Time	:0.00min
End Time	:10.00min
ACQ Mode	:Scan
Event Time	:0.50sec
Scan Speed	:1000
Start m/z	:50.00
End m/z	:500.00
Electron Voltage	: 70 eV
Ionization Mode	: EI

C:\GCMSsolution\Data\Project1\8.QGD

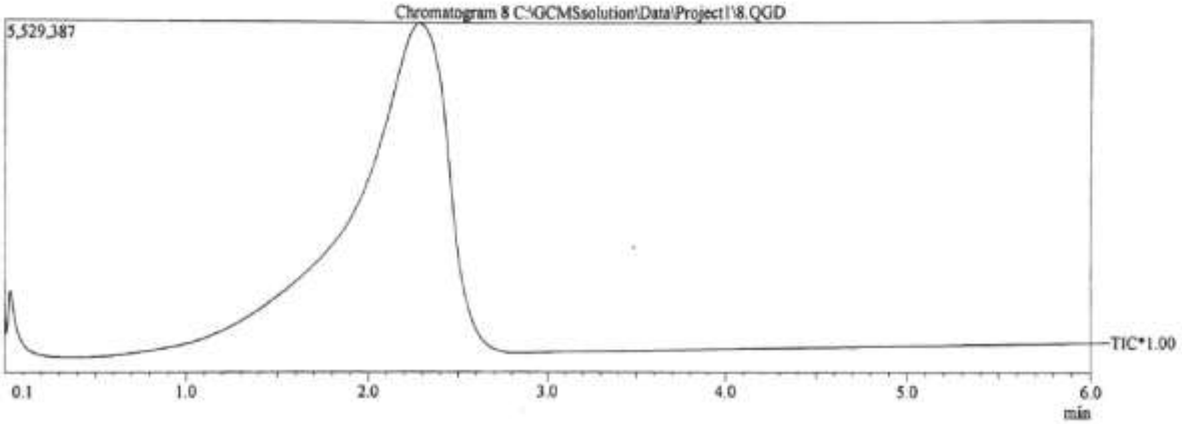

Spectrum

Line#:1 R.Time:2.3(Scan#:278)
MassPeaks:331
RawMode:Single 2.3(278) BasePeak:192(431947)
BG Mode:None Group 1 - Event 1

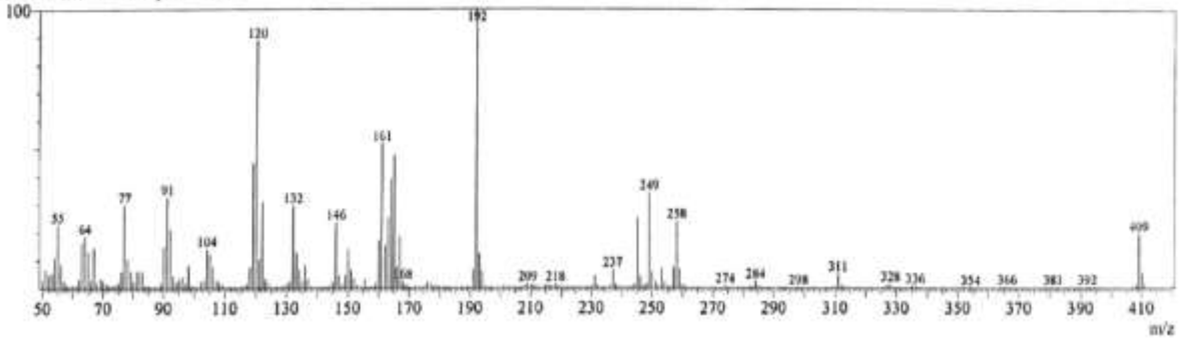

1HNMR

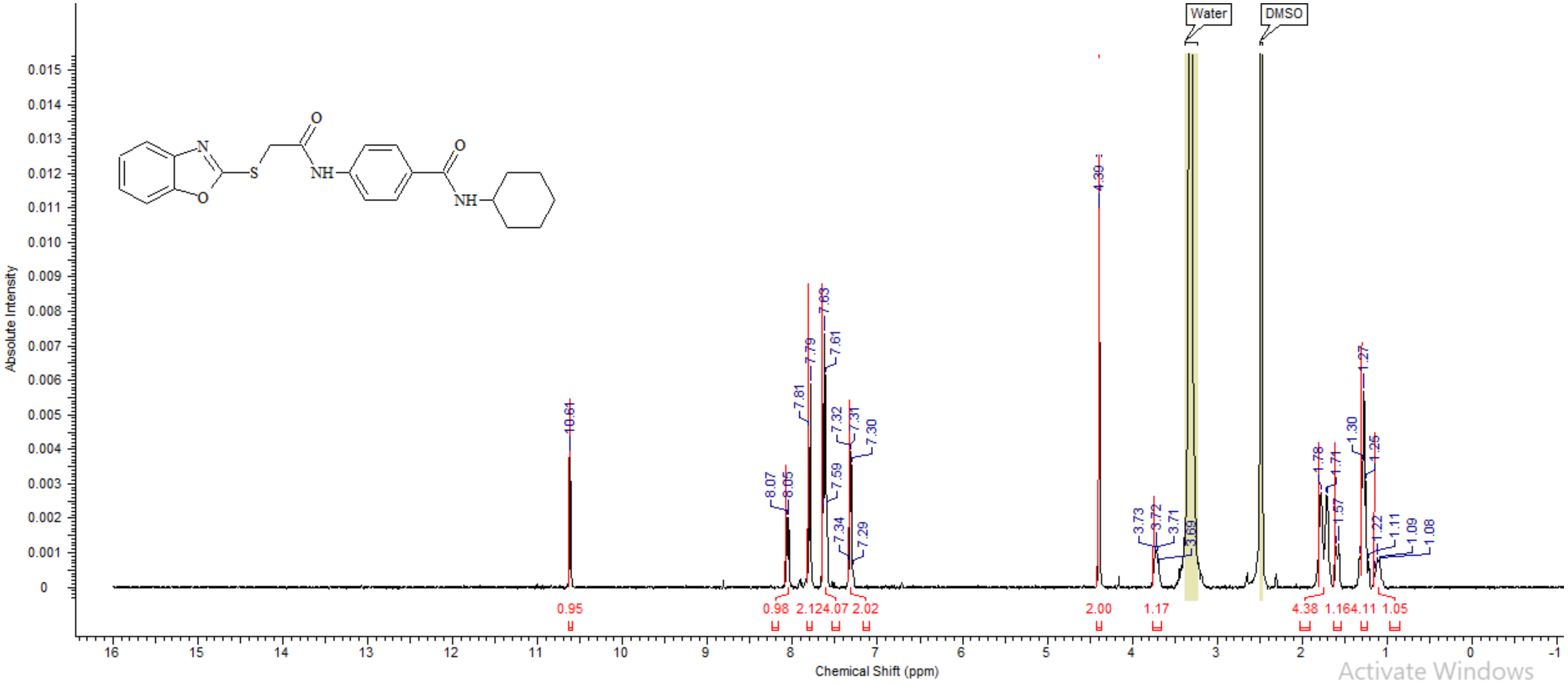

13CNMR

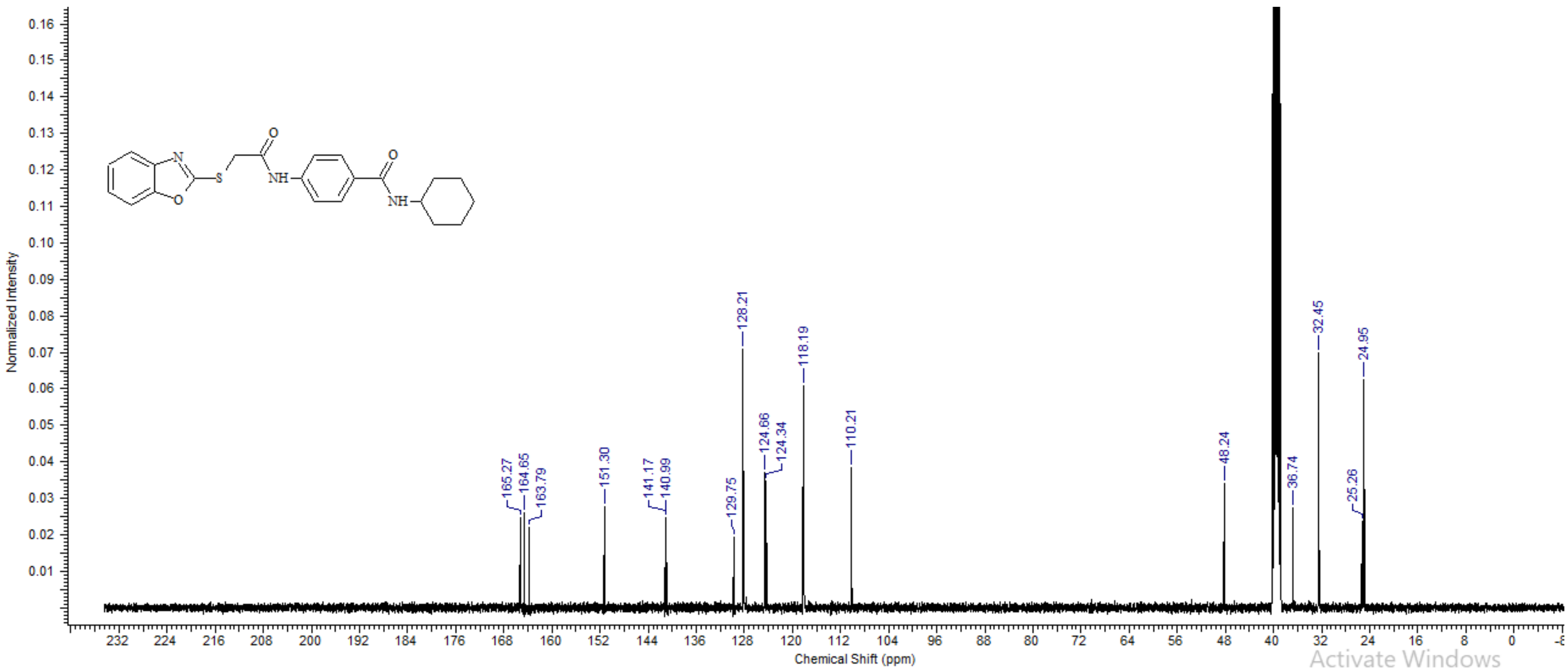

Normalized Intensity
Chemical Shift (ppm)
165.27
164.65
163.79
151.30
141.17
140.99
129.75
128.21
124.66
124.34
118.19
110.21
48.24
36.74
32.45
25.26
24.95
N
O
S
NH
NH

1.2. 4-(2-(benzo[d]oxazol-2-ylthio)acetamido)-N-phenylbenzamide (2):

Off-white powder (yield 80%); m.p. 250-252 ºC; IR ($\upsilon_{max.}$/cm^{-1}) 3298 (N-H), 1673 and 1642 (2*C=O); ^{1}H NMR (400 MHz, DMSO-d_6) δ 10.71 (s, 1H, exchangeable with D_2O, (*NHCOCH$_2$S-)), 10.11 (s, 1H, exchangeable with D_2O), 7.94 (d, *J* = 8.35 Hz, 2H), 7.71 (d, *J* = 8.79 Hz, 2H), 7.74 (d, *J* = 7.91 Hz, 2H), 7.58 - 7.67 (m, 2H), 7.29 - 7.35 (m, 4H), 7.03 - 7.10 (m, 1H), 4.41 (s, 2H). ^{13}C NMR (101 MHz, DMSO-d_6) δ 165.5, 164.8, 163.8, 151.3, 141.6, 141.2, 139.2, 129.7, 128.7, 128.6, 124.7, 124.4, 123.5, 120.4, 118.4, 118.3, 110.3, 36.8. MS (m/z (R.I. %)): [M]$^+$ 403 (16.3), 311 (35.0), 192 (34.0), 120 (100); Anal. Calcd. For $C_{22}H_{17}N_3O_3S$ (403.45); % C, 65.49; H, 4.25; N, 10.42, Found: % C, 65.31; H, 5.89; N, 10.44.

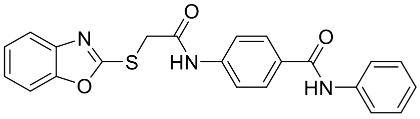

IR

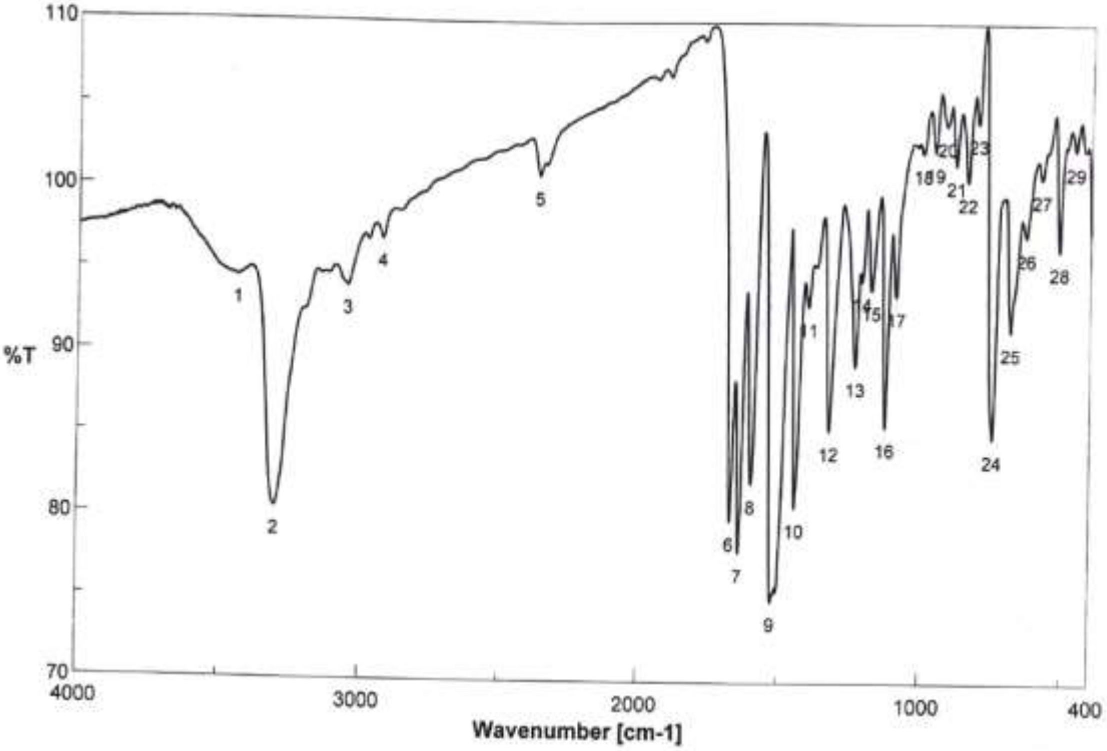

Accumulation	Auto (29)
Resolution	4 cm-1
Zero Filling	ON
Apodization	Cosine
Gain	Auto (2)
Scanning Speed	Auto (2 mm/sec)
Date/Time	5/9/2021 11:55AM
Update	5/9/2021 11:55AM
Operator	IR
File Name	Memory#58
Sample Name	PBA -1
Comment	

Mass

Cairo University
Micro Analytical Center

DI Analysis
Shimadzu Qp-2010 Plus

Sample Information

Analyzed by	: Dr. Mai Younis
Analyzed	: 01/01/2007 05:37:47 ص
Sample Name	: 3
Sample ID	:
Customer Name	: Dr.Radwan Saeed - Pharmacy - Helwan
Data File	: C:\GCMSsolution\Data\Project1\3.QGD
Org Data File	: C:\GCMSsolution\Data\Project1\3.QGD
Method File	: C:\GCMSsolution\Data\Project1\High Temperature Op
Org Method File	: C:\GCMSsolution\Data\Project1\High Temperature Op
Report File	:
Tuning File	: C:\GCMSsolution\System\Tune1_default.qgt
$EndIf$Modified by	: Dr. Mai Younis
Modified	: 01/01/2007 05:41:45 ص

Method

------ Analytical Line 1 ------

IonSourceTemp	:250.00 °C
[MS Table]	
--Group 1 - Event 1--	
Start Time	:0.00min
End Time	:10.00min
ACQ Mode	:Scan
Event Time	:0.50sec
Scan Speed	:1000
Start m/z	:50.00
End m/z	:500.00
Electron Voltage	: 70 eV
Ionization Mode	: EI

C:\GCMSsolution\Data\Project1\3.QGD

Chromatogram 3 C:\GCMSsolution\Data\Project1\3.QGD

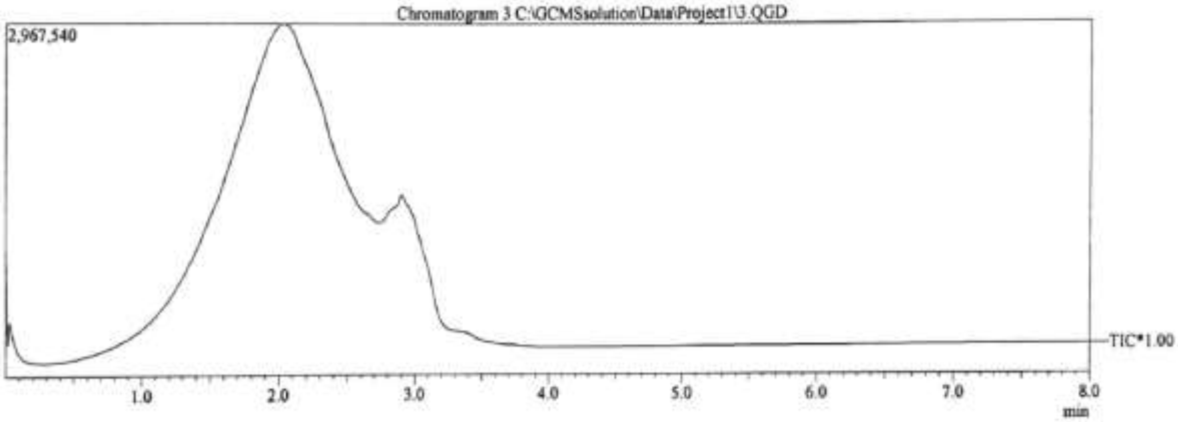

Spectrum

Line#:1 R.Time:2.0(Scan#:243)
MassPeaks:271
RawMode:Single 2.0(243) BasePeak:120(489517)
BG Mode:None Group 1 - Event 1

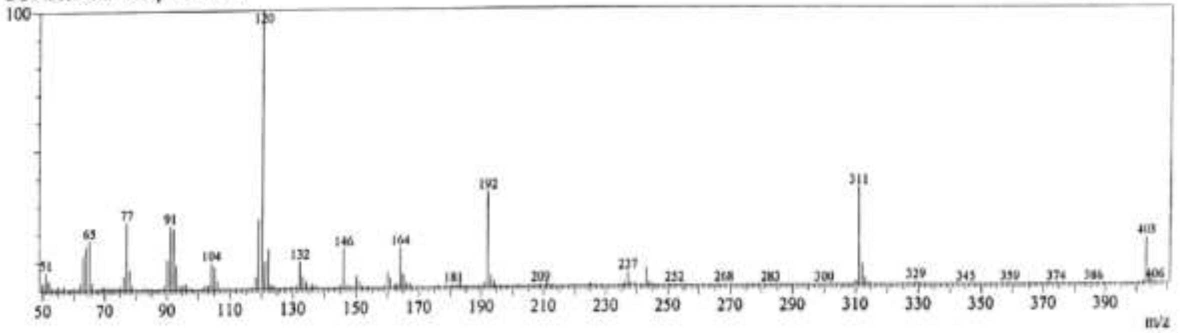

1HNMR

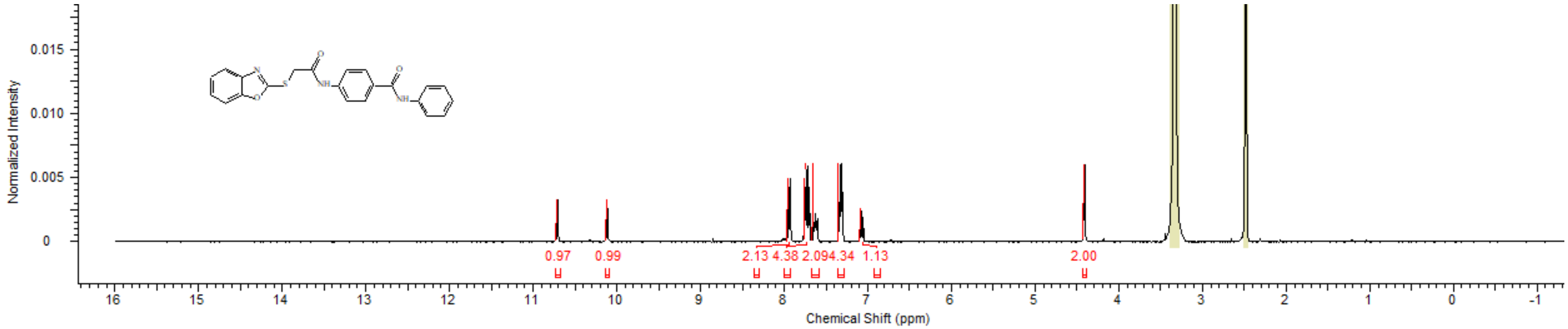

Normalized Intensity
0.97
0.99
2.13 4.38 2.094.34 1.13
2.00
Chemical Shift (ppm)

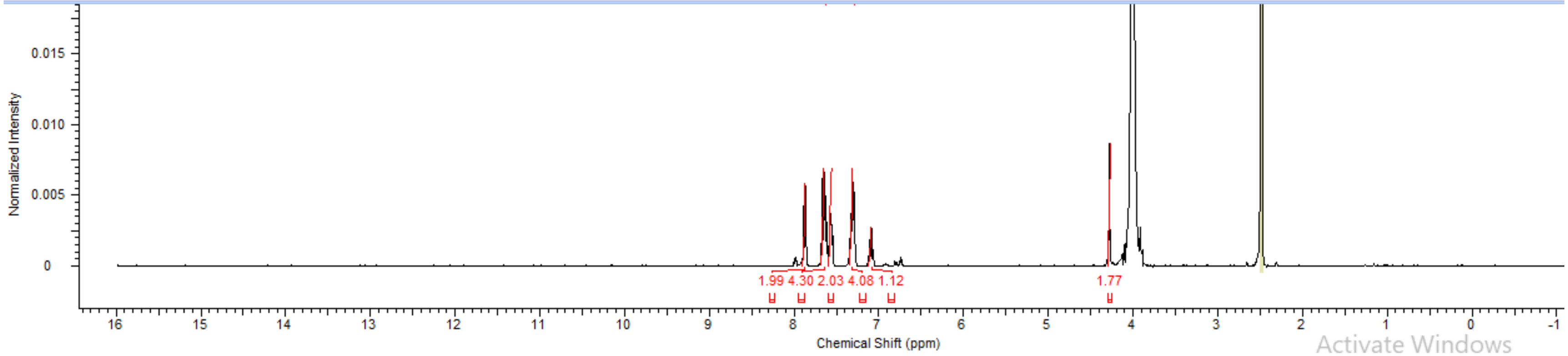

Normalized Intensity
1.99 4.30 2.03 4.08 1.12
1.77
Chemical Shift (ppm)

13CNMR

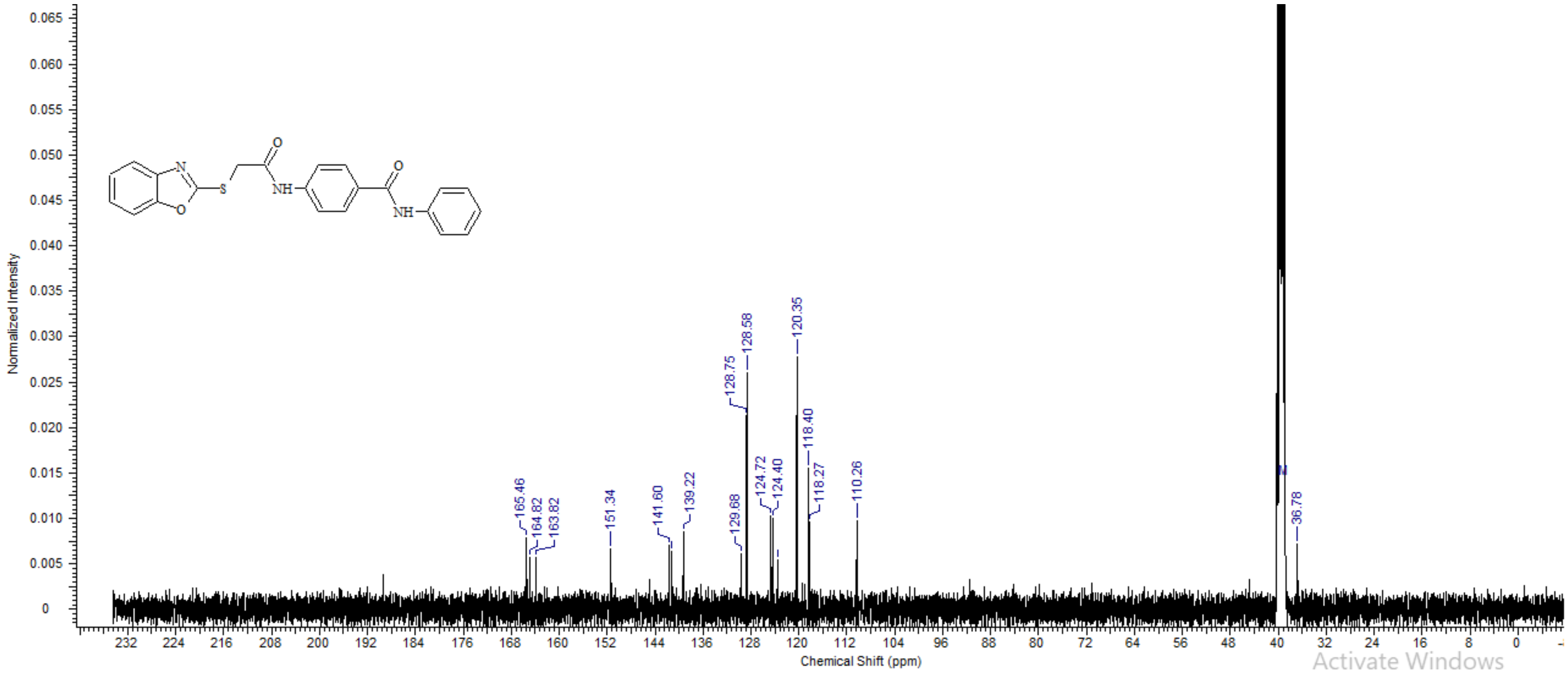

Normalized Intensity
Chemical Shift (ppm)
165.46
164.82
163.82
151.34
141.60
139.22
129.68
128.75
128.58
124.72
124.40
120.35
118.40
118.27
110.26
36.78
N
O
S
NH
Activate Windows

1.3. 4-(2-(benzo[d]oxazol-2-ylthio)acetamido)-N-(4-chlorophenyl)benzamide (3):

Yellow powder (yield 60%); m.p. 238-240 °C; IR ($\upsilon_{max.}$/cm^{-1}) 3303 (N-H), 1656 and 1598 (2*C=O); ^{1}H NMR (400 MHz, DMSO-d_6) δ 10.72 (s, 1H, exchangeable with D_2O, (*NHCOCH_2S-)), 10.24 (s, 1H, exchangeable with D_2O), 7.91 - 7.96 (m, *J* = 8.35 Hz, 2H), 7.79 (d, *J* = 8.79 Hz, 2H), 7.70 - 7.74 (m, *J* = 8.79 Hz, 2H), 7.59 - 7.66 (m, 2H), 7.38 (d, *J* = 8.79 Hz, 2H), 7.30 - 7.34 (m, 2H), 4.41 (s, 2H). ^{13}C NMR (101 MHz, DMSO-d_6) δ 165.5, 164.9, 163.8, 151.3, 141.7, 141.2, 138.2, 129.3, 128.8, 128.5, 127.1, 124.7, 124.4, 121.8, 118.4, 118.2, 110.2, 36.8. MS (m/z (R.I. %)): [M]$^+$ 437 (18.3), [M+2]$^+$ 439 (7.3), 311 (63.1), 192 (47.2), 120 (100); Anal. Calcd. For $C_{22}H_{16}ClN_3O_3S$ (437.89); % C, 60.24; H, 3.68; N, 9.60, Found: % C, 60.47; H, 3.80; N, 9.86.

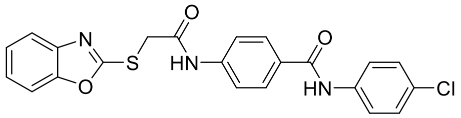

IR

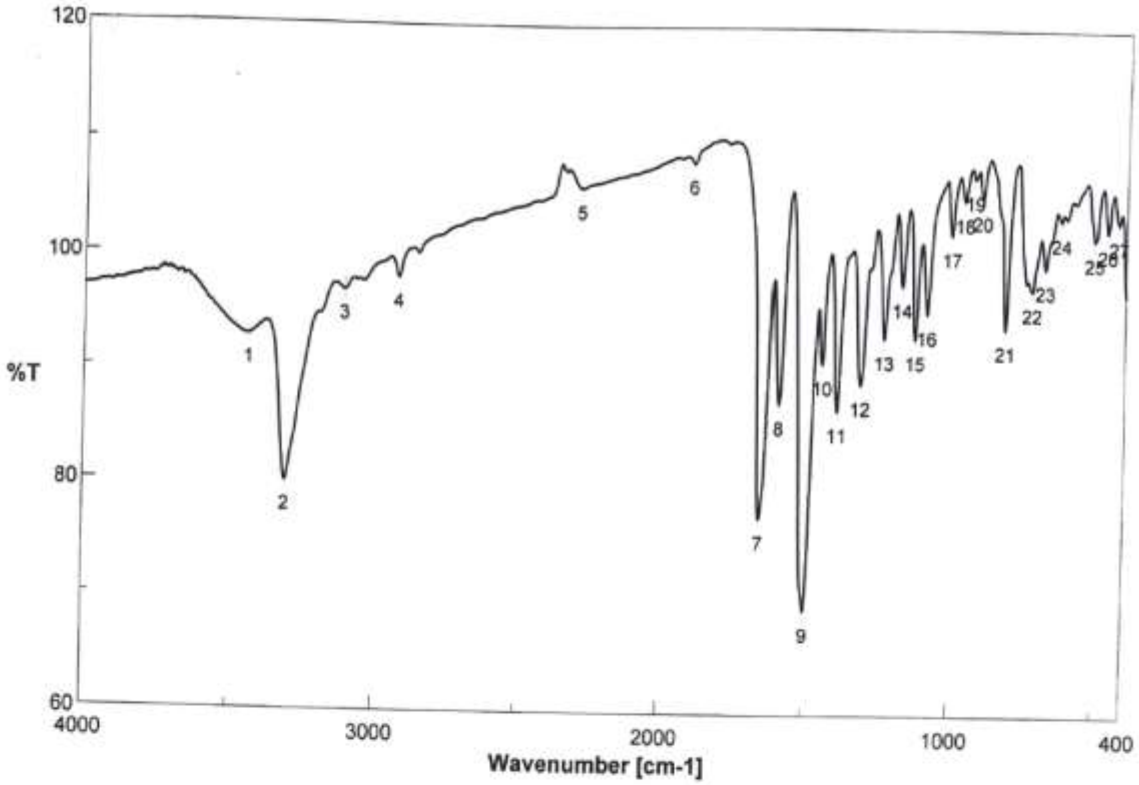

Accumulation	Auto (32)
Resolution	4 cm-1
Zero Filling	ON
Apodization	Cosine
Gain	Auto (2)
Scanning Speed	Auto (2 mm/sec)
Date/Time	5/9/2021 0:08PM
Update	5/9/2021 0:09PM
Operator	IR
File Name	Memory#84
Sample Name	PBA-3
Comment	

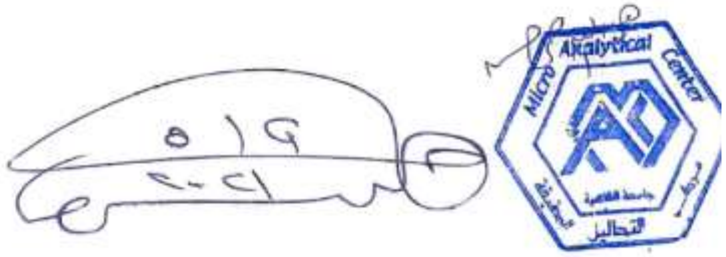

Mass

Cairo University
Micro Analytical Center

DI Analysis
Shimadzu Qp-2010 Plus

Sample Information

Analyzed by	: Dr. Mai Younis
Analyzed	: 01/01/2007 05:51:03 ص
Sample Name	: 5
Sample ID	:
Customer Name	: Dr.Radwan Saeed - Pharmacy - Helwan
Data File	: C:\GCMSsolution\Data\Project1\5.QGD
Org Data File	: C:\GCMSsolution\Data\Project1\5.QGD
Method File	: C:\GCMSsolution\Data\Project1\High Temperature Op
Org Method File	: C:\GCMSsolution\Data\Project1\High Temperature Op
Report File	:
Tuning File	: C:\GCMSsolution\System\Tune1_default.qgt
$EndIf$Modified by	: Dr. Mai Younis
Modified	: 01/01/2007 05:55:39 ص

Method

===== Analytical Line 1 =====

IonSourceTemp	:250.00 °C
[MS Table]	
--Group 1 - Event 1--	
Start Time	:0.00min
End Time	:10.00min
ACQ Mode	:Scan
Event Time	:0.50sec
Scan Speed	:1000
Start m/z	:50.00
End m/z	:500.00
Electron Voltage	: 70 eV
Ionization Mode	: EI

C:\GCMSsolution\Data\Project1\5.QGD

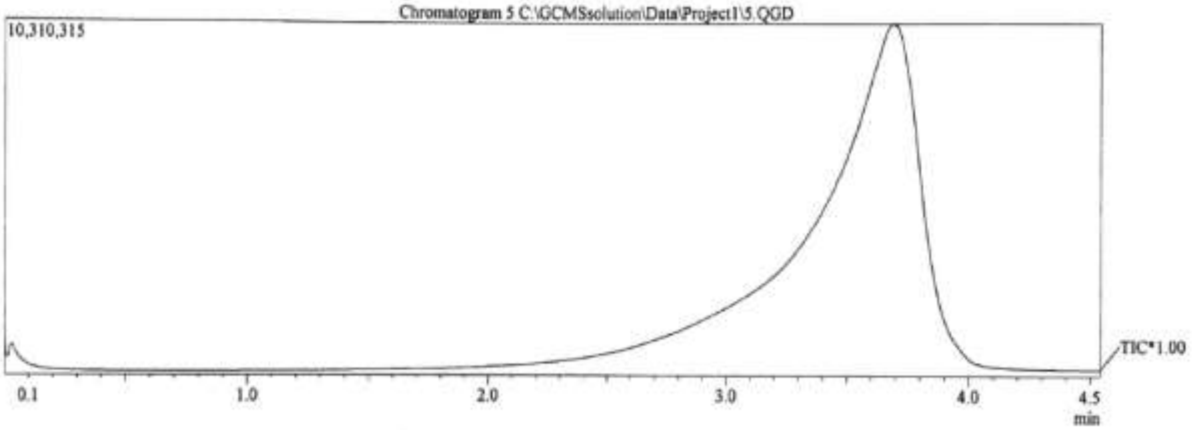

Spectrum

Line#:1 R.Time:3.7(Scan#:445)
MassPeaks:388
RawMode:Single 3.7(445) BasePeak:120(1366202)
BG Mode:None Group 1 - Event 1

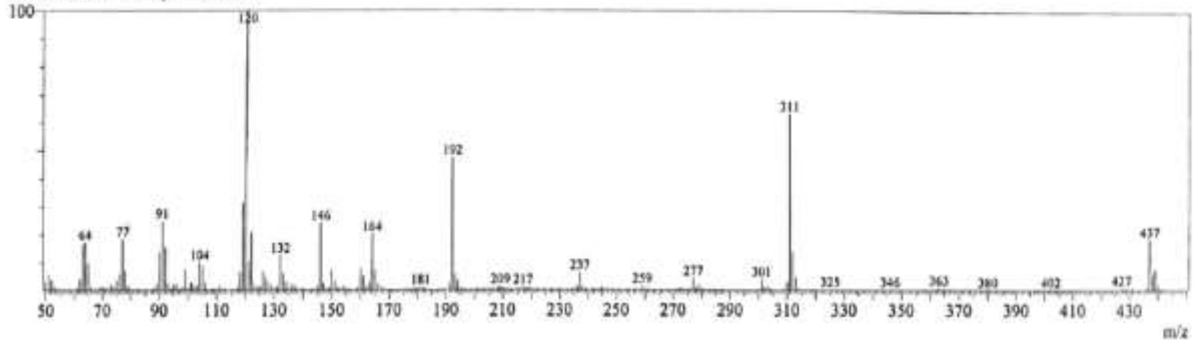

1HNMR

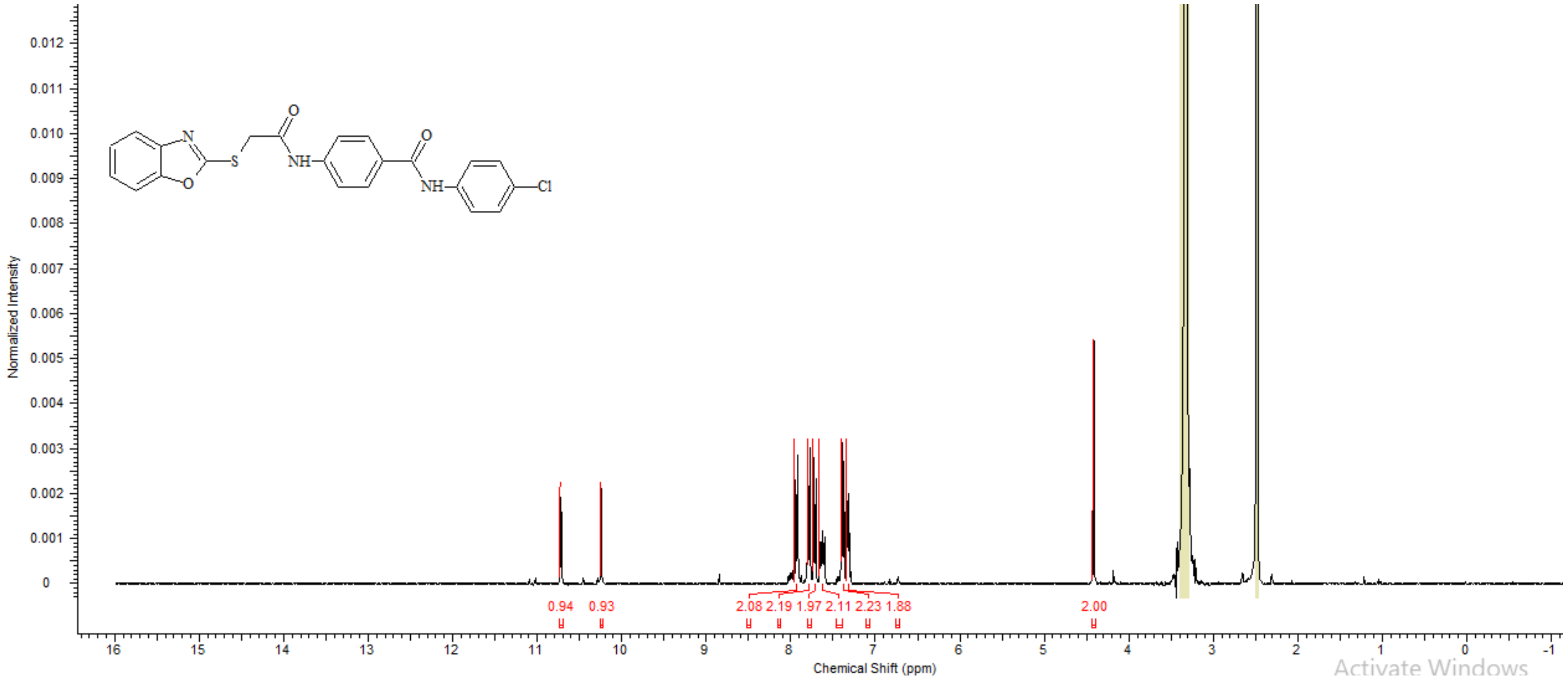

Normalized Intensity
0.012
0.011
0.010
0.009
0.008
0.007
0.006
0.005
0.004
0.003
0.002
0.001
0
0.94
0.93
2.08
2.19
1.97
2.11
2.23
1.88
2.00
16
15
14
13
12
11
10
9
8
7
6
5
4
3
2
1
0
-1
Chemical Shift (ppm)
N
O
S
O
NH
O
NH
Cl
Activate Windows

13CNMR

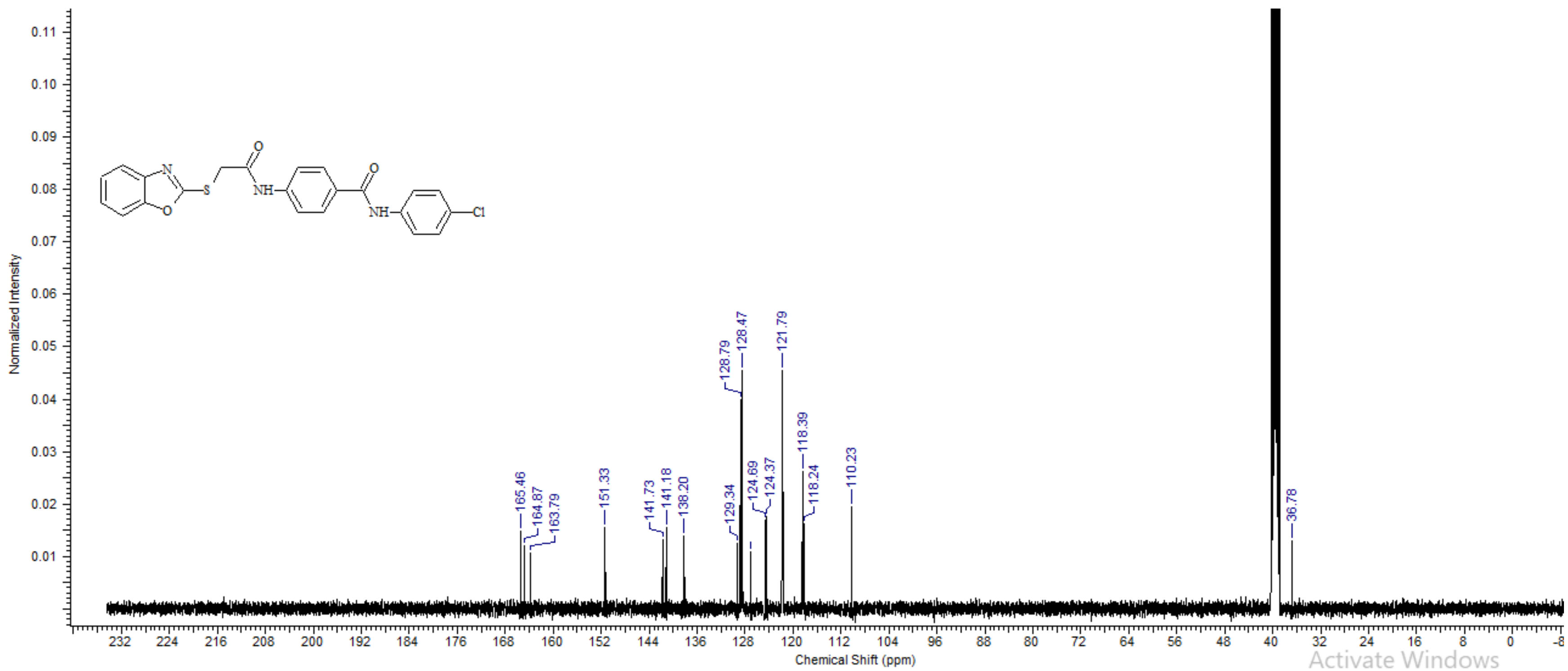

Activate Windows

1.4. 4-(2-(benzo[d]oxazol-2-ylthio)acetamido)-N-(4-methoxyphenyl)benzamide (4):

Yellow powder (yield 75%); m.p. 260-262 °C; IR ($\upsilon_{max.}$/cm^{-1}) 3308 (N-H), 1660 and 1640 (2*C=O); ^{1}H NMR (400 MHz, DMSO-d_6) δ 10.69 (s, 1H, exchangeable with D_2O, (*$NHCOCH_2S$-)), 10.00 (s, 1H, exchangeable with D_2O), 7.93 (d, *J* = 8.79 Hz, 2H), 7.70 (d, *J* = 8.79 Hz, 2H), 7.60 - 7.67 (m, 4H), 7.28 - 7.36 (m, 2H), 6.90 (d, *J* = 9.23 Hz, 2H), 4.42 (s, 2H), 3.72 (s, 3H). ^{13}C NMR (101 MHz, DMSO-d_6) δ 165.4, 164.4, 163.8, 155.4, 151.3, 141.4, 141.2, 132.3, 129.8, 128.6, 124.7, 124.4, 121.9, 118.4, 118.2, 113.7, 110.2, 55.1, 36.8. MS (m/z (R.I. %)): $[M]^+$ 433 (67.7), 311 (41.4), 192 (46.6), 120 (100); Anal. Calcd. For $C_{23}H_{19}N_3O_4S$ (433.48); % C, 63.73; H, 4.42; N, 9.69, Found: % C, 63.90; H, 4.63; N, 9.95.

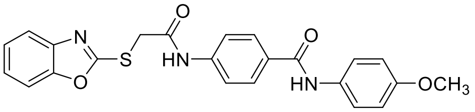

IR

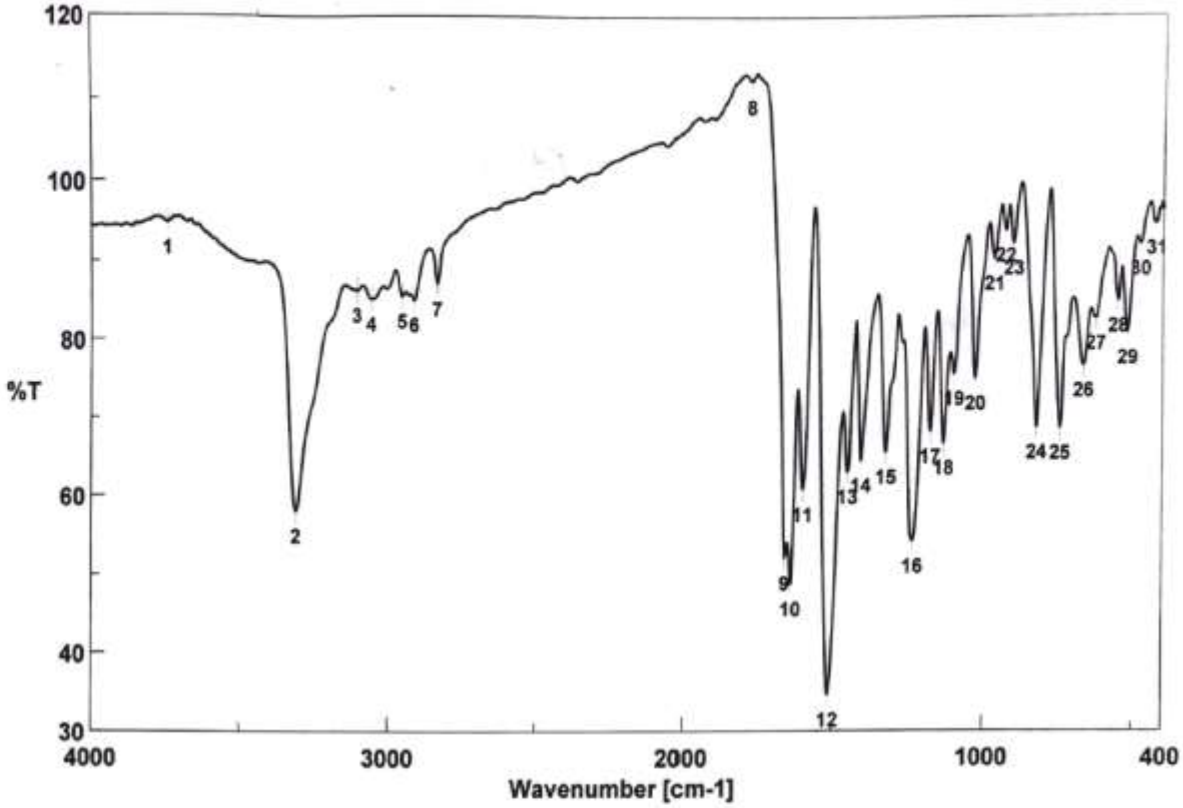

Accumulation	16
Resolution	4 cm-1
Zero Filling	ON
Apodization	Cosine
Gain	Auto (2)
Scanning Speed	Auto (2 mm/sec)
Date/Time	8/22/2021 2:07PM
Update	8/22/2021 2:07PM
Operator	IR
File Name	Memory#125
Sample Name	PBA -19
Comment	

Mass

Cairo University
Micro Analytical Center

DI Analysis
Shimadzu Qp-2010 Plus

Sample Information

Analyzed by	: Dr. Mai Younis
Analyzed	: 01/01/2007 10:46:47 م
Sample Name	: 17
Sample ID	:
Customer Name	: Dr.Radwan Saeed - Pharmacy - Helwan
Data File	: C:\GCMSsolution\Data\Project1\17.QGD
Org Data File	: C:\GCMSsolution\Data\Project1\17.QGD
Method File	: C:\GCMSsolution\Data\Project1\High Temperature Op
Org Method File	: C:\GCMSsolution\Data\Project1\High Temperature Op
Report File	:
Tuning File	: C:\GCMSsolution\System\Tune1_default.qgt
$EndIf$Modified by	: Dr. Mai Younis
Modified	: 01/01/2007 10:52:11 م

Method

------ Analytical Line 1 ------

IonSourceTemp	:250.00 °C
[MS Table]	
--Group 1 - Event 1--	
Start Time	:0.00min
End Time	:10.00min
ACQ Mode	:Scan
Event Time	:0.50sec
Scan Speed	:1250
Start m/z	:50.00
End m/z	:600.00
Electron Voltage	: 70 eV
Ionization Mode	: EI

Dr. Mai Y

Analytical

C:\GCMSsolution\Data\Project1\17.QGD

Chromatogram 17 C:\GCMSsolution\Data\Project1\17.QGD

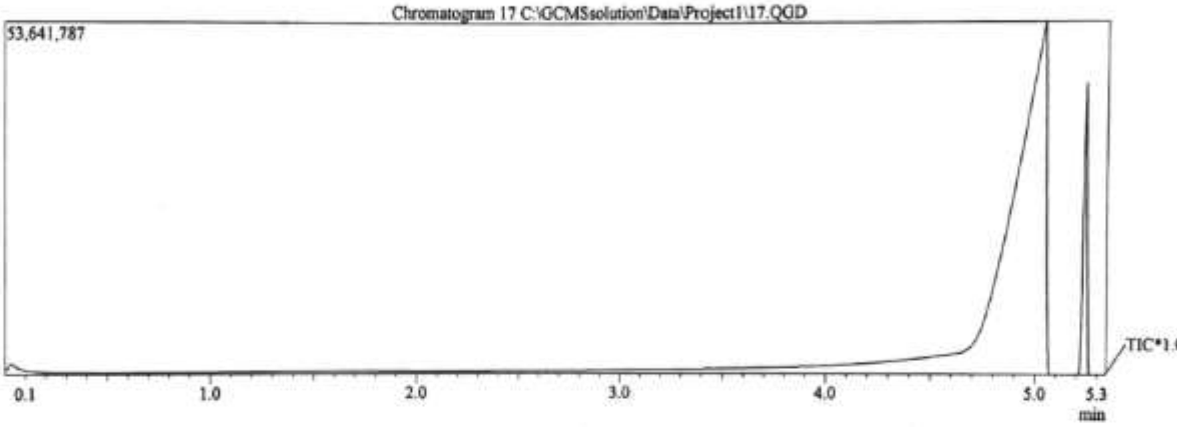

Spectrum

Line#:1 R.Time:5.0(Scan#:595)
MassPeaks:395
RawMode:Single 5.0(595) BasePeak:120(4585821)
BG Mode:None Group 1 - Event 1

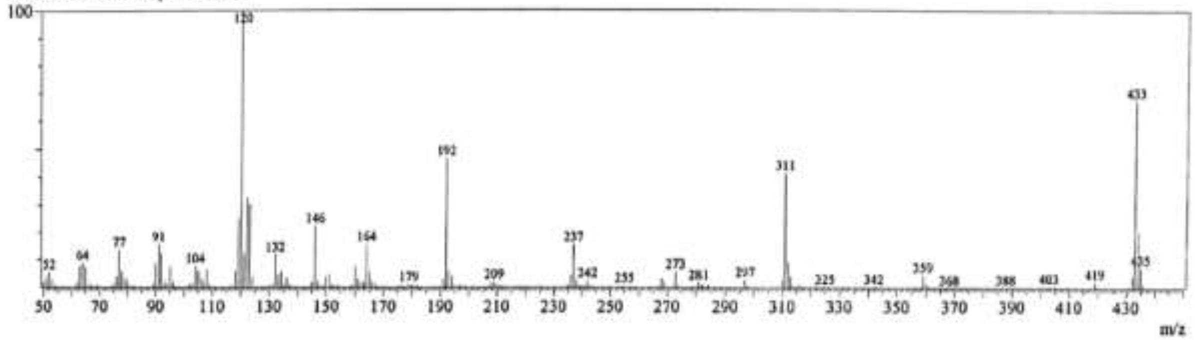

1HNMR

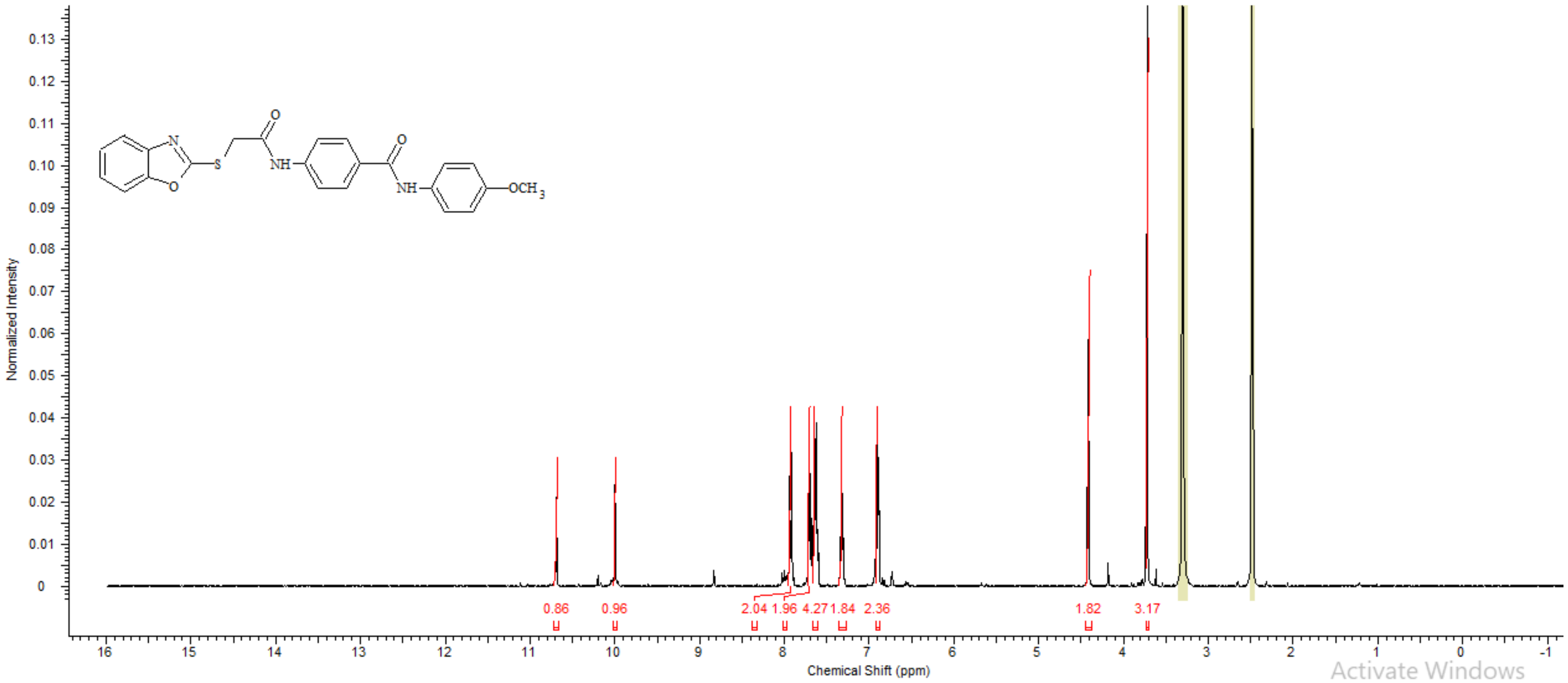

Normalized Intensity
Chemical Shift (ppm)
0.86
0.96
2.04
1.96
4.27
1.84
2.36
1.82
3.17
NH
NH
OCH 3

13CNMR

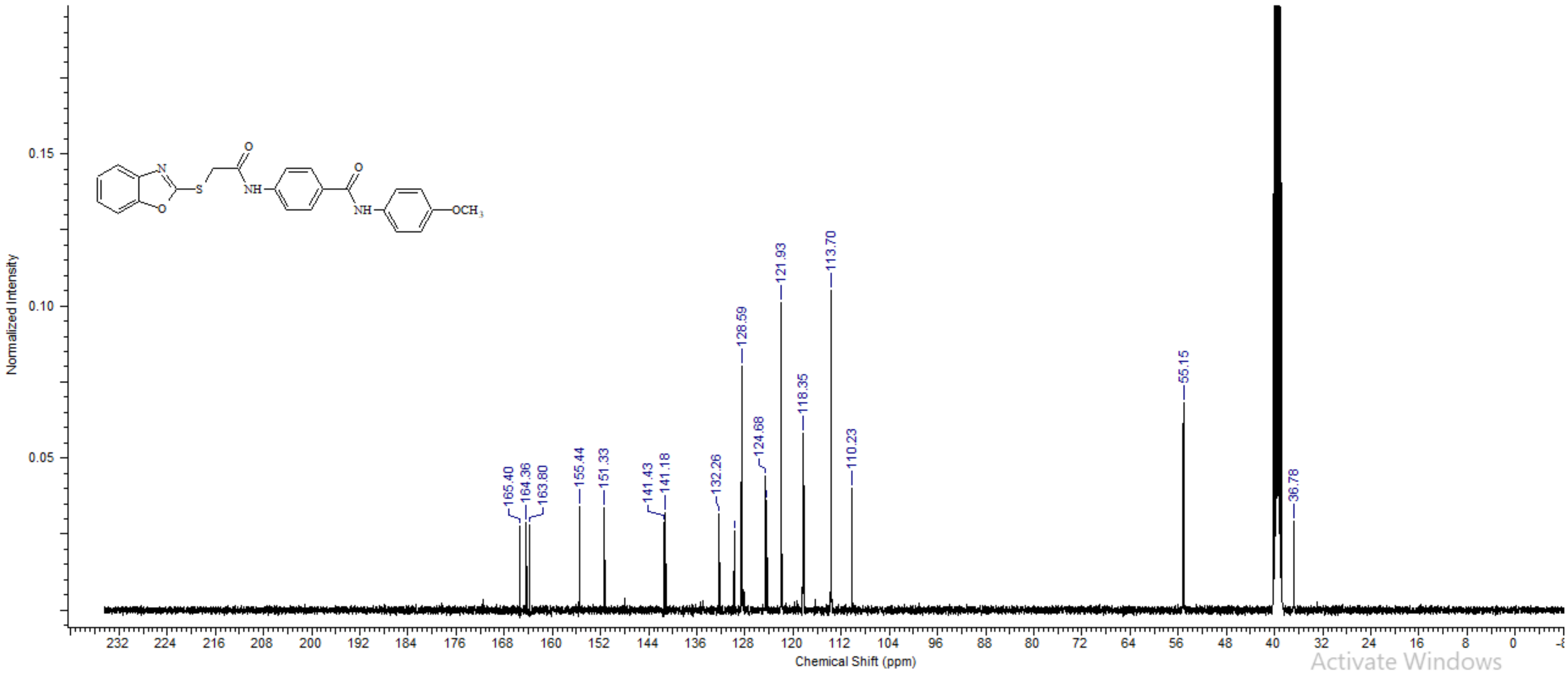

N
S
O
NH
O
NH
OCH3
0.15
0.10
0.05
Normalized Intensity
165.40
164.36
163.80
155.44
151.33
141.43
141.18
132.26
128.59
124.68
121.93
118.35
113.70
110.23
55.15
36.78
232
224
216
208
200
192
184
176
168
160
152
144
136
128
120
112
104
96
88
80
72
64
56
48
40
32
24
16
8
0
Chemical Shift (ppm)
Activate Windows

1.5. 4-(2-(5-methylbenzo[d]oxazol-2-ylthio)acetamido)-N-cyclohexylbenzamide (5):

Off-white powder (yield 72%); m.p. 256-258ºC; IR ($\upsilon_{max.}$/cm^{-1}) 3316 and 3273 (N-H), 2927 (C-H aliphatic), 1673 and 1628 (2*C=O); ^{1}H NMR (400 MHz, DMSO-d_6) δ 10.61 (s, 1H, exchangeable with D_2O, (*$NHCOCH_2S$-)), 8.06 (d, *J* = 7.91 Hz, 1H, exchangeable with D_2O), 7.77 - 7.83 (m, *J* = 8.79 Hz, 2H), 7.59 - 7.64 (m, *J* = 8.79 Hz, 2H), 7.49 (d, *J* = 7.91 Hz, 1H), 7.40 (s, 1H), 7.11 (d, *J* = 8.35 Hz, 1H), 4.37 (s, 2H), 3.72 (br. s., 1H), 2.37 (s, 3H), 1.78 (br. s., 2H), 1.68 - 1.75 (m, 2H), 1.58 (d, *J* = 12.30 Hz, 1H), 1.21 - 1.34 (m, 4H), 1.03 - 1.17 (m, 1H). ^{13}C NMR (101 MHz, DMSO-d_6) δ 165.3, 164.6, 163.7, 149.6, 141.4, 141.0, 134.1, 129.7, 128.2, 125.1, 118.2, 118.2, 109.6, 48.2, 36.7, 32.4, 25.3, 24.9, 20.9. MS (m/z (R.I. %)): $[M]^+$ 423 (42.7), 249 (45.9), 206 (100), 179 (57.4), 120 (71.7); Anal. Calcd. For $C_{23}H_{25}N_3O_3S$ (423.53); % C, 65.23; H, 5.95; N, 9.92, Found: % C, 64.97; H, 5.86; N, 10.21.

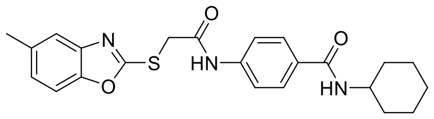

IR

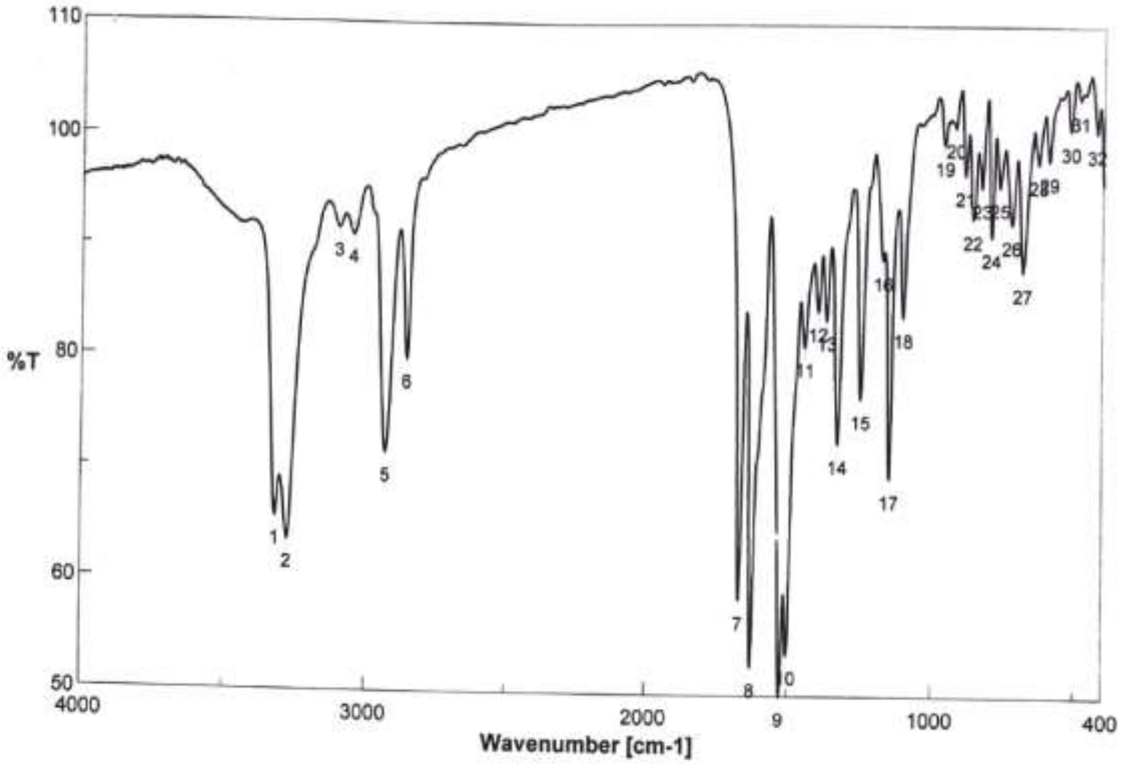

Accumulation	Auto (32)
Resolution	4 cm-1
Zero Filling	ON
Apodization	Cosine
Gain	Auto (2)
Scanning Speed	Auto (2 mm/sec)
Date/Time	5/9/2021 0:00PM
Update	5/9/2021 0:01PM
Operator	IR
File Name	Memory#65
Sample Name	MBA-8
Comment	

Mass

Cairo University
Micro Analytical Center

DI Analysis
Shimadzu Qp-2010 Plus

Sample Information

Analyzed by	: Dr. Mai Younis
Analyzed	: 01/01/2007 06:03:14 ص
Sample Name	: 7
Sample ID	:
Customer Name	: Dr.Radwan Saeed - Pharmacy - Helwan
Data File	: C:\GCMSsolution\Data\Project1\7.QGD
Org Data File	: C:\GCMSsolution\Data\Project1\7.QGD
Method File	: C:\GCMSsolution\Data\Project1\High Temperature Op
Org Method File	: C:\GCMSsolution\Data\Project1\High Temperature Op
Report File	:
Tuning File	: C:\GCMSsolution\System\Tune1_default.qgt
$EndIf$Modified by	: Dr. Mai Younis
Modified	: 01/01/2007 06:07:33 ص

Method

====== Analytical Line 1 ======

IonSourceTemp	:250.00 °C
[MS Table]	
--Group 1 - Event 1--	
Start Time	:0.00min
End Time	:10.00min
ACQ Mode	:Scan
Event Time	:0.50sec
Scan Speed	:1000
Start m/z	:50.00
End m/z	:500.00
Electron Voltage	: 70 eV
Ionization Mode	: EI

C:\GCMSsolution\Data\Project1\7.QGD

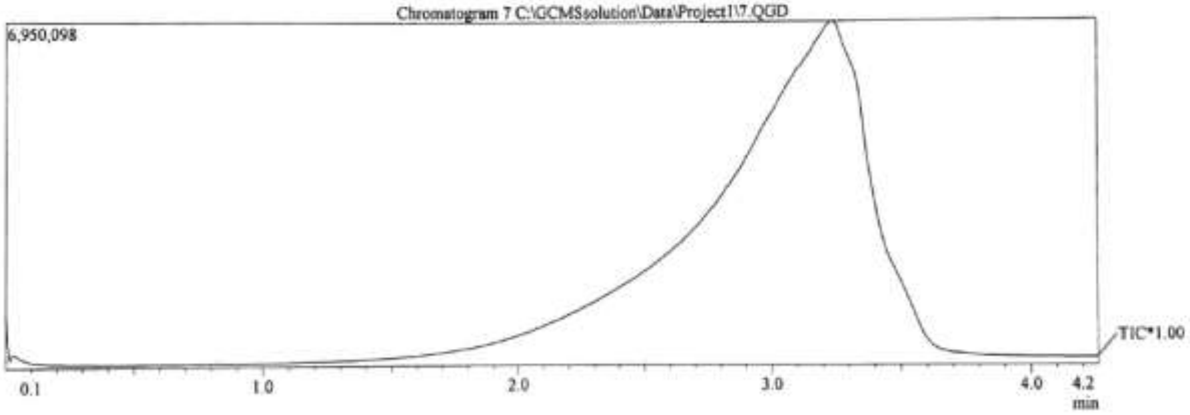

Spectrum

Line#:1 R.Time:3.2(Scan#:387)
MassPeaks:345
RawMode:Single 3.2(387) BasePeak:206(473977)
BG Mode:None Group 1 - Event 1

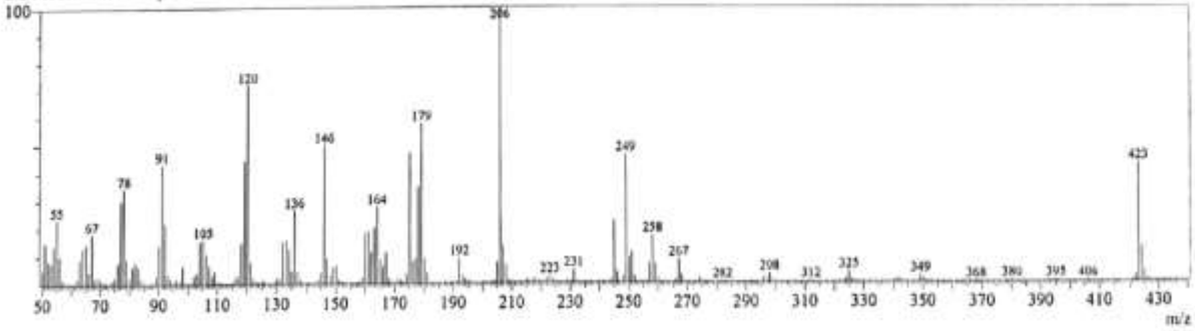

1HNMR

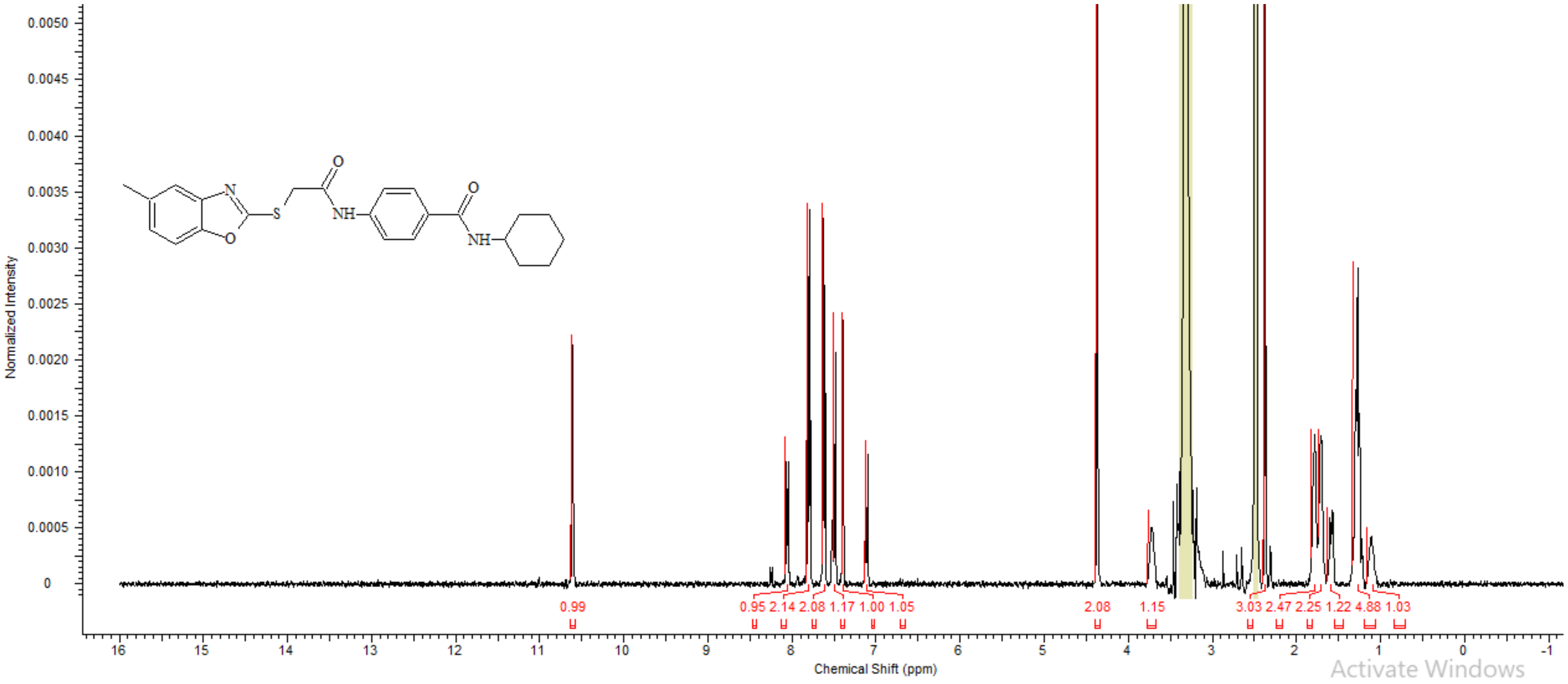
Normalized Intensity
Chemical Shift (ppm)
0.99
0.95 2.14 2.08 1.17 1.00 1.05
2.08
1.15
3.03 2.47 2.25 1.22 4.88 1.03
NH
NH
O
O
N
O
S

13CNMR

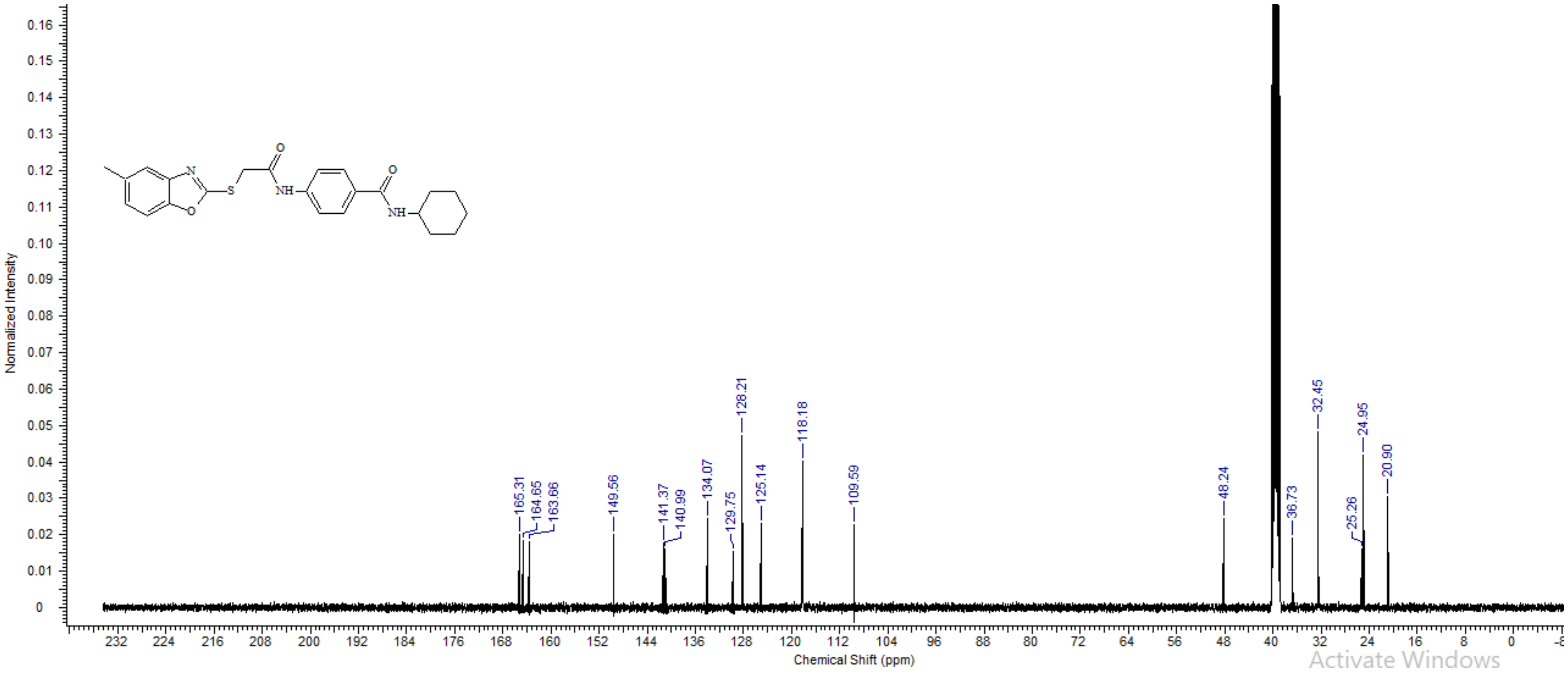

Normalized Intensity
Chemical Shift (ppm)
165.31
164.65
163.66
149.56
141.37
140.99
134.07
129.75
128.21
125.14
118.18
109.59
48.24
36.73
32.45
25.26
24.95
20.90
Activate Windows

1.6. 4-(2-(5-methylbenzo[d]oxazol-2-ylthio)acetamido)-N-phenylbenzamide (6):

Off-white powder (yield 75%); m.p. 240-242 ºC; IR ($\upsilon_{max.}$/cm^{-1}) 3282 (N-H), 1671 and 1641(2*C=O); ^{1}H NMR (400 MHz, DMSO-d$_6$) δ 10.70 (s, 1H, exchangeable with D_2O, (*NHCOCH$_2$S-)), 10.11 (s, 1H, exchangeable with D_2O), 7.94 (d, *J* = 8.35 Hz, 2H), 7.71 (d, *J* = 8.35 Hz, 2H), 7.74 (d, *J* = 7.91 Hz, 2H), 7.50 (d, *J* = 8.35 Hz, 1H), 7.40 (s, 1H), 7.32 (t, *J* = 7.91 Hz, 2H), 7.04 - 7.14 (m, 2H), 4.40 (s, 2H), 2.38 (s, 3H). ^{13}C NMR (101 MHz, DMSO-d$_6$) δ 165.5, 164.8, 163.7, 149.6, 141.6, 141.4, 139.2, 134.1, 129.7, 128.7, 128.6, 125.2, 123.5, 120.3, 118.4, 118.2, 109.6, 36.8, 20.9. MS (m/z (R.I. %)): [M]$^+$ 417 (27.5), 325 (26.2), 243 (12.0), 206 (60.0), 120 (100); Anal. Calcd. For $C_{23}H_{19}N_3O_3S$ (417.48); % C, 66.17; H, 4.59; N, 10.07, Found: % C, 65.97; H, 4.86; N, 10.39.

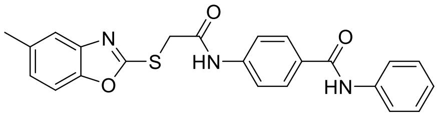

IR

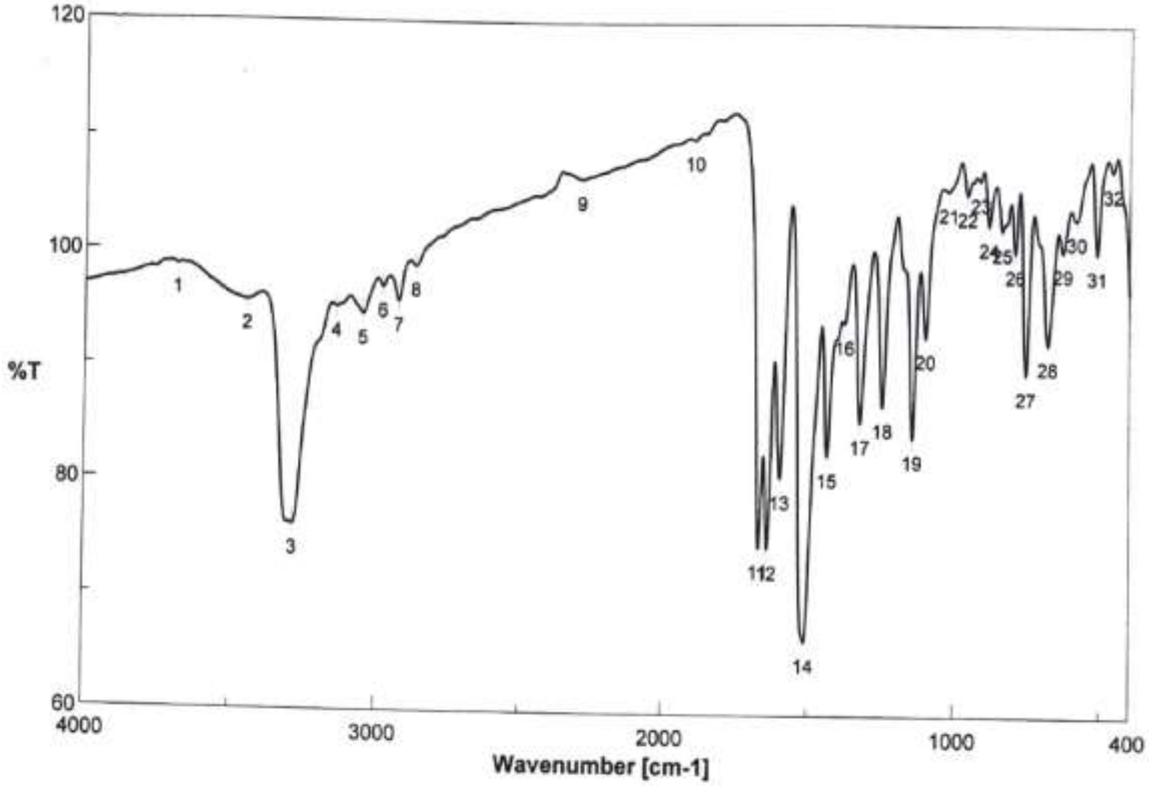

Accumulation	Auto (30)
Resolution	4 cm-1
Zero Filling	ON
Apodization	Cosine
Gain	Auto (2)
Scanning Speed	Auto (2 mm/sec)
Date/Time	5/9/2021 0:05PM
Update	5/9/2021 0:05PM
Operator	IR
File Name	Memory#76
Sample Name	MBA-1
Comment	

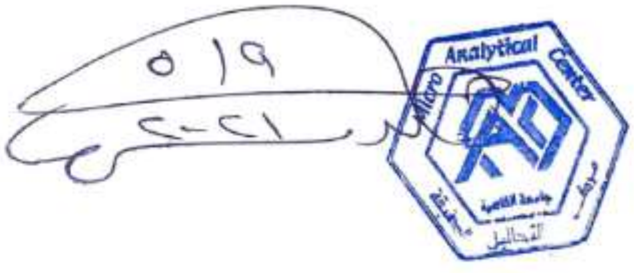

Mass

Cairo University
Micro Analytical Center

DI Analysis
Shimadzu Qp-2010 Plus

Sample Information		Method	
Analyzed by	: Dr. Mai Younis		
Analyzed	: 01/01/2007 05:29:42 ص	------ Analytical Line 1 ------	
Sample Name	: 2	IonSourceTemp	:250.00 °C
Sample ID	:	[MS Table]	
Customer Name	: Dr.Radwan Saeed - Pharmacy - Helwan	--Group 1 - Event 1--	
Data File	: C:\GCMSsolution\Data\Project1\2.QGD	Start Time	:0.00min
Org Data File	: C:\GCMSsolution\Data\Project1\2.QGD	End Time	:10.00min
Method File	: C:\GCMSsolution\Data\Project1\High Temperature Op	ACQ Mode	:Scan
Org Method File	: C:\GCMSsolution\Data\Project1\High Temperature Op	Event Time	:0.50sec
Report File	:	Scan Speed	:1000
Tuning File	: C:\GCMSsolution\System\Tune1_default.qgt	Start m/z	:50.00
$EndIf$Modified by	: Dr. Mai Younis	End m/z	:500.00
Modified	: 01/01/2007 05:35:24 ص		
		Electron Voltage	: 70 eV
		Ionization Mode	: EI

C:\GCMSsolution\Data\Project1\2.QGD

Chromatogram 2 C:\GCMSsolution\Data\Project1\2.QGD

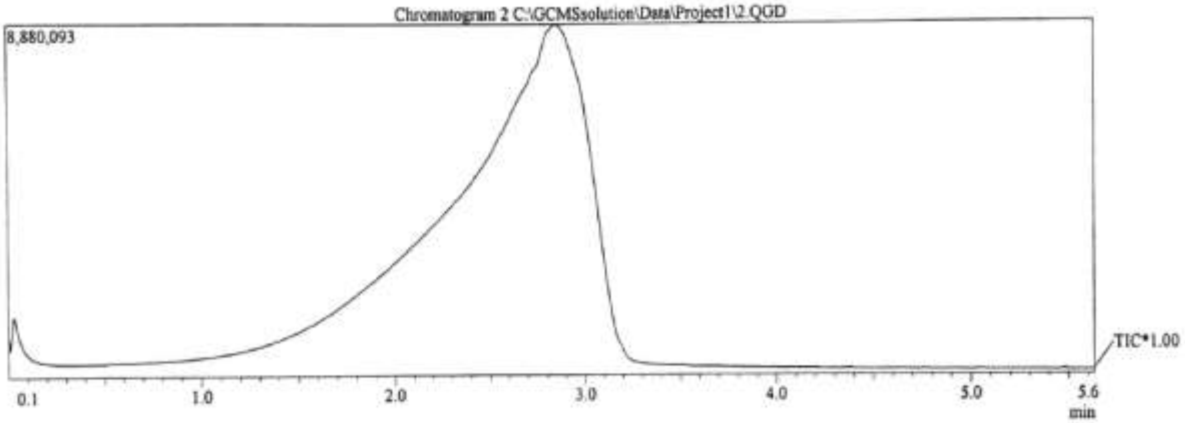

Spectrum

Line#:1 R.Time:2.7(Scan#:330)
MassPeaks:322
RawMode:Single 2.7(330) BasePeak:120(1059105)
BG Mode:None Group 1 - Event 1

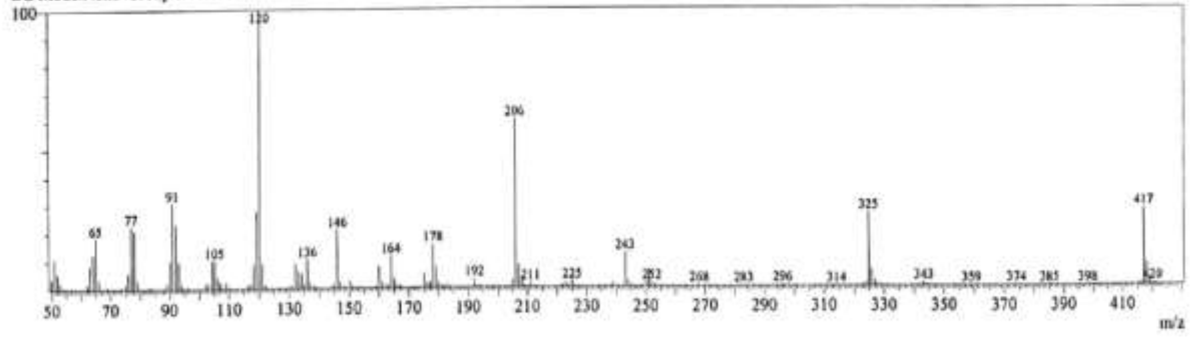

1HNMR

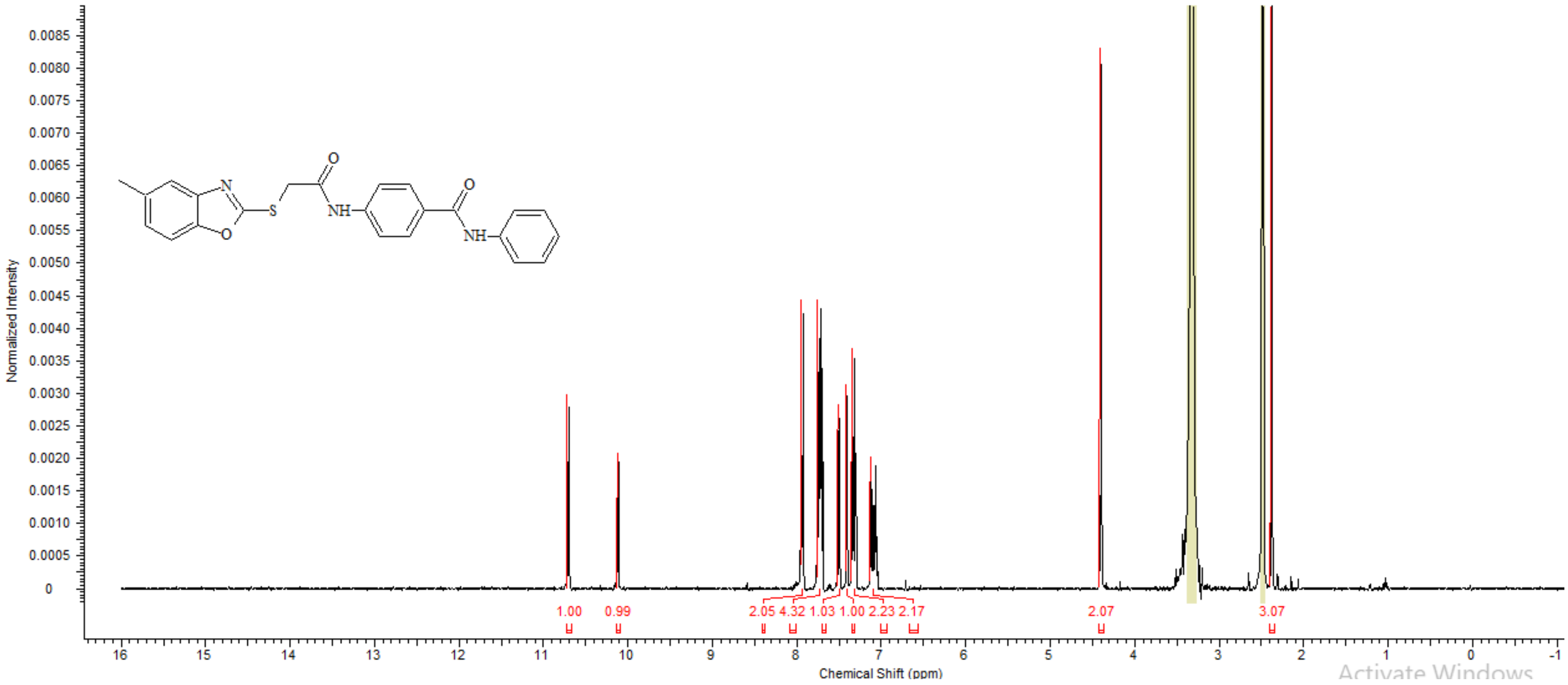

Normalized Intensity
Chemical Shift (ppm)
1.00
0.99
2.05
4.32
1.03
1.00
2.23
2.17
2.07
3.07
N
O
S
O
NH
O
NH

13CNMR

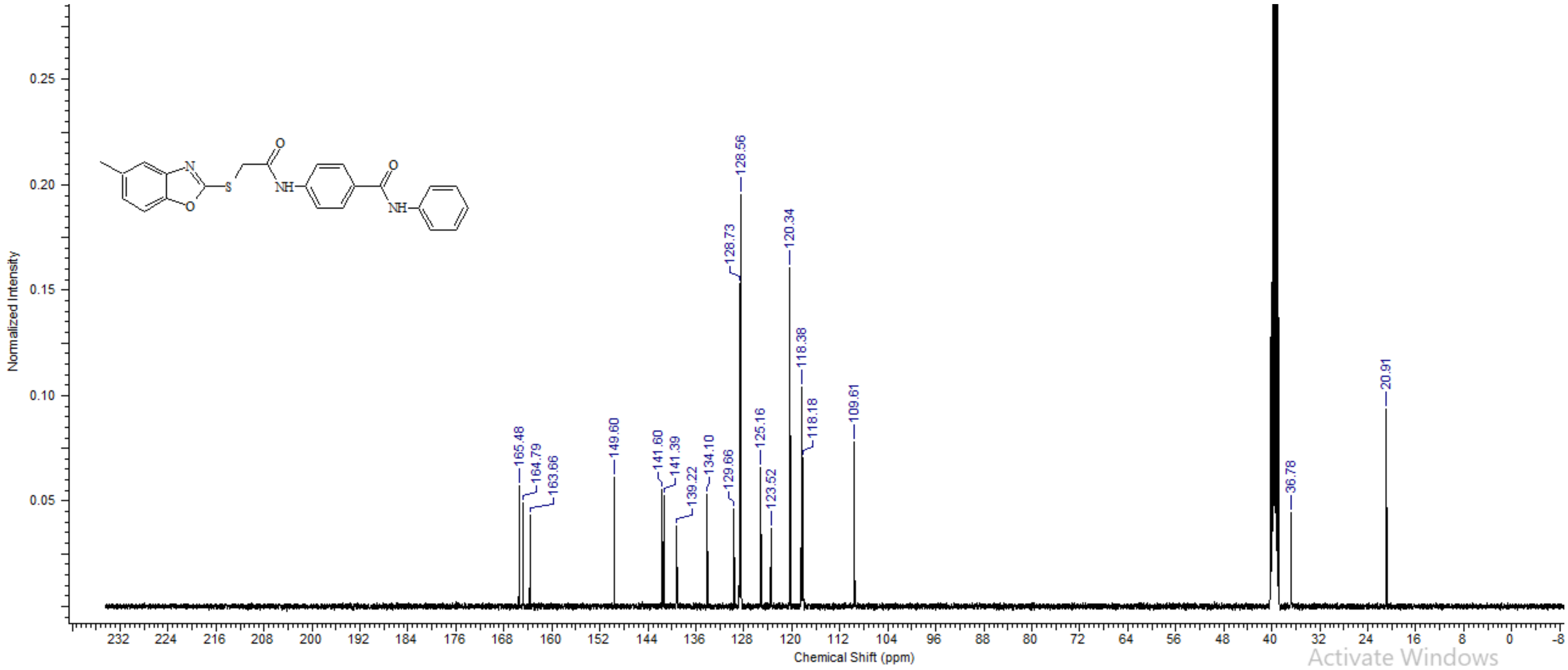

165.48
164.79
163.66
149.60
141.60
141.39
139.22
134.10
129.66
128.73
128.56
125.16
123.52
120.34
118.38
118.18
109.61
36.78
20.91
Normalized Intensity
Chemical Shift (ppm)
Activate Windows

1.7. 4-(2-(5-methylbenzo[d]oxazol-2-ylthio)acetamido)-N-(4-chlorophenyl)benzamide (7):

Off-white powder (yield 73%); m.p. 265-267°C; IR ($\upsilon_{max.}$/cm^{-1}) 3358 and 3317 (2*N-H), 1656 (br.C=O); ^{1}H NMR (400 MHz, DMSO-d$_6$) δ 10.71 (s, 1H, exchangeable with D_2O, (*NHCOCH$_2$S-)), 10.24 (s, 1H, exchangeable with D_2O), 7.93 (d, *J* = 8.79 Hz, 2H), 7.78 (d, *J* = 8.79 Hz, 2H), 7.71 (d, *J* = 8.79 Hz, 2H), 7.50 (d, *J* = 7.91 Hz, 1H), 7.40 (br. s., 1H), 7.38 (d, *J* = 8.79 Hz, 2H), 7.12 (d, *J* = 8.35 Hz, 1H), 4.39 (s, 2H), 2.37 (s, 3H). ^{13}C NMR (101 MHz, DMSO-d$_6$) δ 165.5, 165.4, 164.9, 156.8, 149.6, 141.8, 141.4, 138.2, 134.1, 129.4, 128.8, 128.5, 125.2, 121.8, 118.4, 118.2, 109.6, 36.8, 20.9. MS (m/z (R.I. %)): [M]$^{+}$ 451 (18.4), [M+2]$^{+}$ 453 (7.5), 325 (42.8), 206 (60.4), 120 (100); Anal. Calcd. For $C_{23}H_{18}ClN_3O_3S$ (451.92); % C, 61.13; H, 4.01; N, 9.30, Found: % C, 61.40; H, 4.25; N, 9.18.

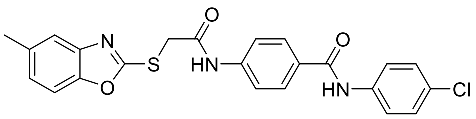

IR

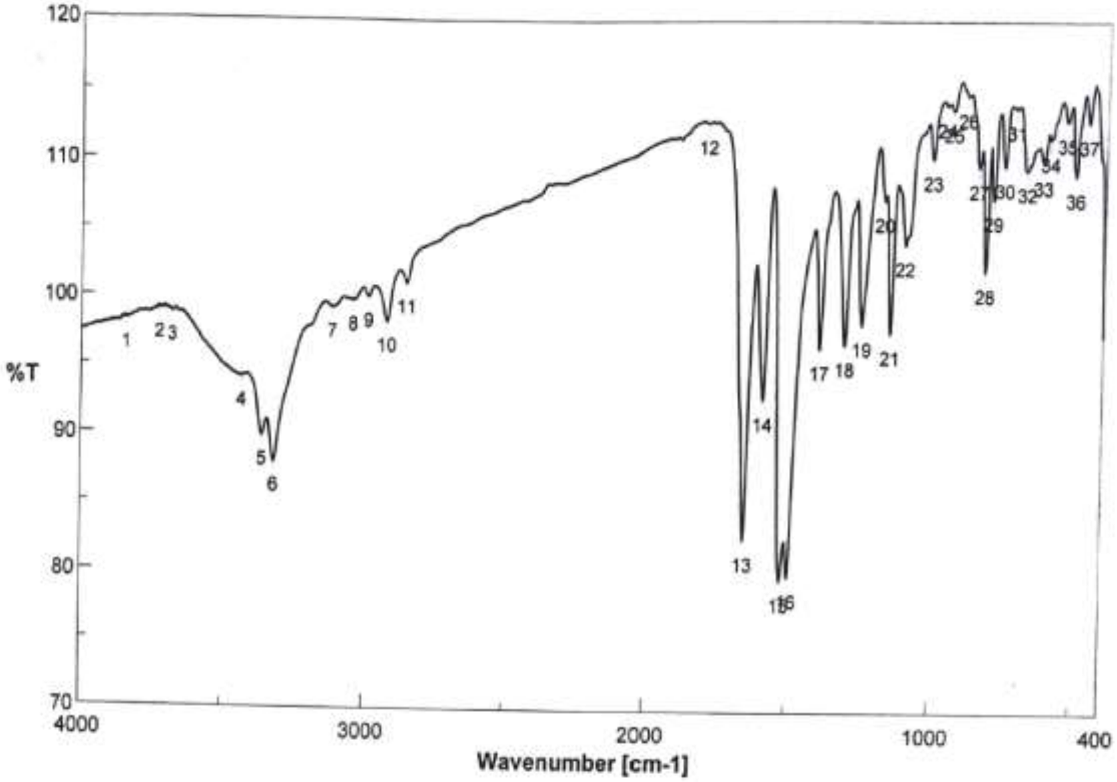

Accumulation	Auto (28)
Resolution	4 cm-1
Zero Filling	ON
Apodization	Cosine
Gain	Auto (2)
Scanning Speed	Auto (2 mm/sec)
Date/Time	5/9/2021 0:02PM
Update	5/9/2021 0:02PM
Operator	IR
File Name	Memory#69
Sample Name	MBA-3
Comment	

Mass

Cairo University
Micro Analytical Center

DI Analysis
Shimadzu Qp-2010 Plus

Sample Information

Analyzed by	: Dr. Mai Younis
Analyzed	: 01/01/2007 05:43:50 ص
Sample Name	: 4
Sample ID	:
Customer Name	: Dr.Radwan Saeed - Pharmacy - Helwan
Data File	: C:\GCMSsolution\Data\Project1\4.QGD
Org Data File	: C:\GCMSsolution\Data\Project1\4.QGD
Method File	: C:\GCMSsolution\Data\Project1\High Temperature Op
Org Method File	: C:\GCMSsolution\Data\Project1\High Temperature Op
Report File	:
Tuning File	: C:\GCMSsolution\System\Tune1_default.qgt
$EndIf$Modified by	: Dr. Mai Younis
Modified	: 01/01/2007 05:48:23 ص

Method

====== Analytical Line 1 ======

IonSourceTemp	:250.00 °C
[MS Table]	
--Group 1 - Event 1--	
Start Time	:0.00min
End Time	:10.00min
ACQ Mode	:Scan
Event Time	:0.50sec
Scan Speed	:1000
Start m/z	:50.00
End m/z	:500.00
Electron Voltage	: 70 eV
Ionization Mode	: EI

C:\GCMSsolution\Data\Project1\4.QGD

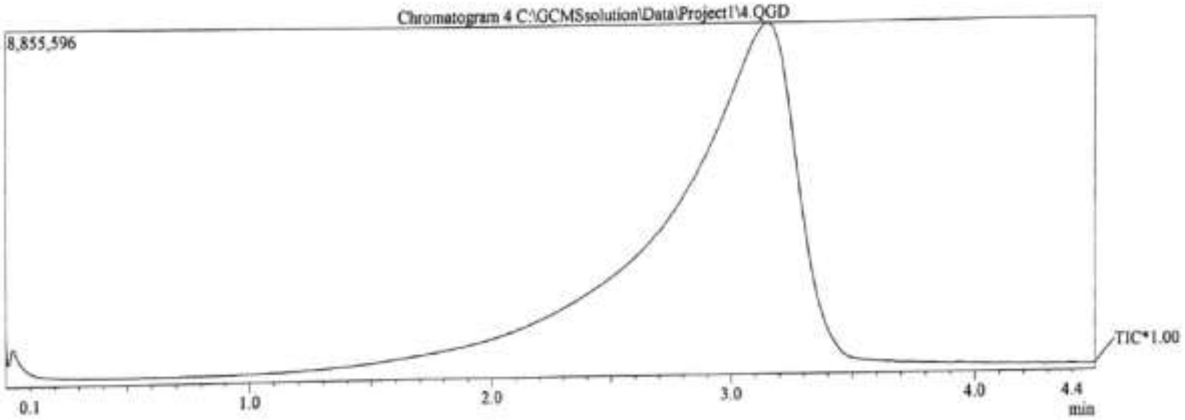

Spectrum

Line#:1 R.Time:3.1(Scan#:375)
MassPeaks:396
RawMode:Single 3.1(375) BasePeak:120(1119566)
BG Mode:None Group 1 - Event 1

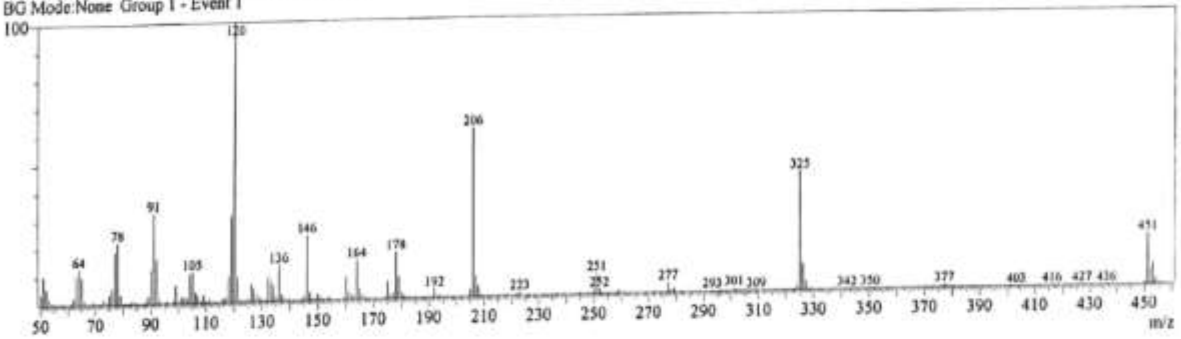

1HNMR

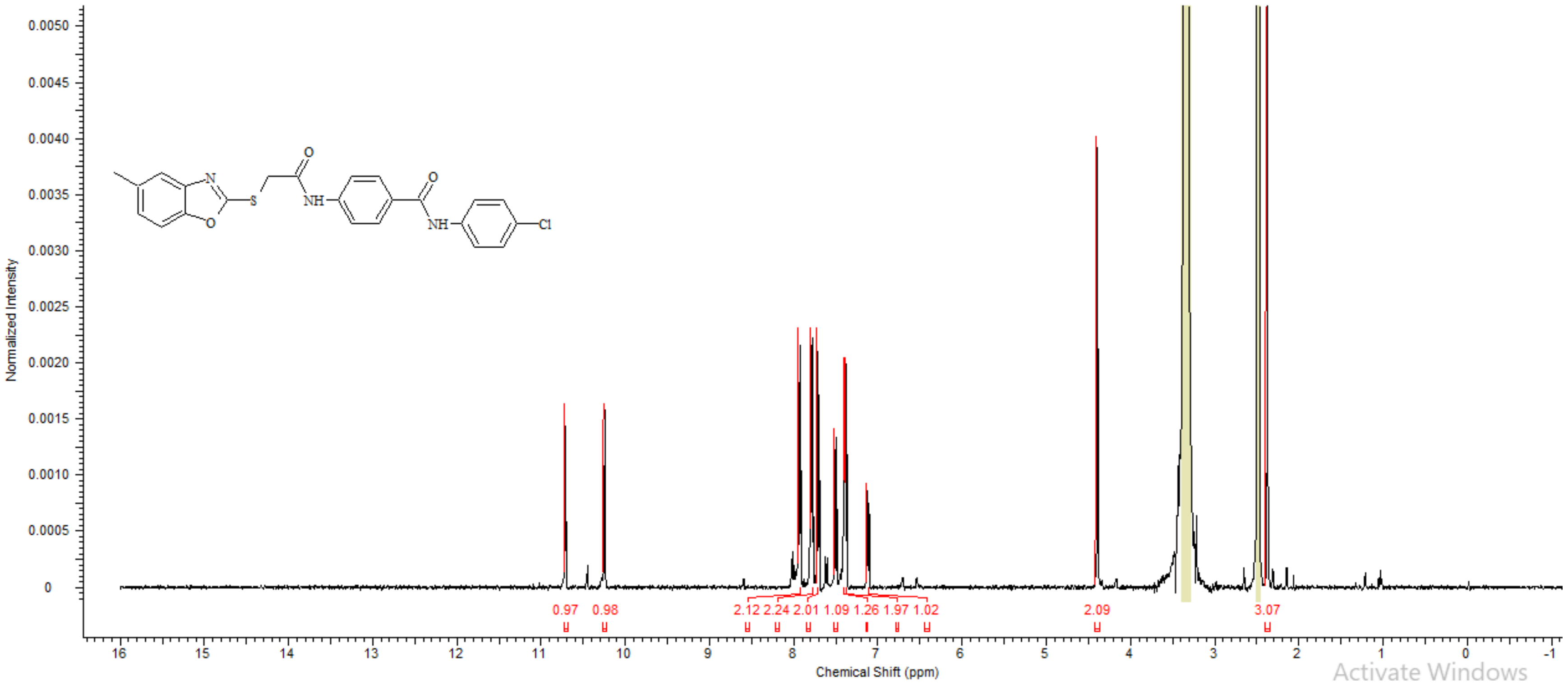

Normalized Intensity
Chemical Shift (ppm)
N
O
S
O
NH
O
NH
Cl
0.97
0.98
2.12
2.24
2.01
1.09
1.26
1.97
1.02
2.09
3.07
Activate Windows

13CNMR

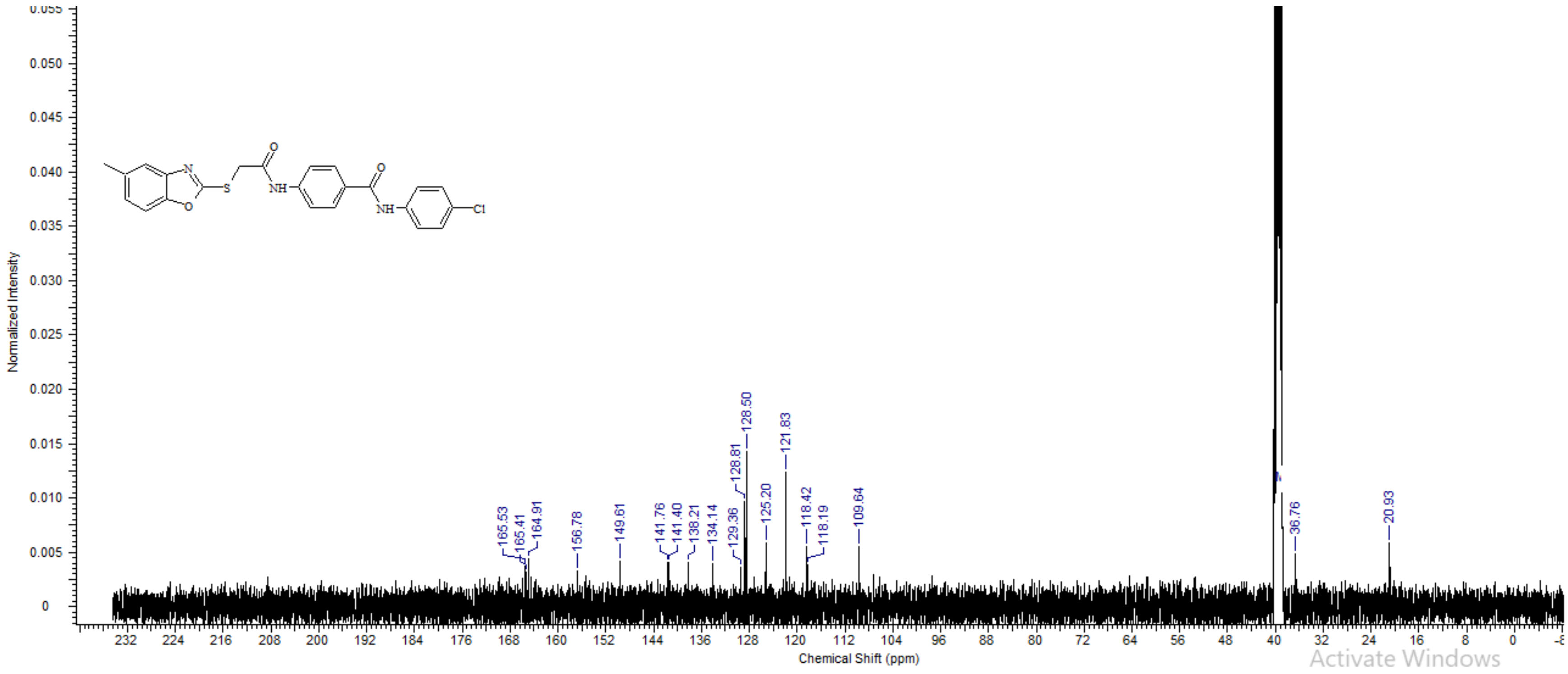

Normalized Intensity
Chemical Shift (ppm)
165.53
165.41
164.91
156.78
149.61
141.76
141.40
138.21
134.14
129.36
128.81
128.50
125.20
121.83
118.42
118.19
109.64
36.76
20.93
NH
NH
Cl

1.8. 4-(2-(5-methylbenzo[d]oxazol-2-ylthio)acetamido)-N-(4-methoxyphenyl)benzamide (8):

Yellow powder (yield 80%); m.p. 258-260°C; IR ($\upsilon_{max.}$/cm^{-1}) 3294 (N-H), 1662 and 1642 (2*C=O); ^{1}H NMR (400 MHz, DMSO-d_6) δ 10.68 (br. s., 1H, D_2O exchangeable, (*NHCOCH_2S)), 9.99 (br. s., 1H, D_2O exchangeable), 7.92 (d, *J* = 8.35 Hz, 2H), 7.69 (d, *J* = 8.35 Hz, 2H), 7.63 (d, *J* = 8.79 Hz, 2H), 7.50 (d, *J* = 8.35 Hz, 1H), 7.40 (br. s., 1H), 7.12 (d, *J* = 8.35 Hz, 1H), 6.90 (d, *J* = 8.79 Hz, 2H), 4.39 (br. s., 2H), 3.72 (s, 3H), 2.37 (br. s., 3H). ^{13}C NMR (101 MHz, DMSO-d_6) δ 165.4, 164.3, 163.6, 155.4, 149.6, 141.4, 141.4, 134.1, 132.3, 129.7, 128.6, 125.1, 121.9, 118.3, 118.2, 113.7, 109.6, 55.1, 36.8, 20.9. MS (m/z (R.I. %)): $[M]^{+}$ 447 (51.8), 325 (29.5), 206 (48.0), 120 (100); Anal. Calcd. for $C_{24}H_{21}N_3O_4S$ (447.50); % C, 64.41; H, 4.73; N, 9.39, Found: % C, 64.65; H, 4.98; N, 9.61.

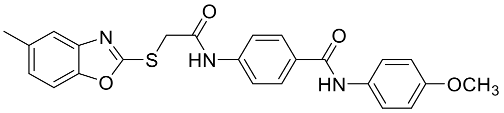

IR

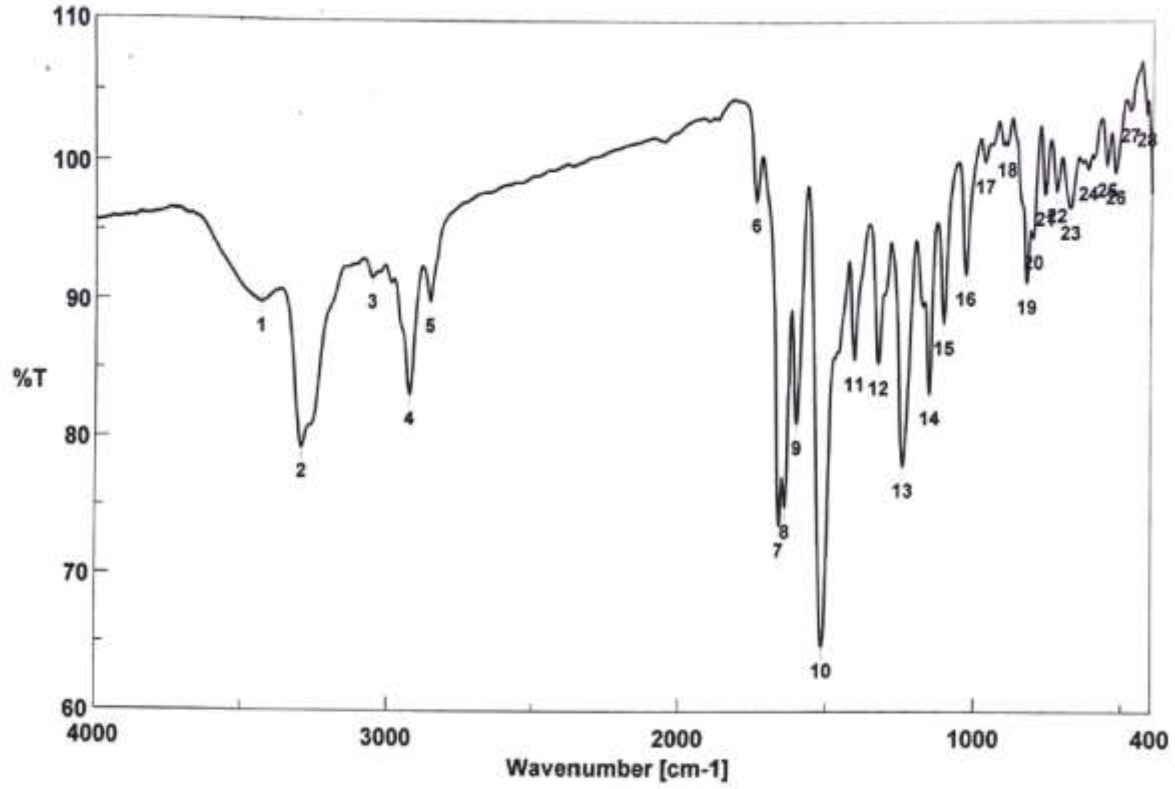

Accumulation	16
Resolution	4 cm-1
Zero Filling	ON
Apodization	Cosine
Gain	Auto (2)
Scanning Speed	Auto (2 mm/sec)
Date/Time	8/22/2021 1:44PM
Update	8/22/2021 1:45PM
Operator	IR
File Name	Memory#72
Sample Name	MBA -19
Comment	

Mass

Cairo University
Micro Analytical Center

DI Analysis
Shimadzu Qp-2010 Plus

Sample Information

Analyzed by	: Dr. Mai Younis
Analyzed	: 01/01/2007 10:55:55 م
Sample Name	: 18
Sample ID	:
Customer Name	: Dr.Radwan Saeed - Pharmacy - Helwan
Data File	: C:\GCMSsolution\Data\Project1\18.QGD
Org Data File	: C:\GCMSsolution\Data\Project1\18.QGD
Method File	: C:\GCMSsolution\Data\Project1\High Temperature Op
Org Method File	: C:\GCMSsolution\Data\Project1\High Temperature Op
Report File	:
Tuning File	: C:\GCMSsolution\System\Tune1_default.qgt
$EndIf$Modified by	: Dr. Mai Younis
Modified	: 01/01/2007 11:01:01 م

Method

—— Analytical Line 1 ——

IonSourceTemp	:250.00 °C
[MS Table]	
--Group 1 - Event 1--	
Start Time	:0.00min
End Time	:10.00min
ACQ Mode	:Scan
Event Time	:0.50sec
Scan Speed	:1250
Start m/z	:50.00
End m/z	:600.00
Electron Voltage	: 70 eV
Ionization Mode	: EI

C:\GCMSsolution\Data\Project1\18.QGD

Chromatogram 18 C:\GCMSsolution\Data\Project1\18.QGD

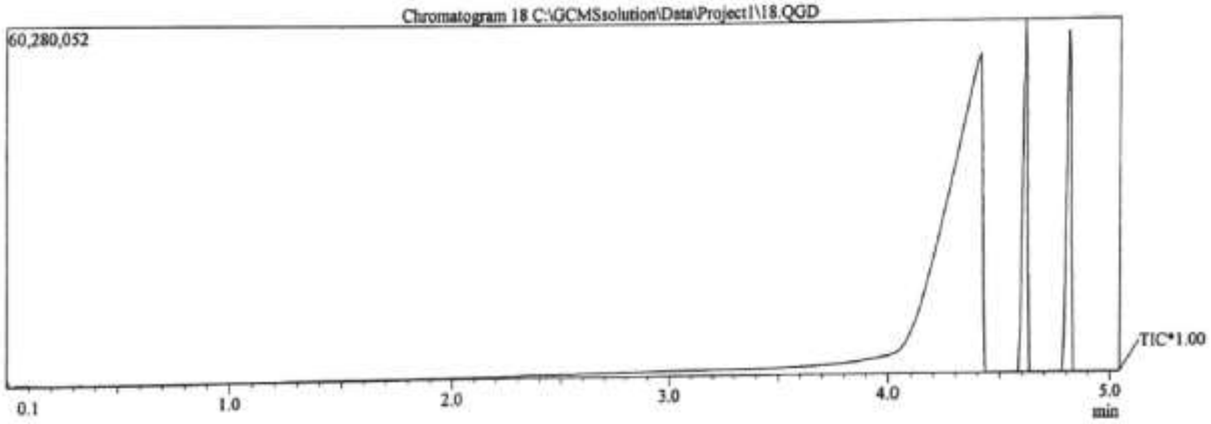

Spectrum

Line#:1 R.Time:4.4(Scan#:528)
MassPeaks:410
RawMode:Single 4.4(528) BasePeak:120(6816756)
BG Mode:None Group 1 - Event 1

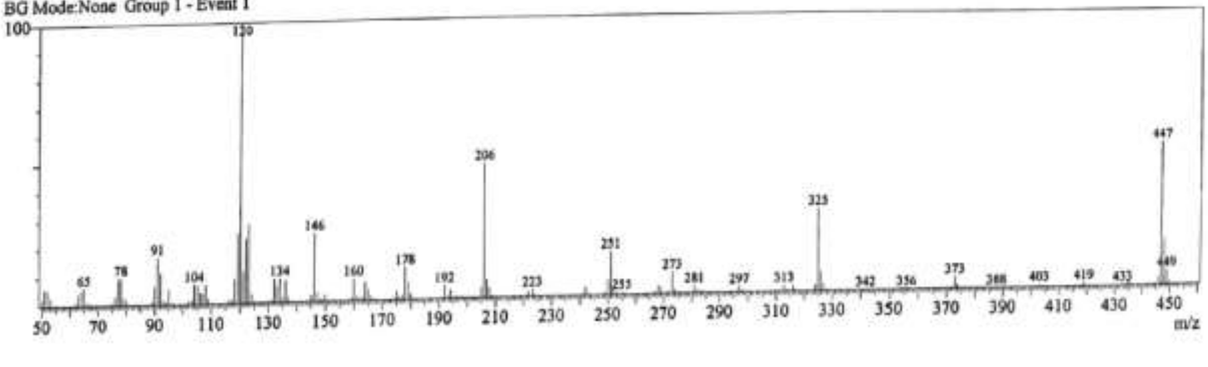

1HNMR

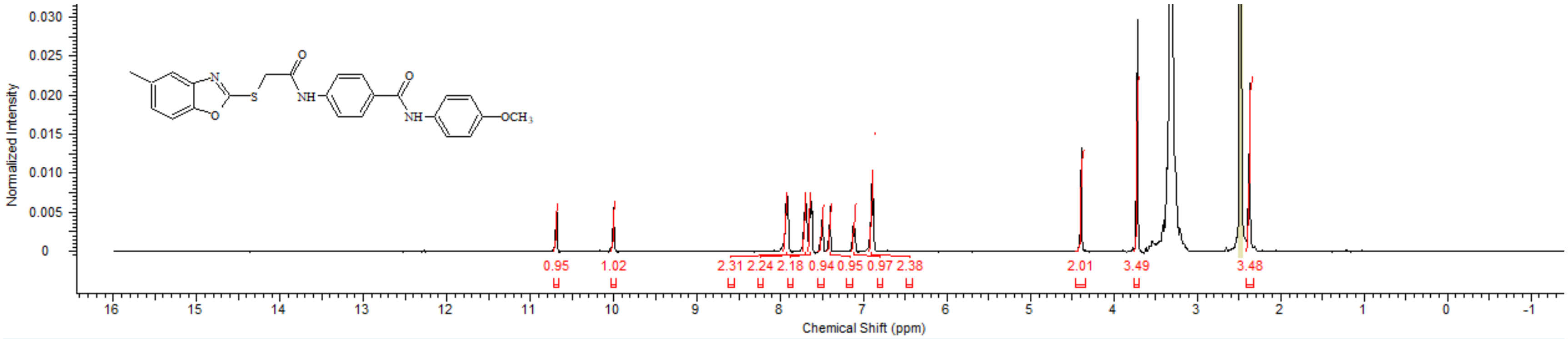

Normalized Intensity
OCH3
NH
NH
S
N
O
0.95
1.02
2.31 2.24 2.18 0.94 0.95 0.97 2.38
2.01
3.49
3.48
Chemical Shift (ppm)

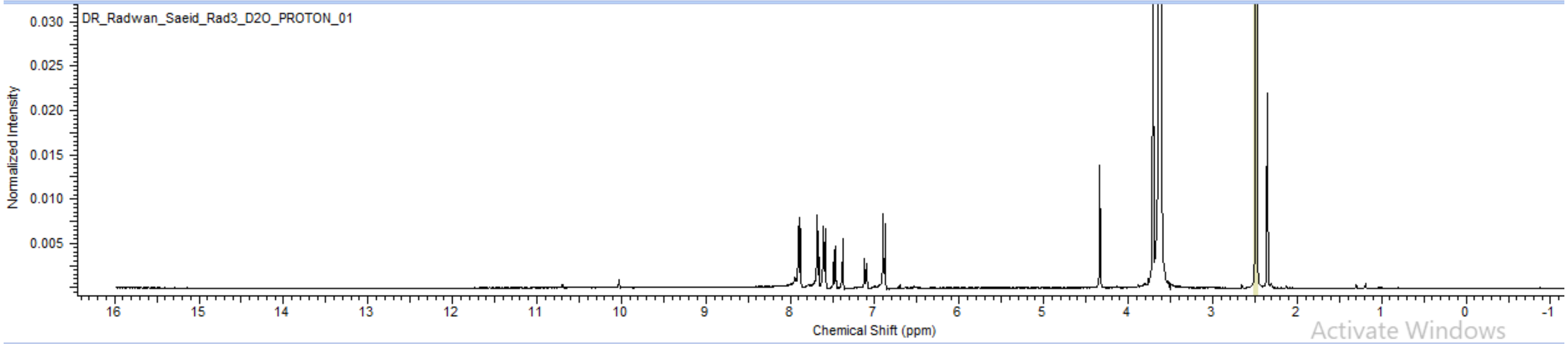

DR_Radwan_Saeid_Rad3_D2O_PROTON_01
Normalized Intensity
Chemical Shift (ppm)
Activate Windows

13CNMR

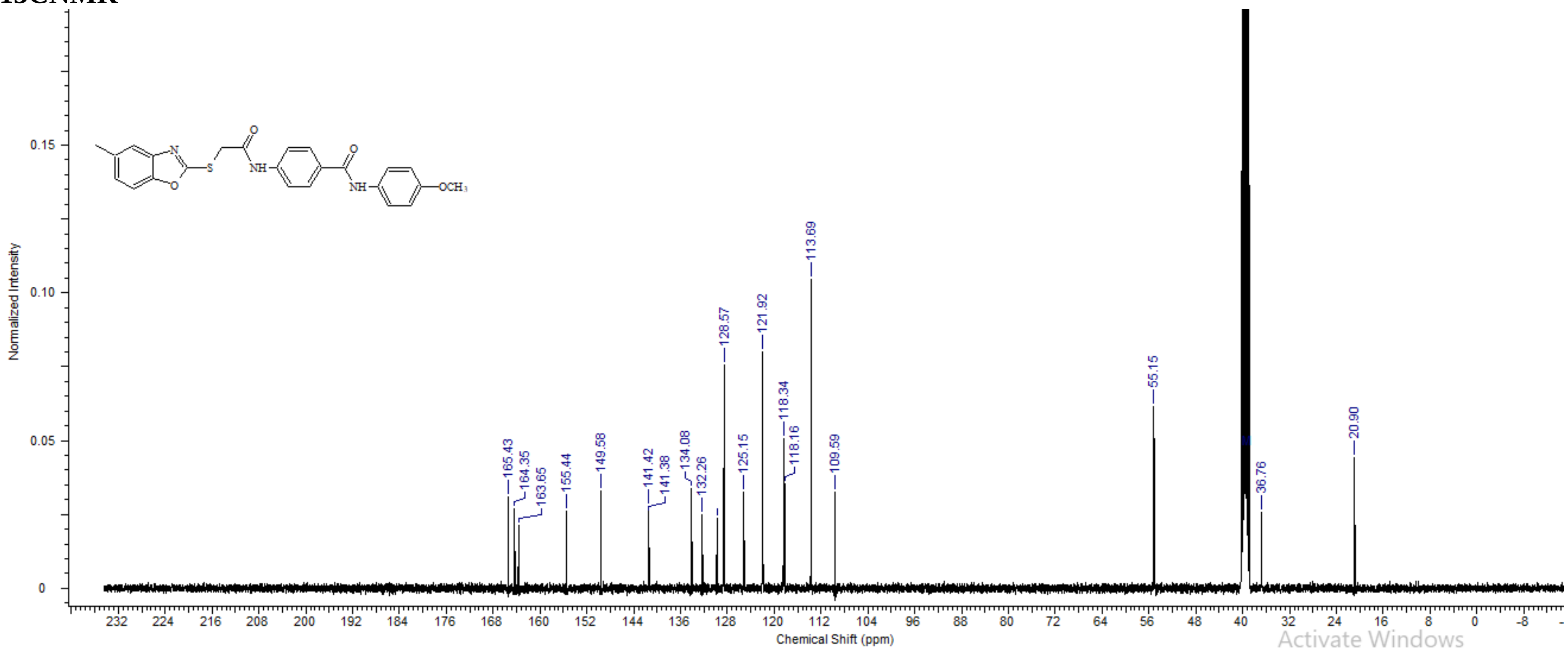

Activate Windows

1.9. 4-(2-(5-chlorobenzo[d]oxazol-2-ylthio)acetamido)-N-cyclohexylbenzamide (9):

Light brown powder (yield 75%); m.p. 260-262°C; IR ($\upsilon_{max.}$/cm^{-1}) 3314 and 3269 (2*N-H), 2933 (C-H aliphatic), 1670 and 1627(2*C=O); ^{1}H NMR (400 MHz, DMSO-d$_6$) δ 10.63 (s, 1H, exchangeable with D_2O, (*NHCOCH$_2$S-)), 8.06 (d, *J* = 7.91 Hz, 1H, exchangeable with D_2O), 7.80 (d, *J* = 8.79 Hz, 2H), 7.72 (d, *J* = 1.76 Hz, 1H), 7.68 (d, *J* = 8.79 Hz, 1H), 7.61 (d, *J* = 8.35 Hz, 2H), 7.35 (dd, *J* = 2.20, 8.79 Hz, 1H), 4.40 (s, 2H), 3.66 - 3.77 (m, 1H), 1.75 - 1.81 (m, 2H), 1.67 - 1.74 (m, 2H), 1.58 (d, *J* = 10.99 Hz, 1H), 1.20 - 1.34 (m, 4H). ^{13}C NMR (101 MHz, DMSO-d$_6$) δ 165.9, 165.1, 164.6, 150.1, 142.5, 140.9, 129.8, 129.0, 128.2, 124.3, 118.2, 118.0, 111.5, 48.2, 36.9, 32.4, 25.3, 24.9. MS (m/z (R.I. %)): [M]$^+$ 443 (16.3), [M+2]$^+$ 445 (6.0), 245 (27.8), 226 (55.6), 120 (100);Anal. Calcd. for $C_{22}H_{22}ClN_3O_3S$ (443.94); % C, 59.52; H, 4.99; N, 9.47, Found: % C, 59.75; H, 5.11; N, 9.71.

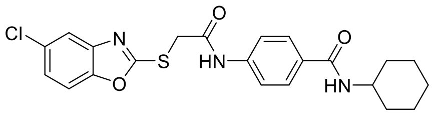

IR

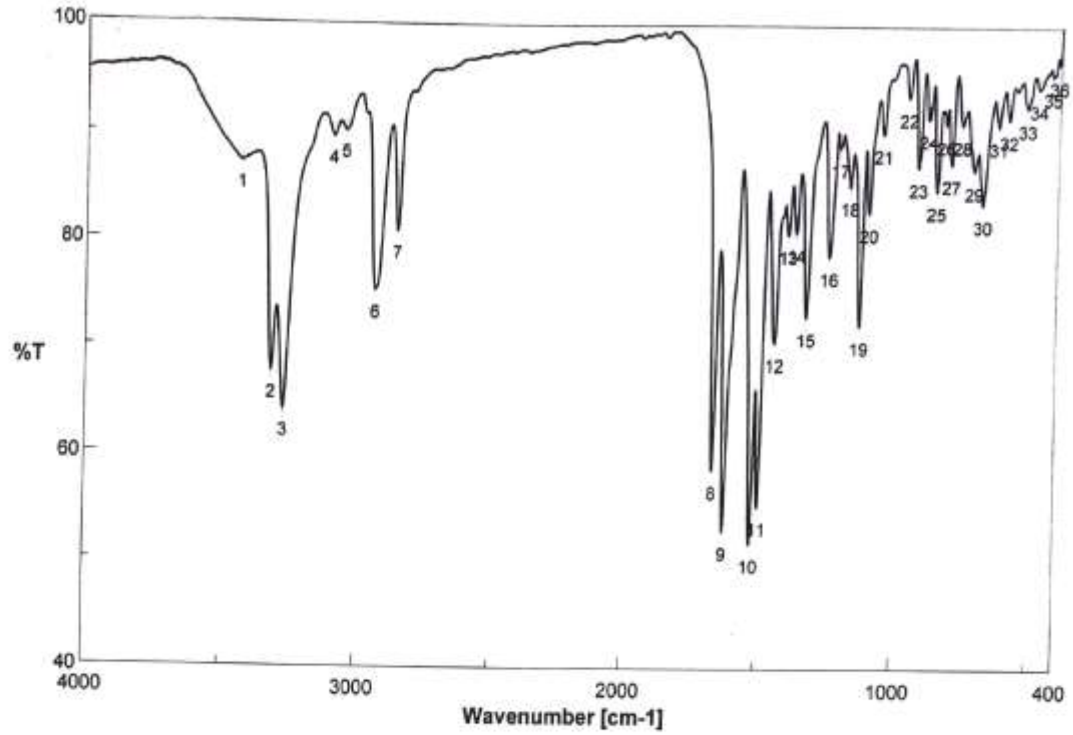

Accumulation	Auto (30)
Resolution	4 cm-1
Zero Filling	ON
Apodization	Cosine
Gain	Auto (2)
Scanning Speed	Auto (2 mm/sec)
Date/Time	5/9/2021 0:15PM
Update	5/9/2021 0:17PM
Operator	IR
File Name	Memory#95
Sample Name	CBA -8
Comment	

Mass

Cairo University
Micro Analytical Center

DI Analysis
Shimadzu Qp-2010 Plus

Sample Information

Analyzed by	: Dr. Mai Younis
Analyzed	: 01/01/2007 05:57:41 ص
Sample Name	: 6
Sample ID	:
Customer Name	: Dr.Radwan Saeed - Pharmacy - Helwan
Data File	: C:\GCMSsolution\Data\Project1\6.QGD
Org Data File	: C:\GCMSsolution\Data\Project1\6.QGD
Method File	: C:\GCMSsolution\Data\Project1\High Temperature Op
Org Method File	: C:\GCMSsolution\Data\Project1\High Temperature Op
Report File	:
Tuning File	: C:\GCMSsolution\System\Tune1_default.qgt
$EndIf$Modified by	: Dr. Mai Younis
Modified	: 01/01/2007 06:01:06 ص

Method

====== Analytical Line 1 ======

IonSourceTemp	:250.00 °C
[MS Table]	
--Group 1 - Event 1--	
Start Time	:0.00min
End Time	:10.00min
ACQ Mode	:Scan
Event Time	:0.50sec
Scan Speed	:1000
Start m/z	:50.00
End m/z	:500.00
Electron Voltage	: 70 eV
Ionization Mode	: EI

C:\GCMSsolution\Data\Project1\6.QGD

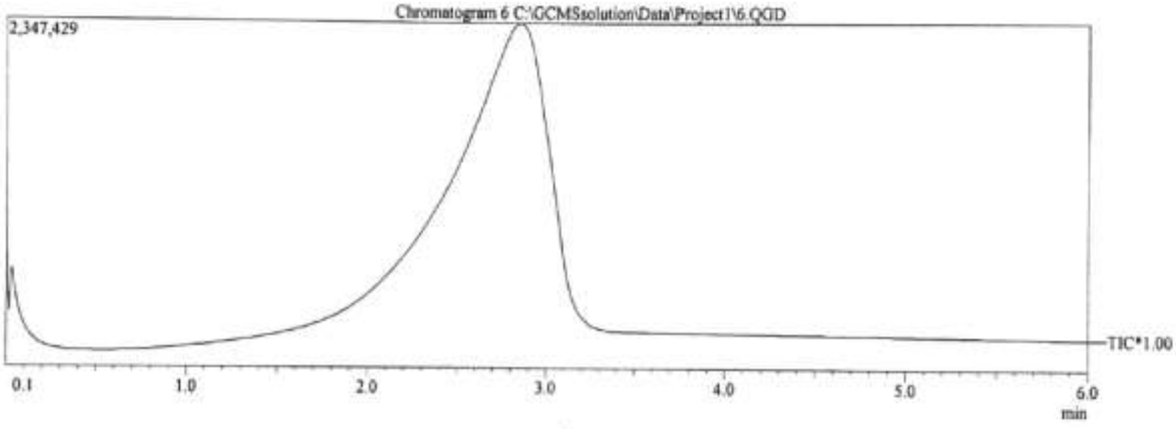

Spectrum

Line#:1 R.Time:2.8(Scan#:338)
MassPeaks:339
RawMode:Single 2.8(338) BasePeak:120(169585)
BG Mode:None Group 1 - Event 1

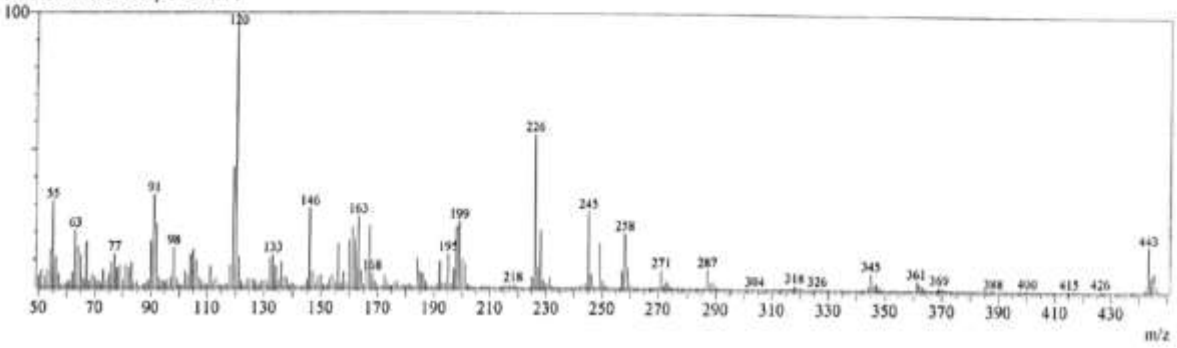

1HNMR

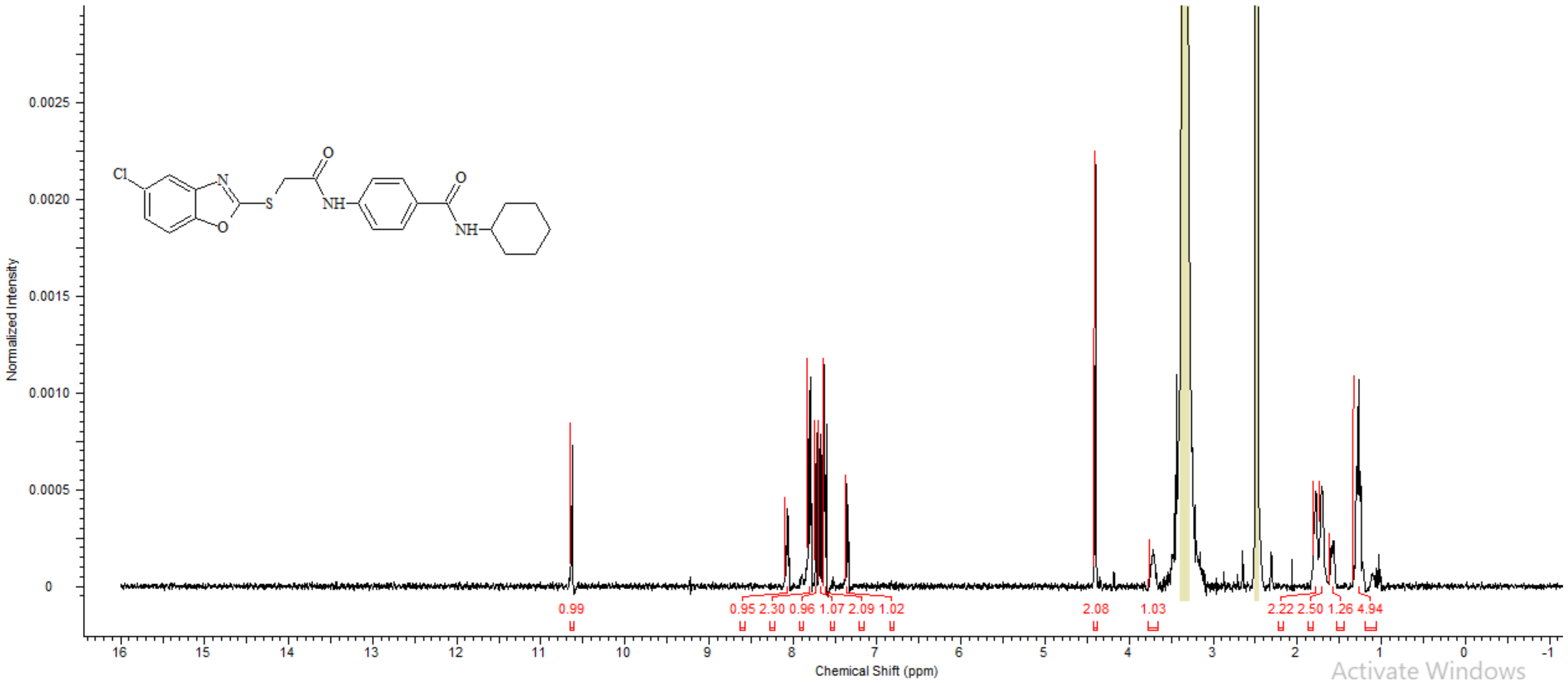
Normalized Intensity
Chemical Shift (ppm)
0.99
0.95 2.30 0.96 1.07 2.09 1.02
2.08
1.03
2.22 2.50 1.26 4.94
Cl
N
O
S
O
NH
O
NH
Activate Windows

13CNMR

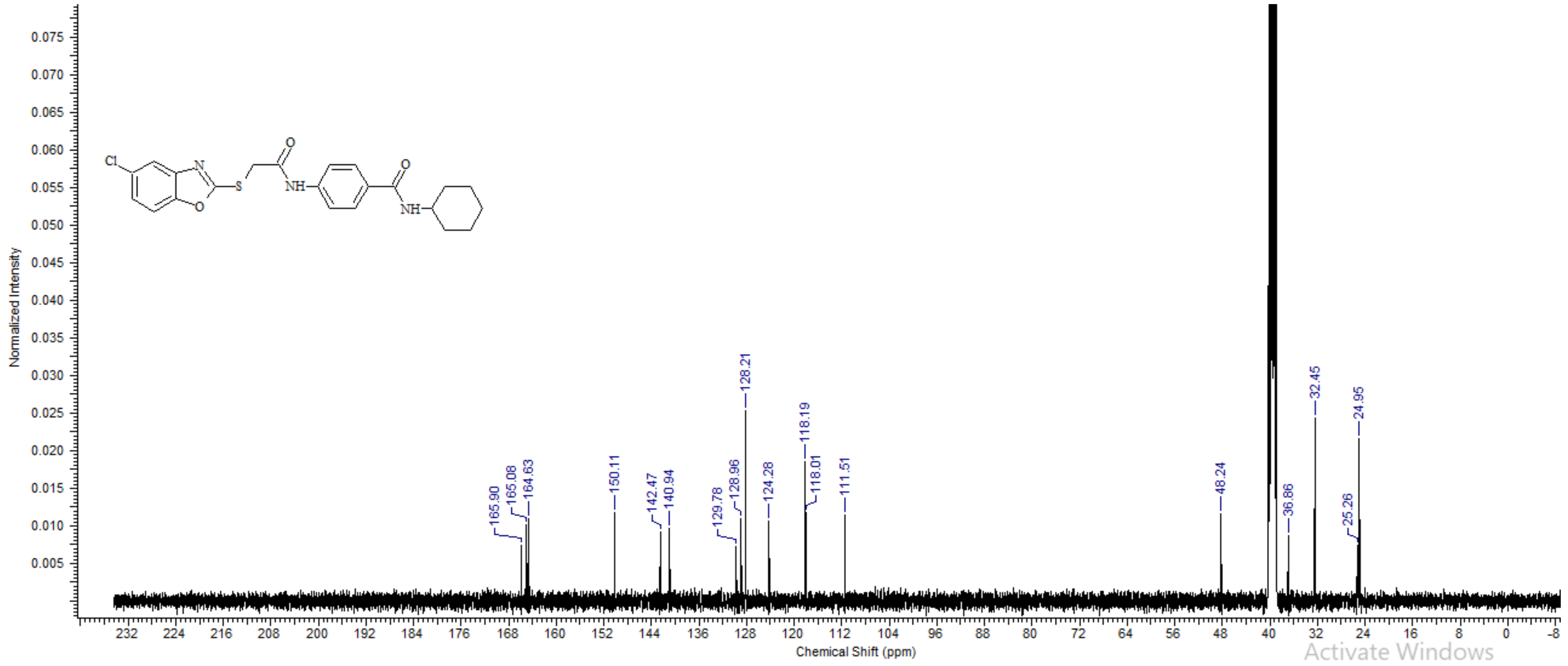

Cl
N
O
S
O
NH
O
NH
Normalized Intensity
0.075
0.070
0.065
0.060
0.055
0.050
0.045
0.040
0.035
0.030
0.025
0.020
0.015
0.010
0.005
165.90
165.08
164.63
150.11
142.47
140.94
129.78
128.96
128.21
124.28
118.19
118.01
111.51
48.24
36.86
32.45
25.26
24.95
232 224 216 208 200 192 184 176 168 160 152 144 136 128 120 112 104 96 88 80 72 64 56 48 40 32 24 16 8 0 -8
Chemical Shift (ppm)
Activate Windows

1.10. 4-(2-(5-chlorobenzo[d]oxazol-2-ylthio)acetamido)-N-phenylbenzamide (10):

Yellow powder (yield 70%); m.p. 245-247°C; IR ($\upsilon_{max.}$/cm^{-1}) 3301 and 3275 (2*N-H), 1672 and 1642 (2*C=O); ^{1}H NMR (400 MHz, DMSO-d_6) δ 10.72 (s, 1H, exchangeable with D_2O, (*$NHCOCH_2S$-)), 10.12 (s, 1H, exchangeable with D_2O), 7.94 (d, *J* = 8.79 Hz, 2H), 7.64 - 7.79 (m, 6H), 7.30 - 7.38 (m, 3H), 7.04 - 7.10 (m, 1H), 4.43 (s, 2H). ^{13}C NMR (101 MHz, DMSO-d_6) δ 165.9, 165.2, 164.8, 150.1, 142.5, 141.5, 139.2, 129.7, 129.0, 128.7, 128.5, 124.3, 123.5, 120.3, 118.4, 118.0, 111.5, 36.9. MS (m/z (R.I. %)): $[M]^+$ 437 (18.2), $[M+2]^+$ 439 (7.8), 345 (34.5), 308 (12.5), 120 (100); Anal. Calcd. for $C_{22}H_{16}ClN_3O_3S$ (437.89); % C, 60.34; H, 3.68; N, 9.60, Found: % C, 60.18; H, 3.82; N, 9.83.

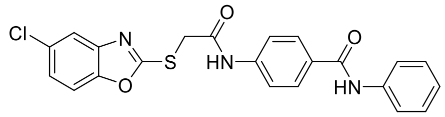

IR

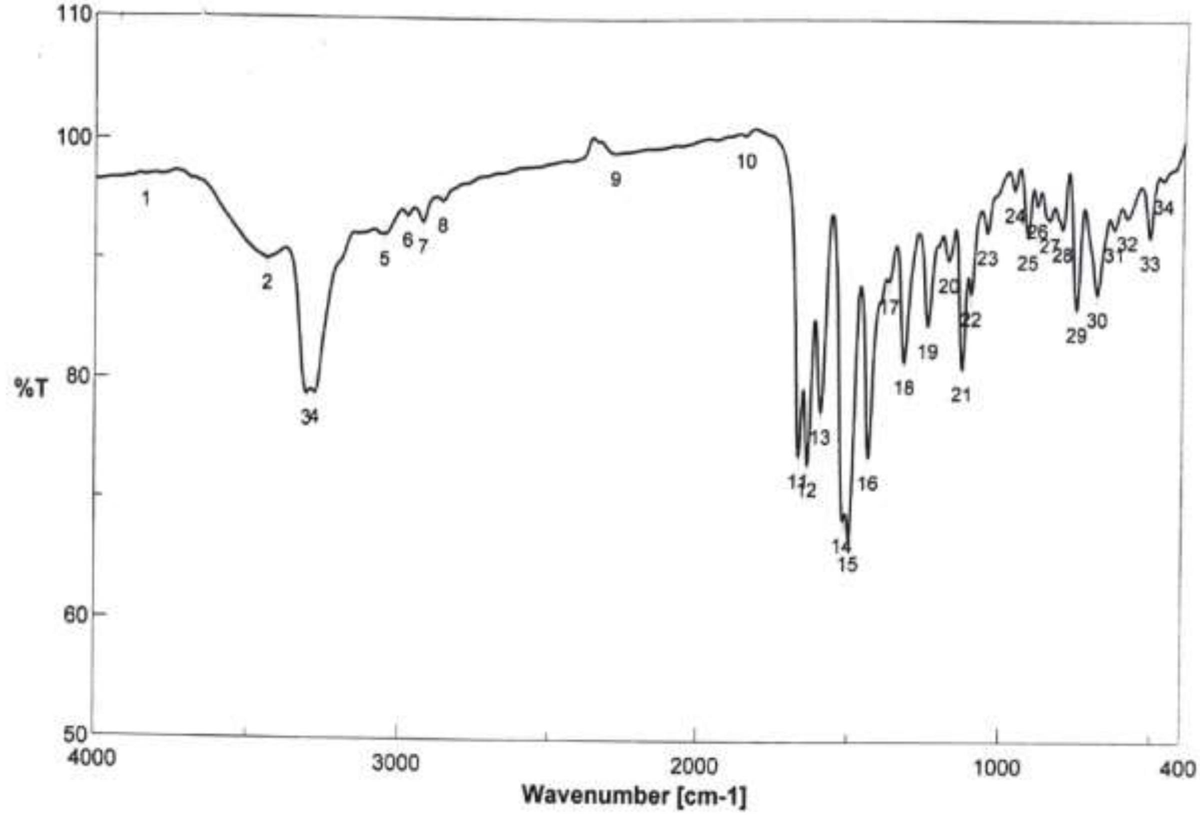

Accumulation	Auto (29)
Resolution	4 cm-1
Zero Filling	ON
Apodization	Cosine
Gain	Auto (2)
Scanning Speed	Auto (2 mm/sec)
Date/Time	5/9/2021 0:18PM
Update	5/9/2021 0:20PM
Operator	IR
File Name	Memory#99
Sample Name	CBA-1
Comment	

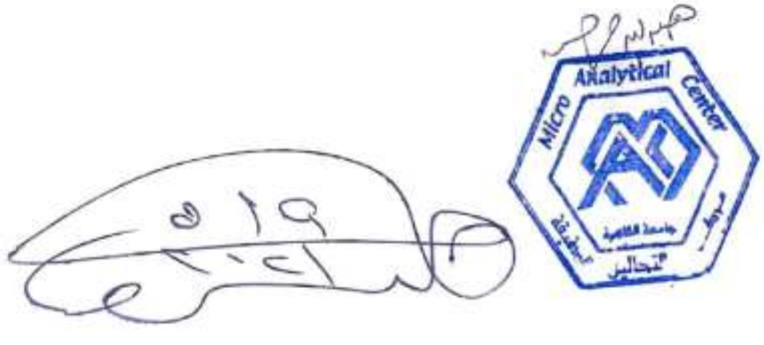

Mass

Cairo University
Micro Analytical Center

DI Analysis
Shimadzu Qp-2010 Plus

Sample Information

Analyzed by	: Dr. Mai Younis
Analyzed	: 01/01/2007 05:20:33 ص
Sample Name	: 1
Sample ID	:
Customer Name	: Dr.Radwan Saeed - Pharmacy - Helwan
Data File	: C:\GCMSsolution\Data\Project1\1.QGD
Org Data File	: C:\GCMSsolution\Data\Project1\1.QGD
Method File	: C:\GCMSsolution\Data\Project1\High Temperature Op
Org Method File	: C:\GCMSsolution\Data\Project1\High Temperature Op
Report File	:
Tuning File	: C:\GCMSsolution\System\Tune1_default.qgt
$EndIf$Modified by	: Dr. Mai Younis
Modified	: 01/01/2007 05:27:53 ص

Method

====== Analytical Line 1 ======

IonSourceTemp	:250.00 °C
[MS Table]	
--Group 1 - Event 1--	
Start Time	:0.00min
End Time	:10.00min
ACQ Mode	:Scan
Event Time	:0.50sec
Scan Speed	:1000
Start m/z	:50.00
End m/z	:500.00
Electron Voltage	: 70 eV
Ionization Mode	: EI

C:\GCMSsolution\Data\Project1\1.QGD

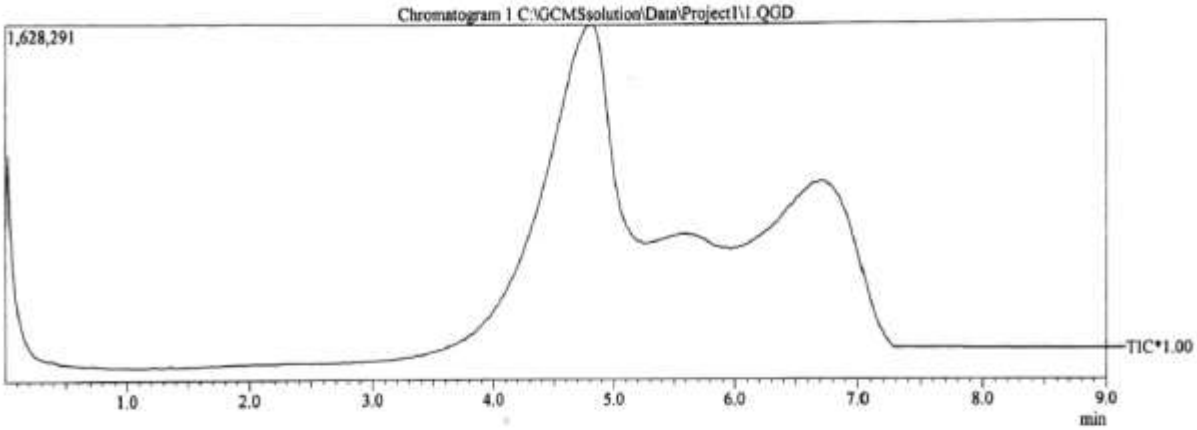

Spectrum

Line#:1 R.Time:4.8(Scan#:575)
MassPeaks:326
RawMode:Single 4.8(575) BasePeak:120(207500)
BG Mode:None Group 1 - Event 1

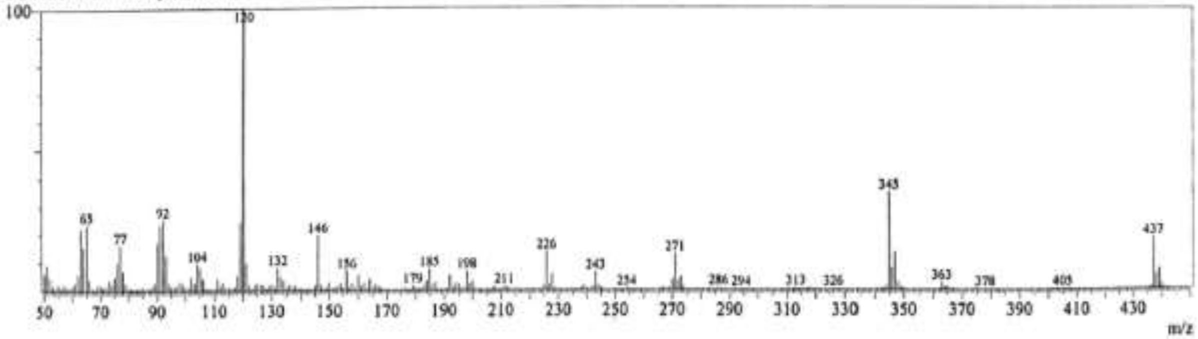

1HNMR

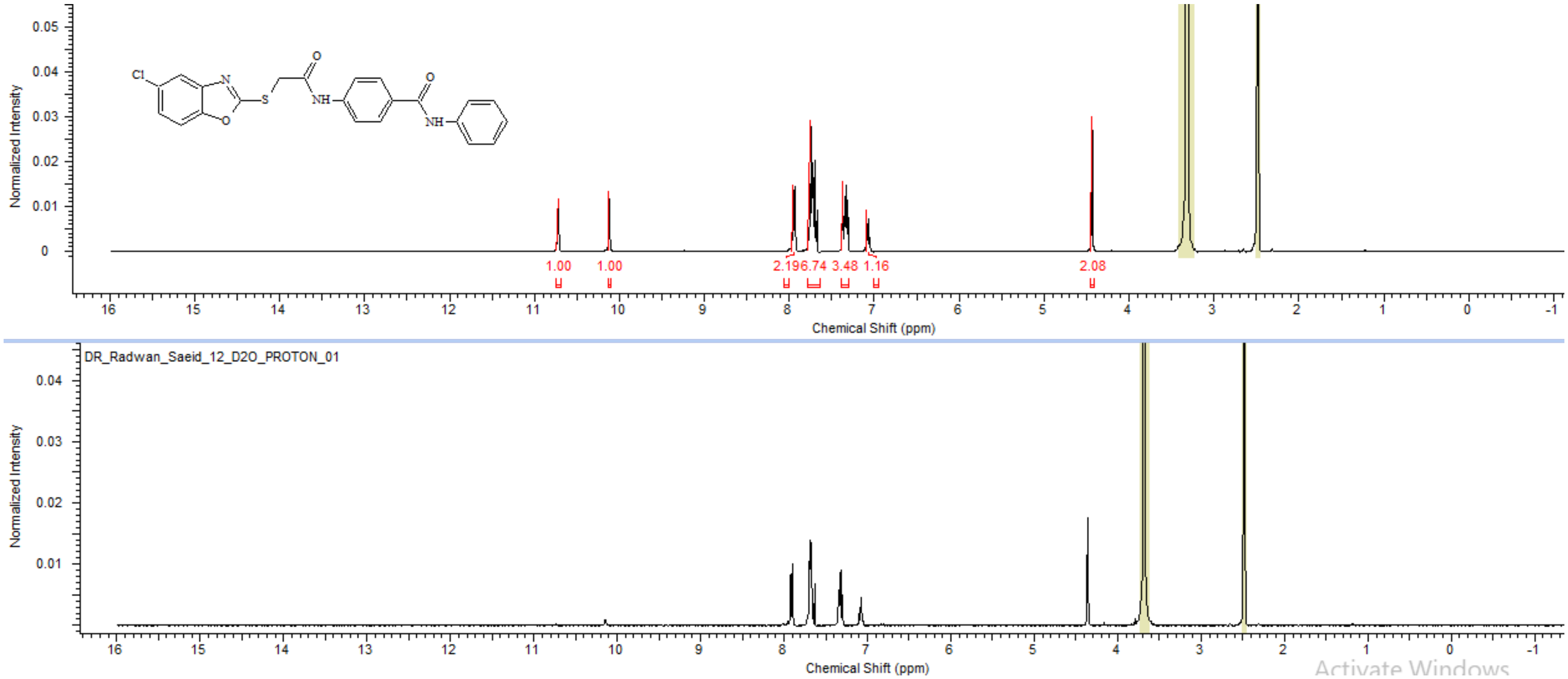

Cl
N
O
S
O
NH
O
NH
Normalized Intensity
1.00
1.00
2.19
6.74
3.48
1.16
2.08
Chemical Shift (ppm)
DR_Radwan_Saeid_12_D2O_PROTON_01
Normalized Intensity
Chemical Shift (ppm)

13CNMR

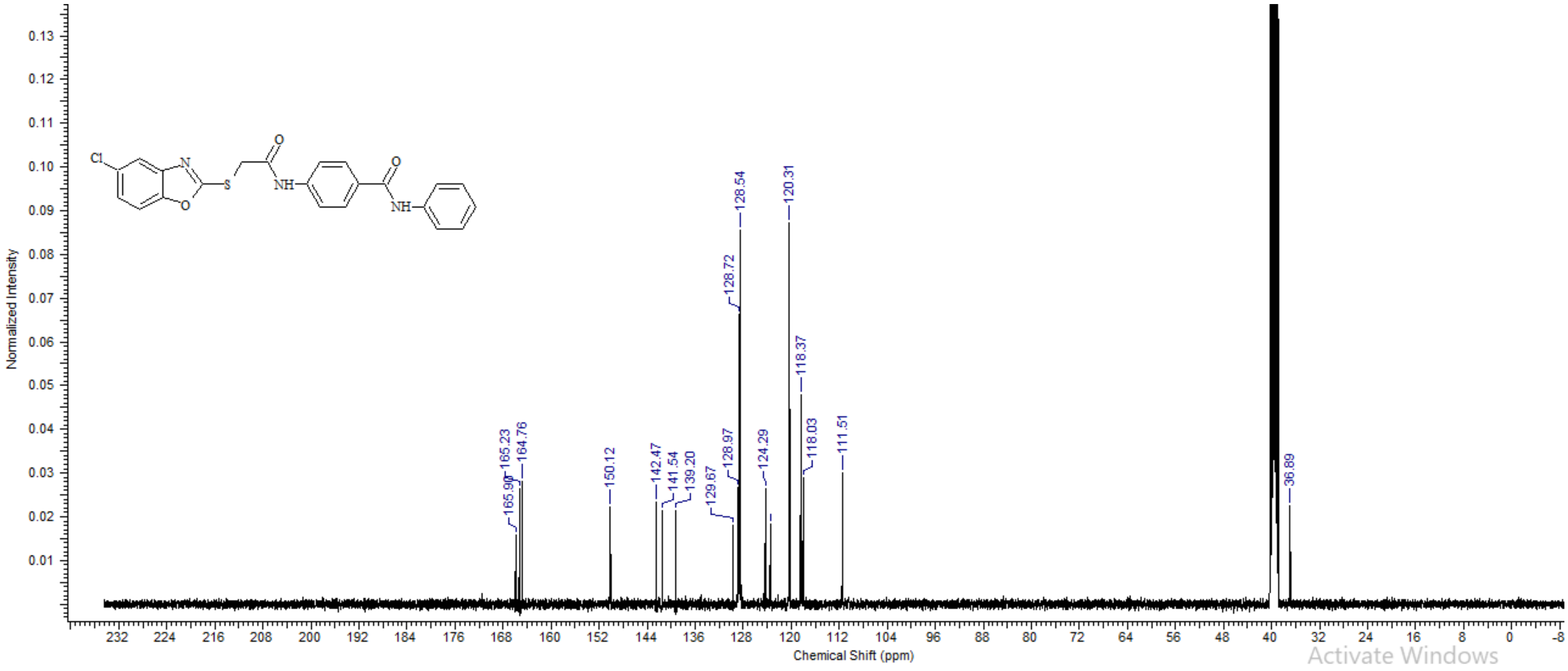
Normalized Intensity
0.13
0.12
0.11
0.10
0.09
0.08
0.07
0.06
0.05
0.04
0.03
0.02
0.01
Cl
N
O
S
O
NH
O
NH
165.90
165.23
164.76
150.12
142.47
141.54
139.20
129.67
128.97
128.72
128.54
124.29
120.31
118.37
118.03
111.51
36.89
232
224
216
208
200
192
184
176
168
160
152
144
136
128
120
112
104
96
88
80
72
64
56
48
40
32
24
16
8
0
-8
Chemical Shift (ppm)
Activate Windows

1.11. 4-(2-(5-chlorobenzo[d]oxazol-2-ylthio)acetamido)-N-(4-chlorophenyl)benzamide (11):

Off-white powder (yield 65%); m.p. 245-247°C; IR ($\upsilon_{max.}$/cm^{-1}) 3300 (N-H), 1661 and 1643 (2*C=O); ^{1}H NMR (400 MHz, DMSO-d$_6$) δ 10.73 (s, 1H, exchangeable with D_2O, (*NHCOCH$_2$S-)), 10.24 (s, 1H, exchangeable with D_2O), 7.94 (d, *J* = 8.79 Hz, 2H), 7.79 (d, *J* = 8.79 Hz, 2H), 7.66 - 7.75 (m, 3H), 7.33 - 7.41 (m, 4H), 4.43 (s, 2H). ^{13}C NMR (101 MHz, DMSO-d$_6$) δ 165.9, 165.3, 164.9, 150.1, 142.5, 141.7, 138.2, 129.4, 129.0, 128.8, 128.5, 127.1, 124.3, 121.8, 118.4, 118.0, 111.5, 36.9. MS (m/z (R.I. %)): [M]$^+$ 471 (9.2), [M+2]$^+$ 473 (7.0), [M+4]$^+$ 475 (1.6), 345 (31.5), 226 (18.6), 120 (100); Anal. Calcd. for $C_{22}H_{15}Cl_2N_3O_3S$ (472.34); % C, 55.94; H, 3.20; N, 8.90, Found: % C, 56.12; H, 3.47; N, 9.14.

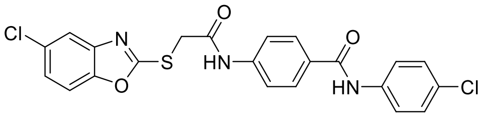

IR

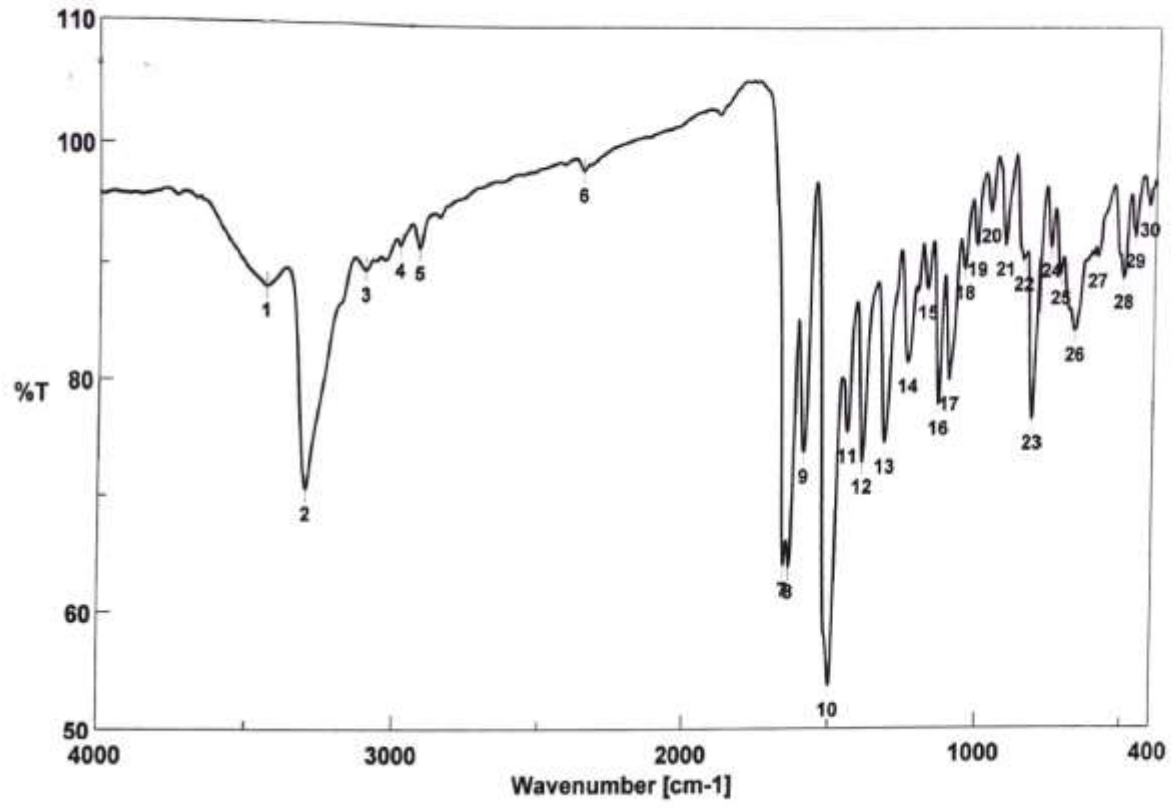

Accumulation	16
Resolution	4 cm-1
Zero Filling	ON
Apodization	Cosine
Gain	Auto (2)
Scanning Speed	Auto (2 mm/sec)
Date/Time	8/22/2021 1:56PM
Update	8/22/2021 1:57PM
Operator	IR
File Name	Memory#104
Sample Name	CBA -3
Comment	

Mass

Cairo University
Micro Analytical Center

DI Analysis
Shimadzu Qp-2010 Plus

Sample Information

Analyzed by	: Dr. Mai Younis
Analyzed	: 01/01/2007 08:31:33 م
Sample Name	: 1
Sample ID	:
Customer Name	: Dr.Radwan Saeed - Pharmacy - Helwan
Data File	: C:\GCMSsolution\Data\Project1\1.QGD
Org Data File	: C:\GCMSsolution\Data\Project1\1.QGD
Method File	: C:\GCMSsolution\Data\Project1\High Temperature Op
Org Method File	: C:\GCMSsolution\Data\Project1\High Temperature Op
Report File	:
Tuning File	: C:\GCMSsolution\System\Tune1_default.qgt
$EndIf$Modified by	: Dr. Mai Younis
Modified	: 01/01/2007 08:37:17 م

Method

------ Analytical Line 1 ------

IonSourceTemp	:250.00 °C
[MS Table]	
--Group 1 - Event 1--	
Start Time	:0.00min
End Time	:10.00min
ACQ Mode	:Scan
Event Time	:0.50sec
Scan Speed	:1250
Start m/z	:50.00
End m/z	:600.00

Electron Voltage	: 70 eV
Ionization Mode	: EI

C:\GCMSsolution\Data\Project1\1.QGD

Chromatogram 1 C:\GCMSsolution\Data\Project1\1.QGD

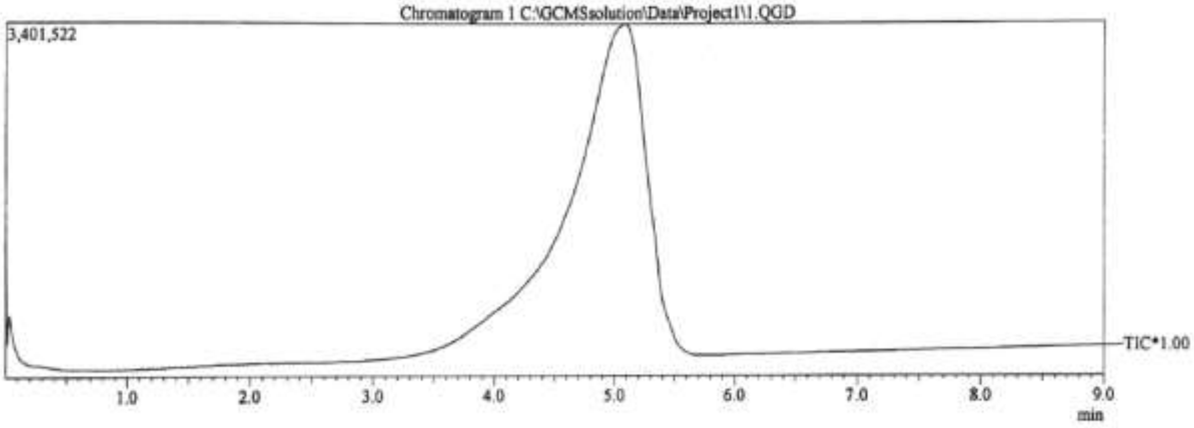

Spectrum

Line#:1 R.Time:5.1(Scan#:612)
MassPeaks:288
RawMode:Single 5.1(612) BasePeak:120(479918)
BG Mode:None Group 1 - Event 1

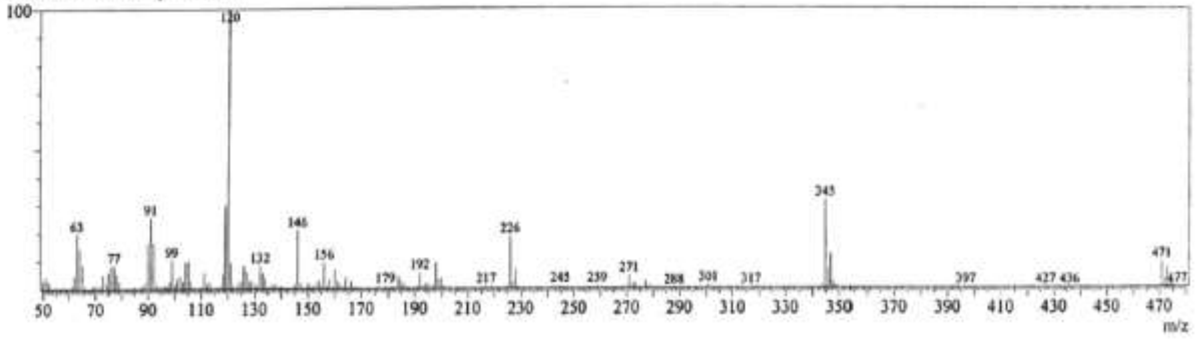

1HNMR

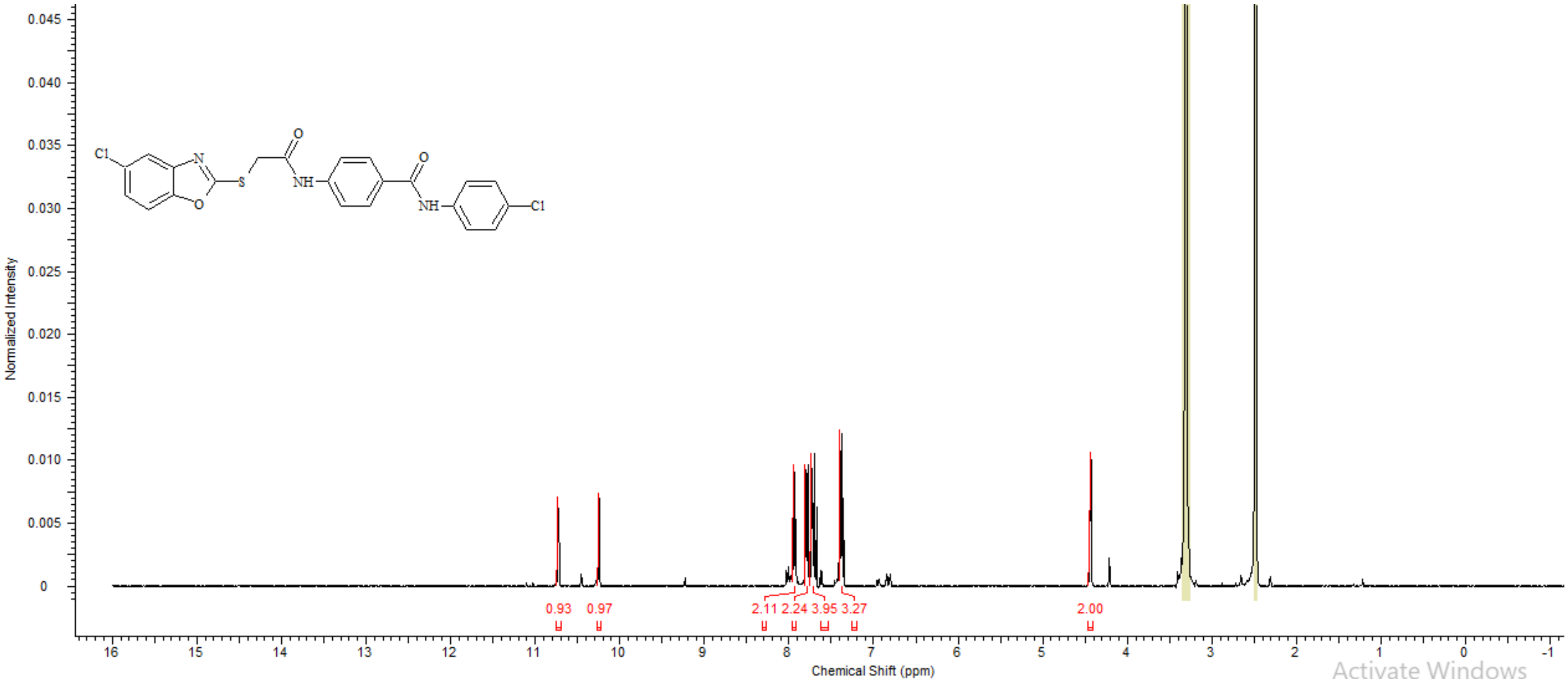

Activate Windows

13CNMR

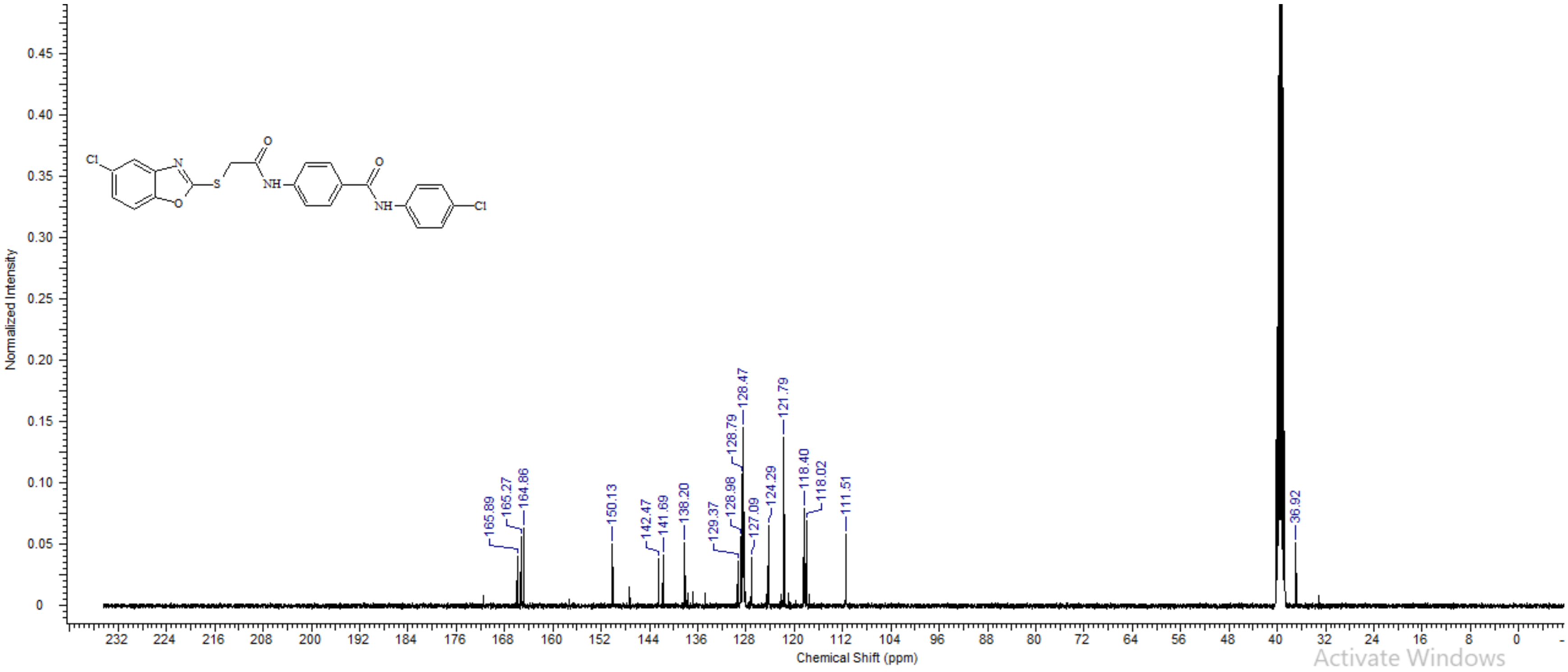

Normalized Intensity
Chemical Shift (ppm)
165.89
165.27
164.86
150.13
142.47
141.69
138.20
129.37
128.98
128.79
128.47
127.09
124.29
121.79
118.40
118.02
111.51
36.92
Cl
N
S
O
NH
O
O
NH
Cl

1.12. 4-(2-(5-chlorobenzo[d]oxazol-2-ylthio)acetamido)-N-(4-methoxyphenyl)benzamide (12):

Yellow powder (yield 78%); m.p. 266-268°C; IR ($\upsilon_{max.}$/cm^{-1}) 3304 (N-H), 1661 and 1637 (2*C=O); ^{1}H NMR (400 MHz, DMSO-d_6) δ 10.70 (s, 1H, exchangeable with D_2O, (*$NHCOCH_2S$-)), 10.00 (s, 1H, exchangeable with D_2O), 7.92 (d, *J* = 8.79 Hz, 2H), 7.73 (d, *J* = 2.20 Hz, 1H), 7.69 (dd, *J* = 3.52, 8.79 Hz, 3H), 7.63 (d, *J* = 9.23 Hz, 2H), 7.36 (dd, *J* = 1.98, 8.57 Hz, 1H), 6.90 (d, *J* = 8.79 Hz, 2H), 4.43 (s, 2H), 3.72 (s, 3H). ^{13}C NMR (101 MHz, DMSO-d_6) δ 165.9, 165.2, 164.3, 155.4, 150.1, 142.5, 141.4, 132.3, 129.8, 129.0, 128.6, 124.3, 121.9, 118.4, 118.0, 113.7, 111.5, 55.2, 36.9. MS (m/z (R.I. %)): [M]$^+$ 467 (57.6), [M+2]$^+$ 469 (23.3), 345 (38.2), 226 (32.8), 120 (100); Anal. Calcd. for $C_{23}H_{18}ClN_3O_4S$ (467.92); % C, 59.04; H, 3.88; N, 8.98, Found: % C, 59.31; H, 4.02; N, 9.25.

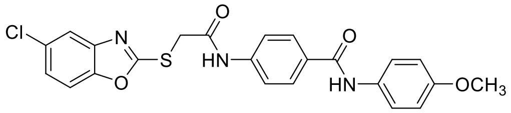

IR

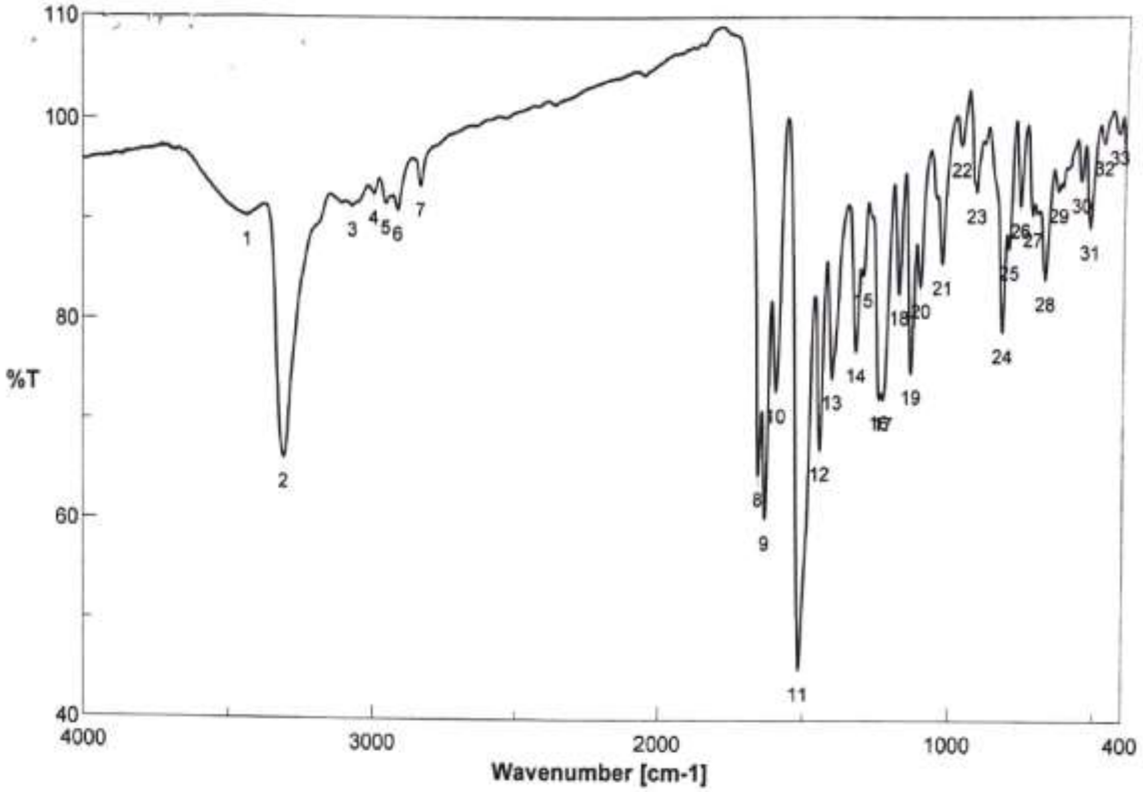

Accumulation Auto (33)
Resolution 4 cm-1
Zero Filling ON
Apodization Cosine
Gain Auto (2)
Scanning Speed Auto (2 mm/sec)
Date/Time 5/9/2021 0:21PM
Update 5/9/2021 0:21PM
Operator IR
File Name Memory#103
Sample Name CBA-19
Comment

Mass

Cairo University
Micro Analytical Center

DI Analysis
Shimadzu Qp-2010 Plus

Dr. Mai Younis

Sample Information

Analyzed by	: Dr. Mai Younis
Analyzed	: 01/01/2007 06:14:43 ص
Sample Name	: 9
Sample ID	:
Customer Name	: Dr.Radwan Saeed - Pharmacy - Helwan
Data File	: C:\GCMSsolution\Data\Project1\9.QGD
Org Data File	: C:\GCMSsolution\Data\Project1\9.QGD
Method File	: C:\GCMSsolution\Data\Project1\High Temperature Op
Org Method File	: C:\GCMSsolution\Data\Project1\High Temperature Op
Report File	:
Tuning File	: C:\GCMSsolution\System\Tune1_default.qgt
$EndIf$Modified by	: Dr. Mai Younis
Modified	: 01/01/2007 06:19:35 ص

Method

===== Analytical Line 1 =====

IonSourceTemp	:250.00 °C
[MS Table]	
--Group 1 - Event 1--	
Start Time	:0.00min
End Time	:10.00min
ACQ Mode	:Scan
Event Time	:0.50sec
Scan Speed	:1000
Start m/z	:50.00
End m/z	:500.00
Electron Voltage	: 70 eV
Ionization Mode	: EI

C:\GCMSsolution\Data\Project1\9.QGD

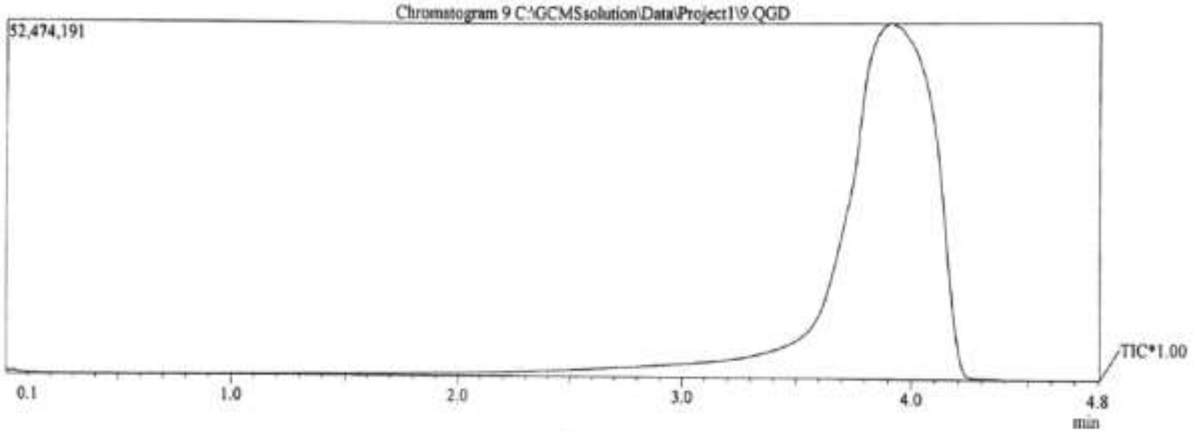

Spectrum

Line#:1 R.Time:3.9(Scan#:467)
MassPeaks:426
RawMode:Single 3.9(467) BasePeak:120(5813115)
BG Mode:None Group 1 - Event 1

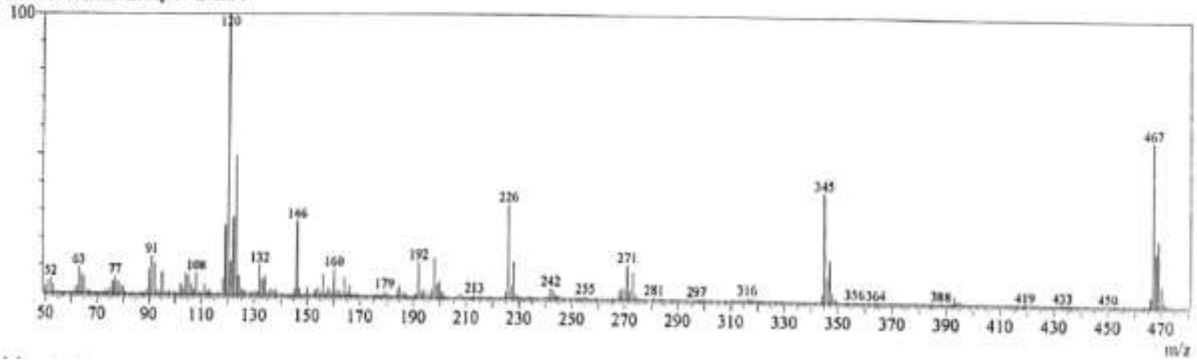

1HNMR

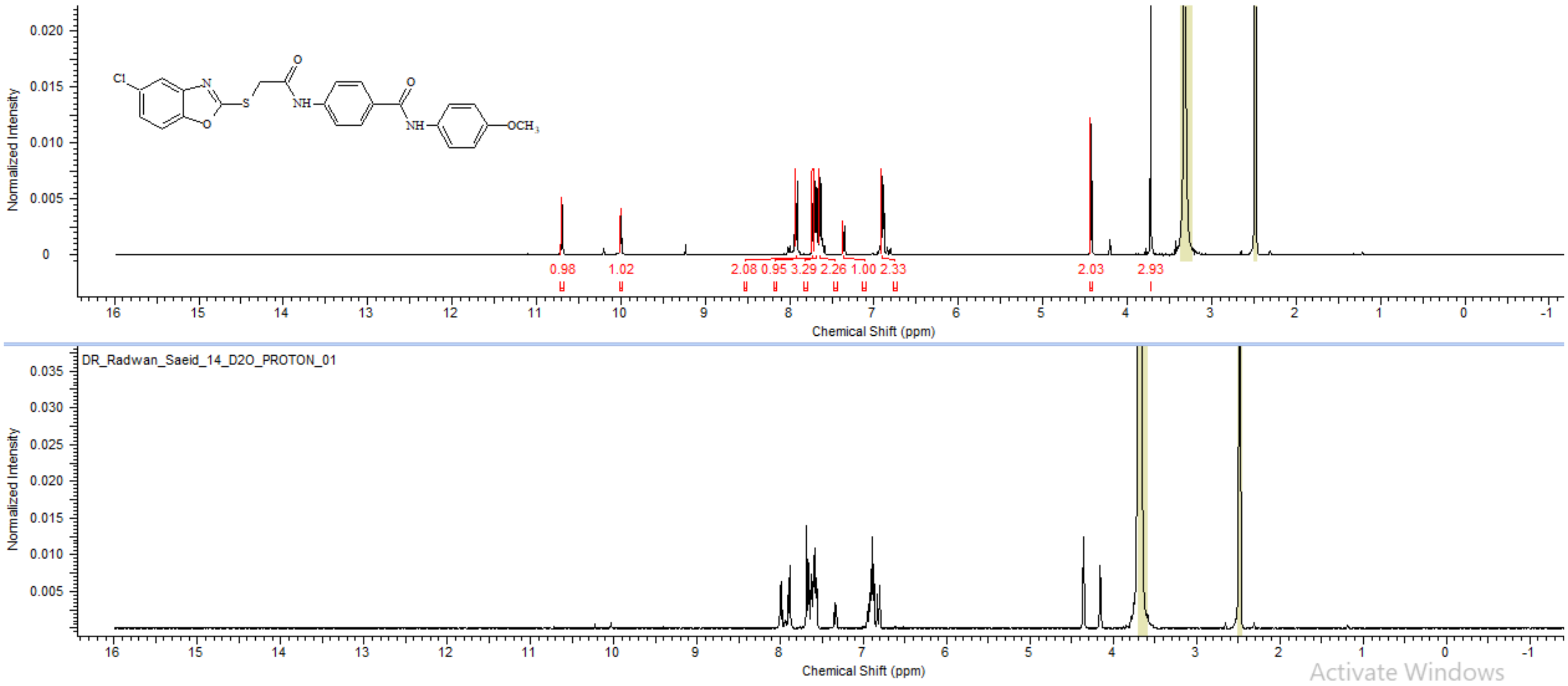

Normalized Intensity
Chemical Shift (ppm)
0.98
1.02
2.08
0.95
3.29
2.26
1.00
2.33
2.03
2.93
DR_Radwan_Saeid_14_D2O_PROTON_01
Activate Windows

13CNMR

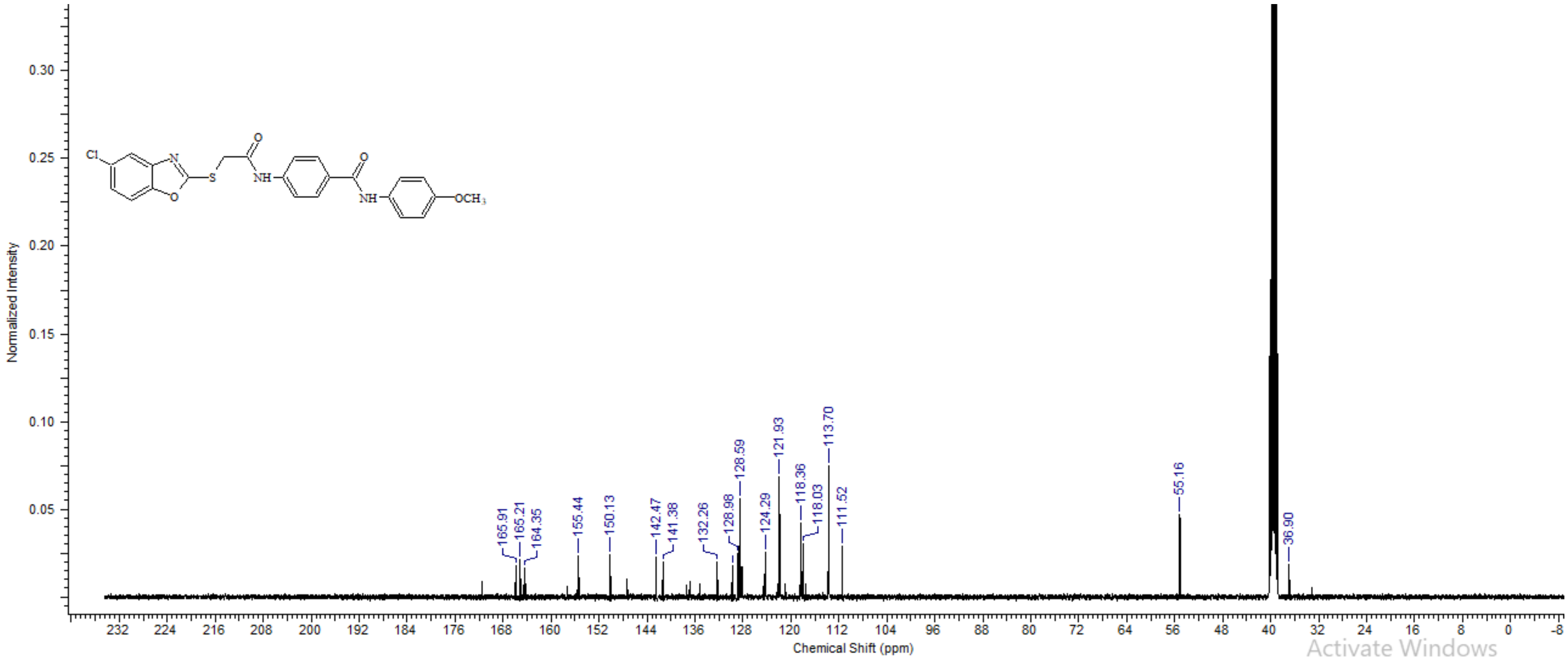

Cl
N
O
S
O
NH
O
NH
OCH3
165.91
165.21
164.35
155.44
150.13
142.47
141.38
132.26
128.98
128.59
124.29
121.93
118.36
118.03
113.70
111.52
55.16
36.90
0.30
0.25
0.20
0.15
0.10
0.05
Normalized Intensity
232
224
216
208
200
192
184
176
168
160
152
144
136
128
120
112
104
96
88
80
72
64
56
48
40
32
24
16
8
0
-8
Chemical Shift (ppm)

1.13. 4-(2-(benzo[d]oxazol-2-ylthio)acetamido)-N'-benzoyl-benzohydrazide (13):

Buff powder (yield 60%); m.p. 270-272 °C; IR ($\upsilon_{max.}$/cm^{-1}) 3290 and 3196 (N-H), 1670 and 1603 (2*C=O); ^{1}H NMR (400 MHz, DMSO-d_6) δ 10.71 (s, 1H,D_2O exchangeable), 10.43 (br. s., 1H, D_2O exchangeable), 10.39 (br. s., 1H, D_2O exchangeable, (*$NHCOCH_2S$-)), 7.90 (d, *J* = 8.35 Hz, 2H), 7.70 (d, *J* = 8.79 Hz, 2H), 7.60 - 7.66 (m, 4H), 7.57 (d, *J* = 7.03 Hz, 1H), 7.51 (d, *J* = 7.91 Hz, 2H), 7.29 - 7.35 (m, 2H), 4.42 (s, 2H). ^{13}C NMR (101 MHz, DMSO-d_6) δ 165.8, 165.5, 165.2, 163.8, 151.3, 141.8, 141.2, 132.6, 131.8, 128.5, 128.5, 127.4, 127.4, 124.7, 124.4, 118.5, 118.2, 110.2, 36.8. MS (m/z (R.I. %)): [M]$^+$ 446 (5.6), 311 (43.2), 192 (34.4), 120 (100), 105 (83.9); Anal. Calcd. for $C_{23}H_{18}N_4O_4S$ (446.48); % C, 61.87; H, 4.06; N, 12.55, Found: % C, 62.04; H, 4.22; N, 12.79.

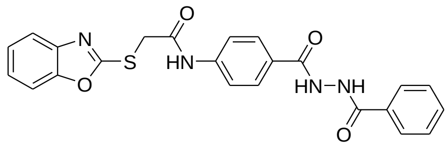

IR

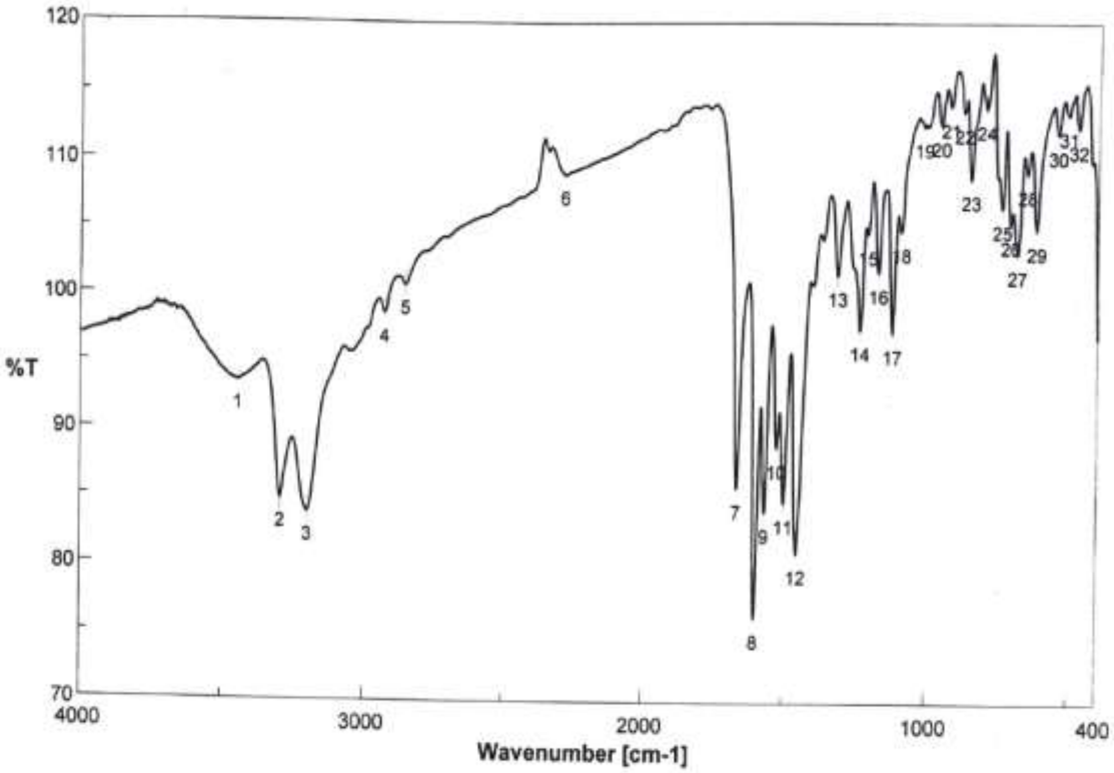

Accumulation	Auto (31)
Resolution	4 cm-1
Zero Filling	ON
Apodization	Cosine
Gain	Auto (2)
Scanning Speed	Auto (2 mm/sec)
Date/Time	5/9/2021 0:06PM
Update	5/9/2021 0:07PM
Operator	IR
File Name	Memory#80
Sample Name	PBA-52
Comment	

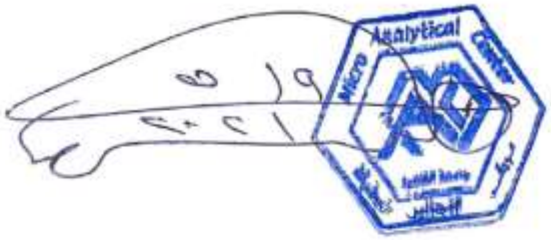

Mass

Cairo University
Micro Analytical Center

DI Analysis
Shimadzu Qp-2010 Plus

Sample Information

Analyzed by	: Dr. Mai Younis
Analyzed	: 01/01/2007 06:34:19 ص
Sample Name	: 12
Sample ID	:
Customer Name	: Dr.Radwan Saeed - Pharmacy - Helwan
Data File	: C:\GCMSsolution\Data\Project1\12.QGD
Org Data File	: C:\GCMSsolution\Data\Project1\12.QGD
Method File	: C:\GCMSsolution\Data\Project1\High Temperature Op
Org Method File	: C:\GCMSsolution\Data\Project1\High Temperature Op
Report File	:
Tuning File	: C:\GCMSsolution\System\Tune1_default.qgt
$EndIf$Modified by	: Dr. Mai Younis
Modified	: 01/01/2007 06:38:53 ص

Method

====== Analytical Line 1 ======

IonSourceTemp	:250.00 °C
[MS Table]	
--Group 1 - Event 1--	
Start Time	:0.00min
End Time	:10.00min
ACQ Mode	:Scan
Event Time	:0.50sec
Scan Speed	:1000
Start m/z	:50.00
End m/z	:500.00
Electron Voltage	: 70 eV
Ionization Mode	: EI

C:\GCMSsolution\Data\Project1\12.QGD

Chromatogram 12 C:\GCMSsolution\Data\Project1\12.QGD

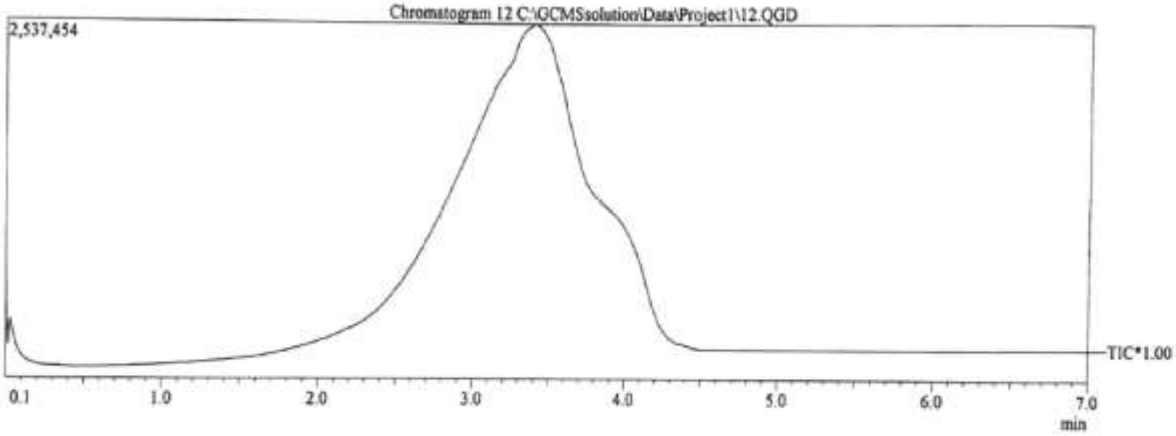

Spectrum

Line#:1 R.Time:3.4(Scan#:408)
MassPeaks:297
RawMode:Single 3.4(408) BasePeak:120(350268)
BG Mode:None Group 1 - Event 1

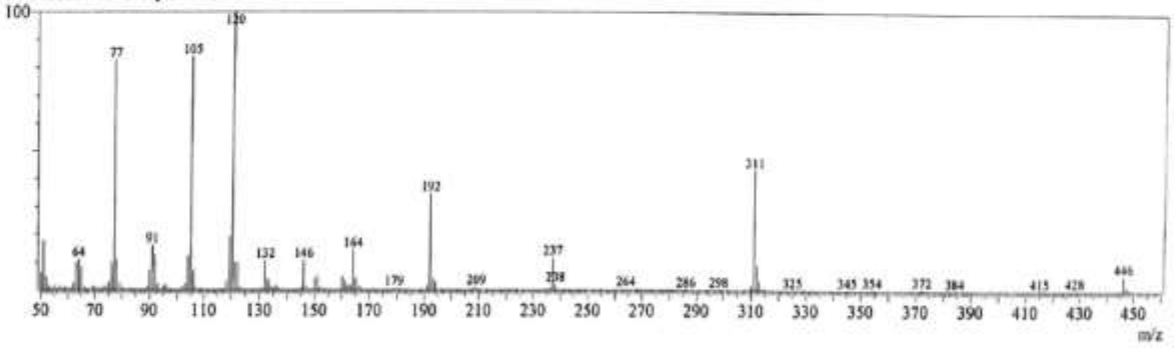

1HNMR

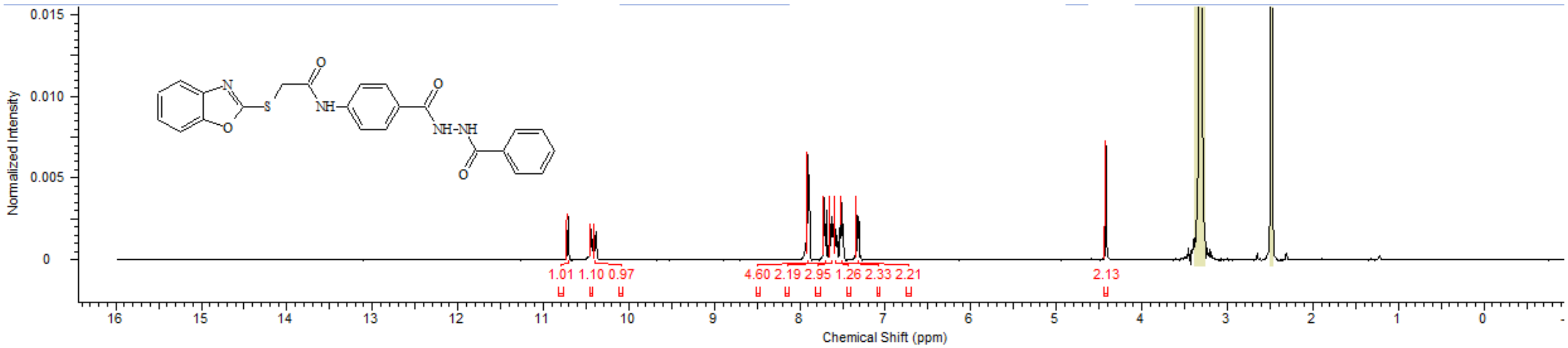
Normalized Intensity
Chemical Shift (ppm)
1.01 1.10 0.97
4.60 2.19 2.95 1.26 2.33 2.21
2.13
NH
NH-NH

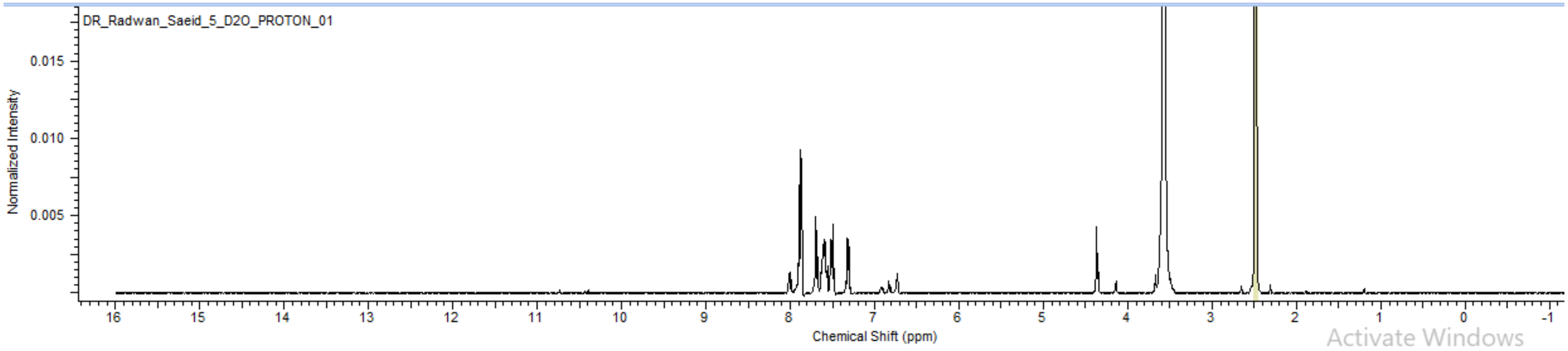
DR_Radwan_Saeid_5_D2O_PROTON_01
Normalized Intensity
Chemical Shift (ppm)
Activate Windows

13CNMR

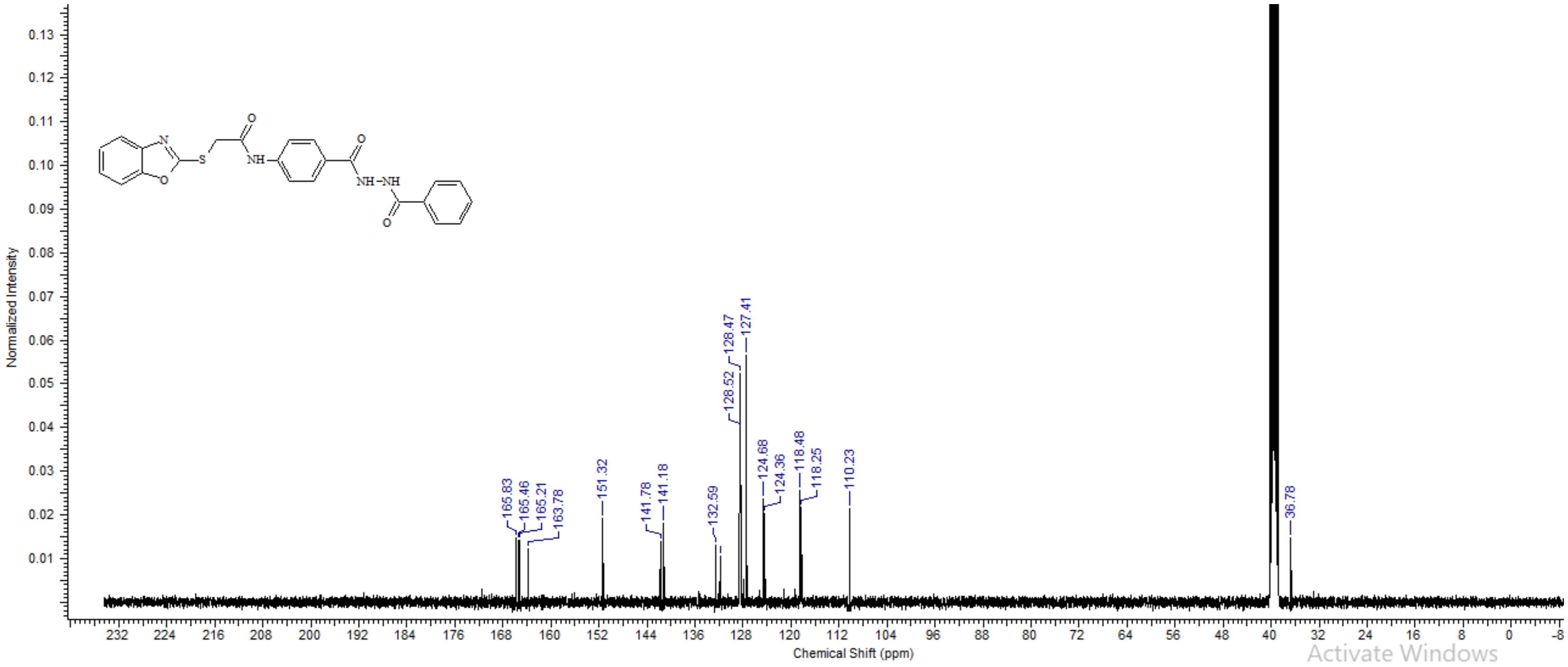
Normalized Intensity
Chemical Shift (ppm)
165.83
165.46
165.21
163.78
151.32
141.78
141.18
132.59
128.52
128.47
127.41
124.68
124.36
118.48
118.25
110.23
36.78
Activate Windows

1.14. 4-(2-(5-methylbenzo[d]oxazol-2-ylthio)acetamido)-N'-benzoyl-benzohydrazide (14):

Buff powder (yield 80%); m.p. 250-252 ^{o}C; IR ($\upsilon_{max.}$/cm^{-1}) 3292 and 3184 (N-H), 1661 and 1601 (2*C=O); ^{1}H NMR (400 MHz, DMSO-d$_6$) δ 10.70 (s, 1H, D_2O exchangeable), 10.44 (s, 1H, D_2O exchangeable), 10.39 (s, 1H,D_2O exchangeable, (*NHCOCH$_2$S-)), 7.86 - 7.93 (m, 4H), 7.70 (d, *J* = 8.79 Hz, 2H), 7.57 (d, *J* = 7.47 Hz, 1H), 7.51 (dd, *J* = 5.27, 7.91 Hz, 3H), 7.41 (s, 1H), 7.12 (d, *J* = 8.35 Hz, 1H), 4.40 (s, 2H), 2.38 (s, 3H). ^{13}C NMR (101 MHz, DMSO-d$_6$) δ 165.9, 165.5, 165.3, 163.7, 149.6, 141.8, 141.4, 134.1, 132.6, 128.5, 127.4, 125.2, 122.5, 120.9, 119.4, 118.5, 118.2, 109.6, 36.8, 20.9. MS (m/z (R.I. %)): [M]$^+$ 460 (6.9), 325 (35.5), 206 (28.9), 120 (100), 105 (85.0); Anal. Calcd. for $C_{24}H_{20}N_4O_4S$ (460.50); % C, 62.60; H, 4.38; N, 12.17, Found: % C, 62.43; H, 4.60; N, 12.45.

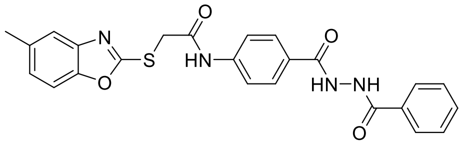

IR

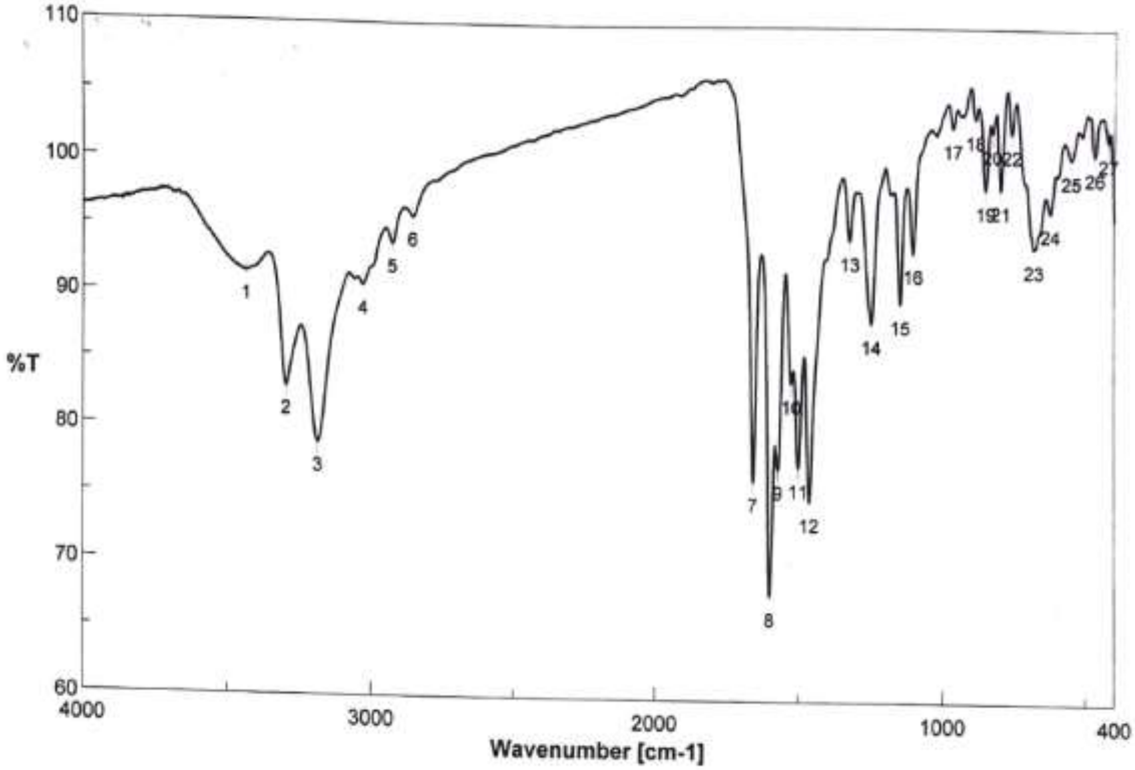

Accumulation	Auto (29)
Resolution	4 cm-1
Zero Filling	ON
Apodization	Cosine
Gain	Auto (2)
Scanning Speed	Auto (2 mm/sec)
Date/Time	5/9/2021 11:57AM
Update	5/9/2021 0:17PM
Operator	IR
File Name	Memory#61
Sample Name	MBA-52
Comment	

Mass

Cairo University
Micro Analytical Center

DI Analysis
Shimadzu Qp-2010 Plus

Sample Information

Analyzed by	: Dr. Mai Younis
Analyzed	: 01/01/2007 06:28:18 ص
Sample Name	: 11
Sample ID	:
Customer Name	: Dr.Radwan Saeed - Pharmacy - Helwan
Data File	: C:\GCMSsolution\Data\Project1\11.QGD
Org Data File	: C:\GCMSsolution\Data\Project1\11.QGD
Method File	: C:\GCMSsolution\Data\Project1\High Temperature Op
Org Method File	: C:\GCMSsolution\Data\Project1\High Temperature Op
Report File	:
Tuning File	: C:\GCMSsolution\System\Tune1_default.qgt
$EndIf$Modified by	: Dr. Mai Younis
Modified	: 01/01/2007 06:32:22 ص

Method

====== Analytical Line 1 ======

IonSourceTemp	:250.00 °C
[MS Table]	
--Group 1 - Event 1--	
Start Time	:0.00min
End Time	:10.00min
ACQ Mode	:Scan
Event Time	:0.50sec
Scan Speed	:1000
Start m/z	:50.00
End m/z	:500.00
Electron Voltage	: 70 eV
Ionization Mode	: EI

C:\GCMSsolution\Data\Project1\11.QGD

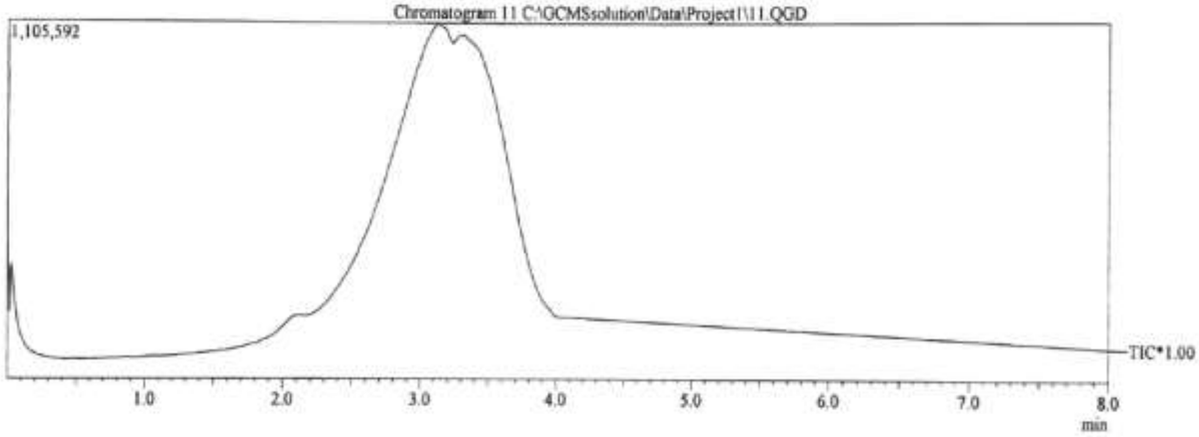

Spectrum

Line#:1 R.Time:3.3(Scan#:392)
MassPeaks:274
RawMode:Single 3.3(392) BasePeak:120(143630)
BG Mode:None Group 1 - Event 1

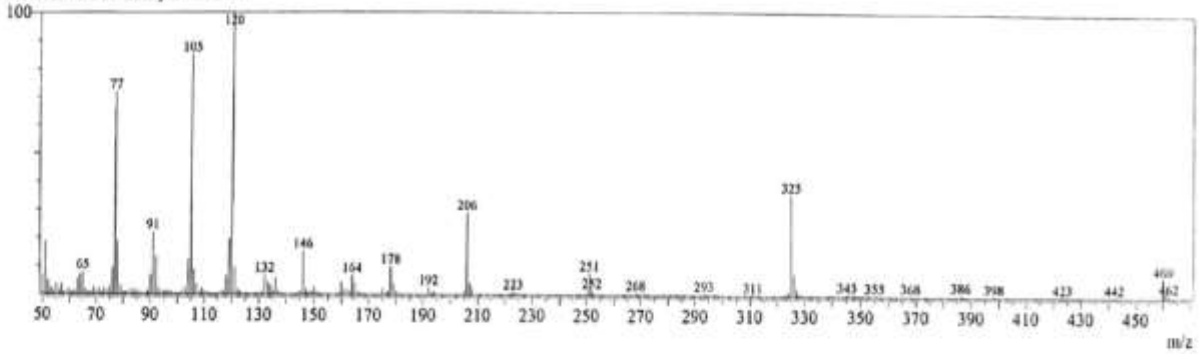

1HNMR

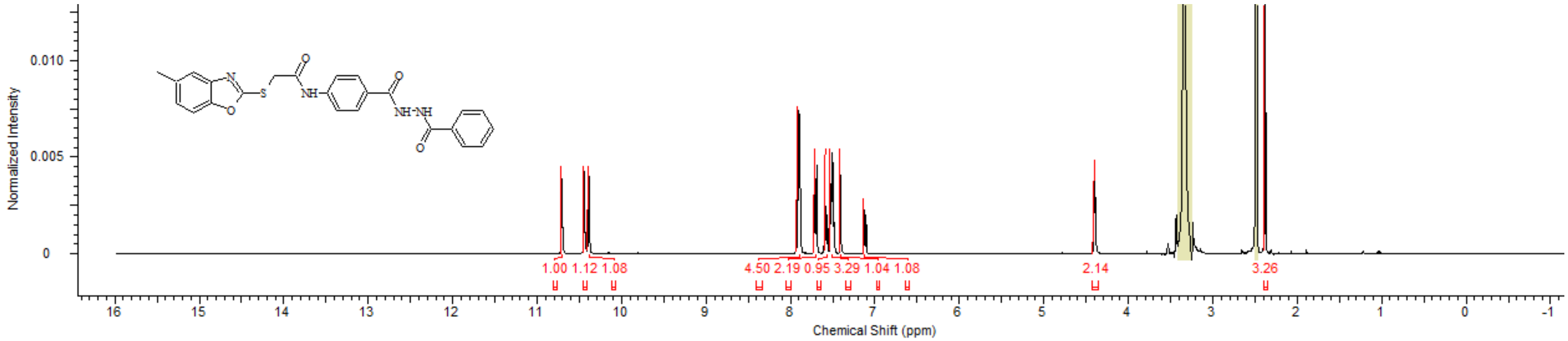

Normalized Intensity
0.010
0.005
0
1.00 1.12 1.08
4.50 2.19 0.95 3.29 1.04 1.08
2.14
3.26
16 15 14 13 12 11 10 9 8 7 6 5 4 3 2 1 0 -1
Chemical Shift (ppm)

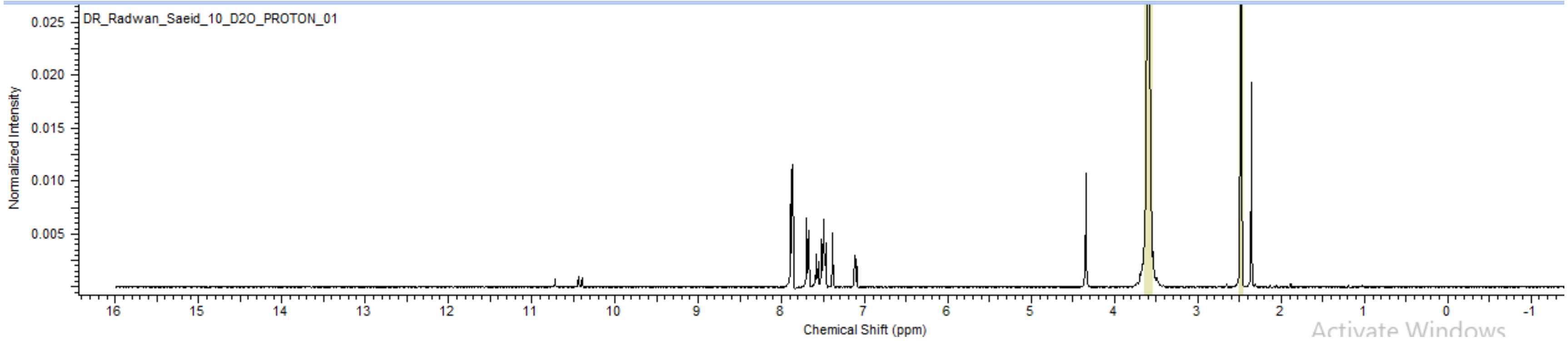

DR_Radwan_Saeid_10_D2O_PROTON_01
Normalized Intensity
0.025
0.020
0.015
0.010
0.005
16 15 14 13 12 11 10 9 8 7 6 5 4 3 2 1 0 -1
Chemical Shift (ppm)
Activate Windows

13CNMR

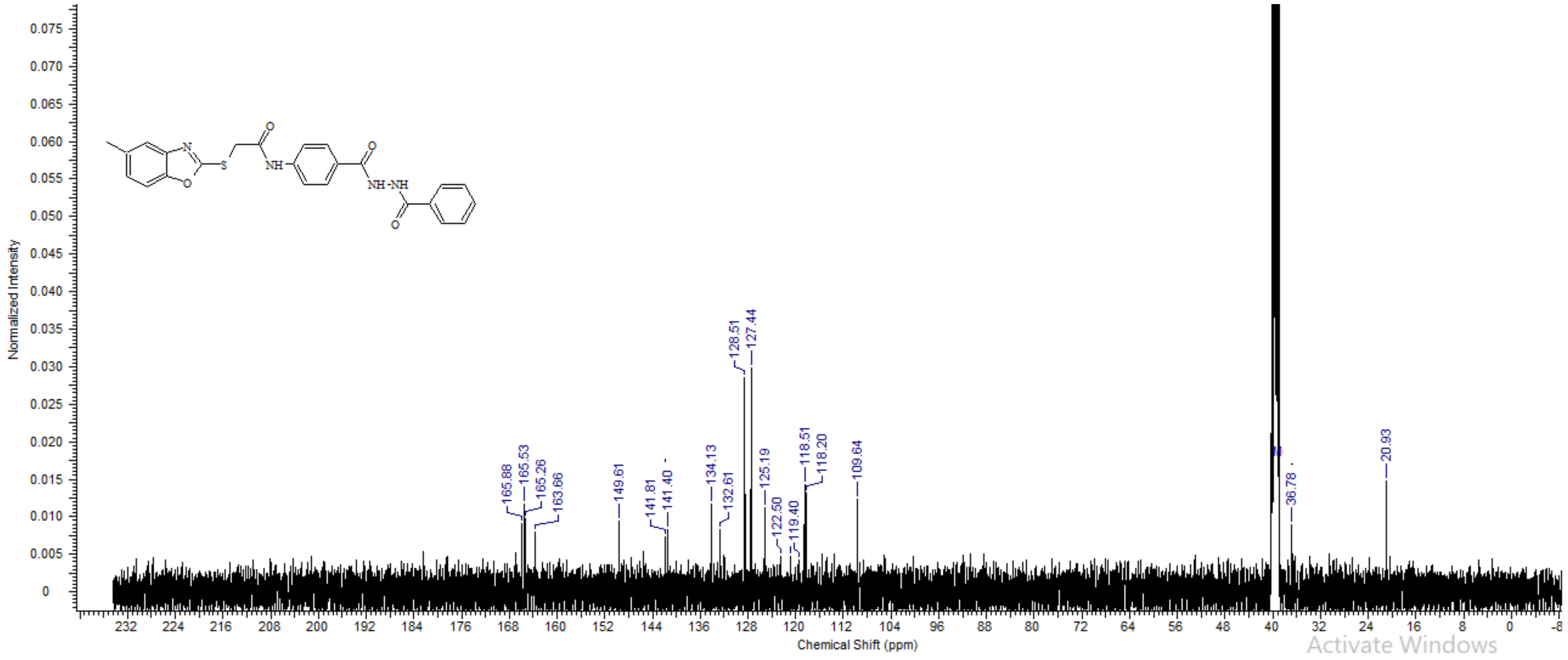

Normalized Intensity
Chemical Shift (ppm)
165.88
165.53
165.26
163.66
149.61
141.81
141.40
134.13
132.61
128.51
127.44
125.19
122.50
119.40
118.51
118.20
109.64
36.78
20.93
NH
NH-NH
Activate Windows

1.15. 4-(2-(5-chlorobenzo[d]oxazol-2-ylthio)acetamido)-N'-benzoyl-benzohydrazide (15):

Off-white powder (yield 62%); m.p. 277-279ºC; IR ($\upsilon_{max.}$/cm^{-1}) 3290 and 3184 (2*N-H), 1660 and 1601 (2*C=O); ^{1}H NMR (400 MHz, DMSO-d$_6$) δ 10.72 (s, 1H, D_2O exchangeable), 10.44 (s, 1H, D_2O exchangeable), 10.39 (s, 1H, D_2O exchangeable, (*NHCOCH$_2$S-)), 7.90 (d, *J* = 8.79 Hz, 4H), 7.73 (d, *J* = 2.20 Hz, 1H), 7.69 (dd, *J* = 5.49, 8.57 Hz, 3H), 7.55 – 7.61 (m, 1H), 7.48 - 7.53 (m, 2H), 7.36 (dd, *J* = 1.98, 8.57 Hz, 1H), 4.43 (s, 2H). MS (m/z (R.I. %)): [M]$^+$ 480 (1.9), [M+2]$^+$ 482 (0.8), 345 (18.3), 271 (20.6), 120 (72.1), 105 (100); Anal. Calcd. For $C_{23}H_{17}ClN_4O_4S$ (480.92); % C, 57.44; H, 3.56; N, 11.65, Found: % C, 57.71; H, 3.70; N, 11.49.

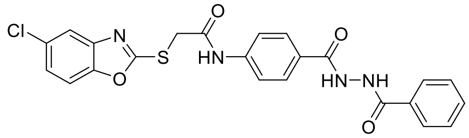

IR

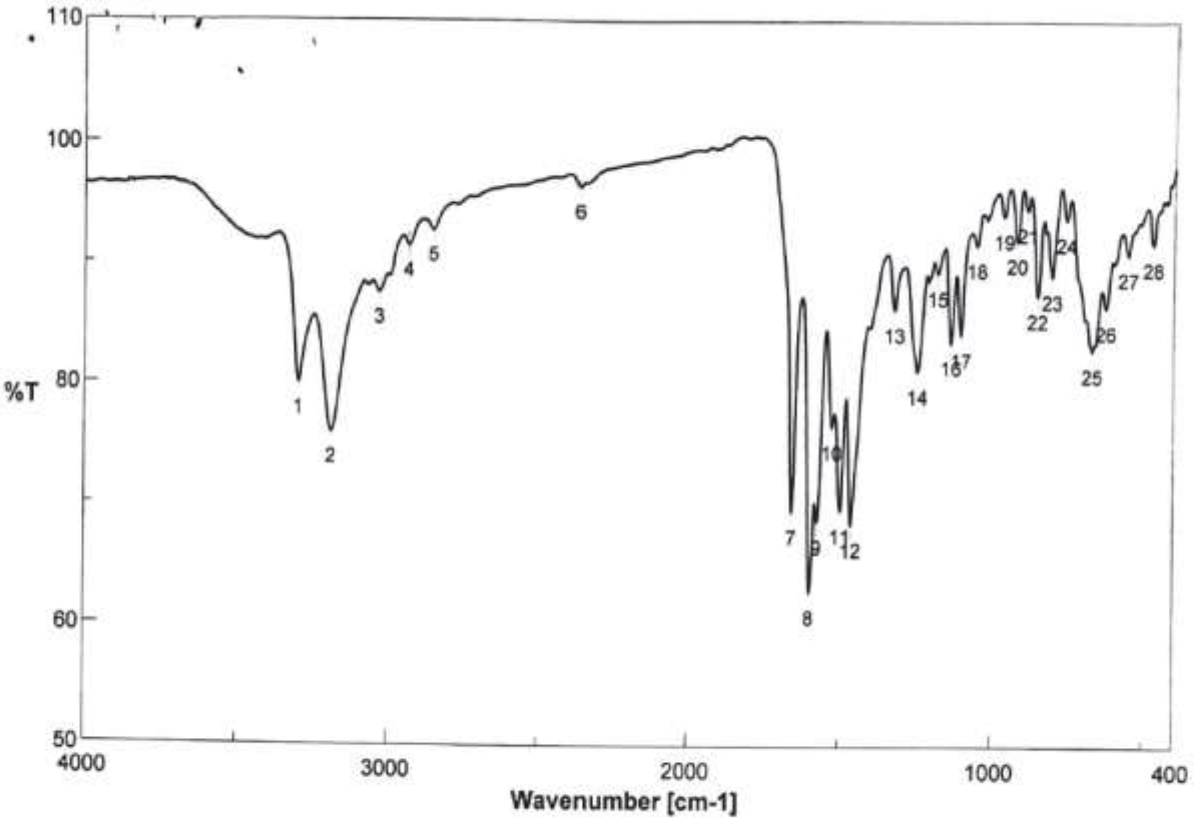

Accumulation	Auto (29)
Resolution	4 cm-1
Zero Filling	ON
Apodization	Cosine
Gain	Auto (2)
Scanning Speed	Auto (2 mm/sec)
Date/Time	5/9/2021 0:12PM
Update	5/9/2021 0:12PM
Operator	IR
File Name	Memory#92
Sample Name	CBA-52
Comment	

Mass

Cairo University
Micro Analytical Center

DI Analysis
Shimadzu Qp-2010 Plus

Sample Information

Analyzed by	: Dr. Mai Younis
Analyzed	: 01/01/2007 06:21:36 ص
Sample Name	: 10
Sample ID	:
Customer Name	: Dr.Radwan Saeed - Pharmacy - Helwan
Data File	: C:\GCMSsolution\Data\Project1\10.QGD
Org Data File	: C:\GCMSsolution\Data\Project1\10.QGD
Method File	: C:\GCMSsolution\Data\Project1\High Temperature Op
Org Method File	: C:\GCMSsolution\Data\Project1\High Temperature Op
Report File	:
Tuning File	: C:\GCMSsolution\System\Tune1_default.qgt
$EndIf$Modified by	: Dr. Mai Younis
Modified	: 01/01/2007 06:26:08 ص

Method

====== Analytical Line 1 ======

IonSourceTemp	:250.00 °C
[MS Table]	
--Group 1 - Event 1--	
Start Time	:0.00min
End Time	:10.00min
ACQ Mode	:Scan
Event Time	:0.50sec
Scan Speed	:1000
Start m/z	:50.00
End m/z	:500.00
Electron Voltage	: 70 eV
Ionization Mode	: EI

C:\GCMSsolution\Data\Project1\10.QGD

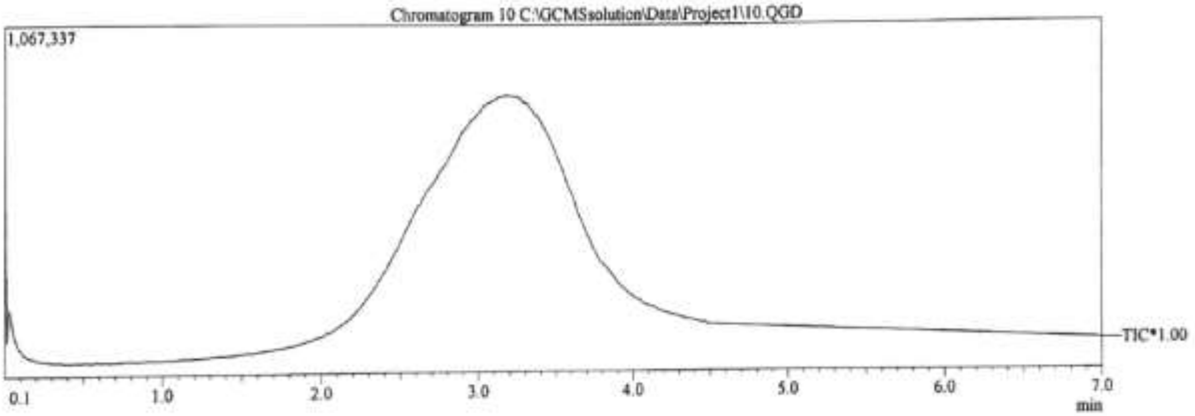

Spectrum

Line#:1 R.Time:3.4(Scan#:407)
MassPeaks:307
RawMode:Single 3.4(407) BasePeak:105(101713)
BG Mode:None Group 1 - Event 1

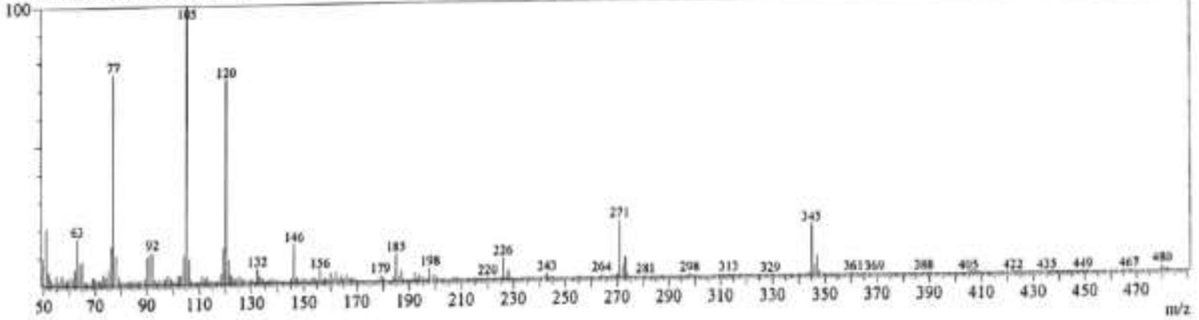

1HNMR

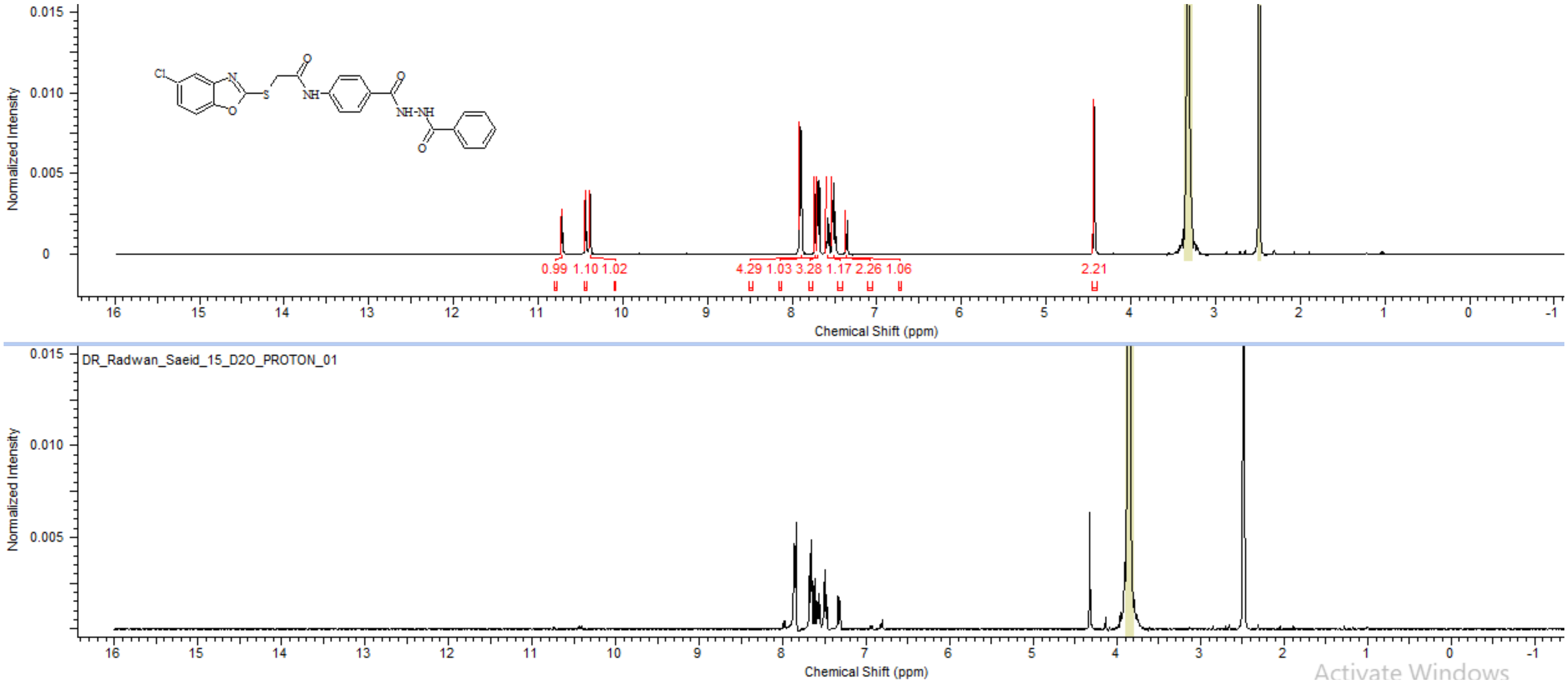
Normalized Intensity
Cl
N
O
S
O
NH
O
NH-NH
O
0.99 1.10 1.02
4.29 1.03 3.28 1.17 2.26 1.06
2.21
Chemical Shift (ppm)
DR_Radwan_Saeid_15_D2O_PROTON_01
Normalized Intensity
Chemical Shift (ppm)

13CNMR

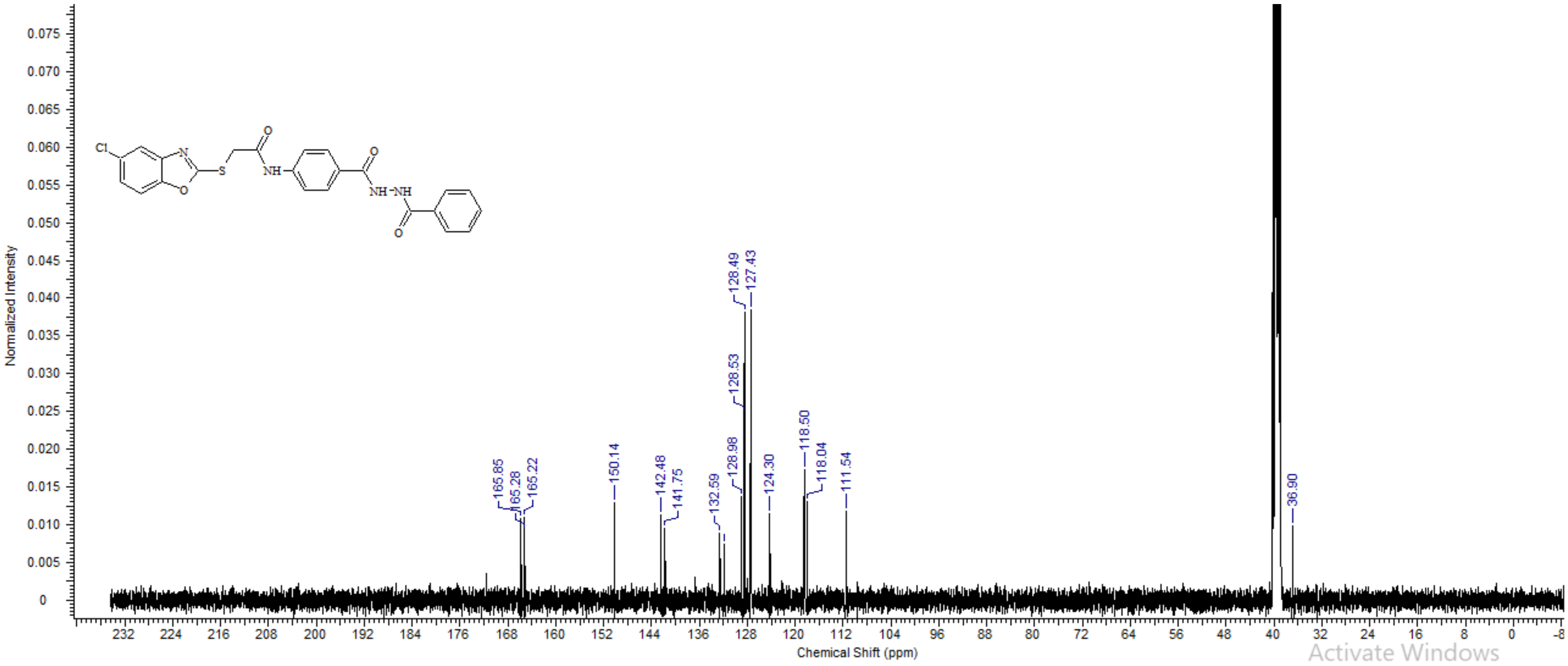

Normalized Intensity
Chemical Shift (ppm)
165.85
165.28
165.22
150.14
142.48
141.75
132.59
128.98
128.53
128.49
127.43
124.30
118.50
118.04
111.54
36.90
Cl
N
S
O
NH
NH-NH
Activate Windows

Elemental Analysis:

No.	Code
1	PBA 8
2	PBA 1
3	PBA 3
4	PBA 19
13	PBA 52
5	MBA 8
6	MBA 1
7	MBA 3
8	MBA 19
14	MBA 52
9	CBA 8
10	CBA 1
11	CBA 3
12	CBA 19
15	CBA 52

Al-Azhar University

The Regional Center for Mycology and Biotechnology

Requester Data:

Name: Prof. Dr. Radwan El-Haggar

Authority: Faculty of Pharmacy, Helwan University

Sample Data:

Fifteen samples had been submitted for elemental analysis.

Analysis Report:

Sample Code	C%	H%	N%
CBA$_1$	60.18	3.82	9.83
CBA$_3$	56.12	3.47	9.14
CBA$_8$	59.75	5.11	9.71
CBA$_{19}$	59.31	4.02	9.25
CBA$_{52}$	57.71	3.70	11.49
MBA$_1$	65.97	4.86	10.39
MBA$_3$	61.40	4.25	9.18
MBA$_8$	64.97	5.86	10.21
MBA$_{19}$	64.65	4.98	9.61
MBA$_{52}$	62.43	4.60	12.45
PBA$_1$	65.31	5.89	10.44
PBA$_3$	60.47	3.80	9.86
PBA$_8$	64.31	5.89	10.45
PBA$_{19}$	63.90	4.63	9.95
PBA$_{52}$	62.04	4.22	12.79

INVESTIGATOR DIRECTOR

Al-Azhar University Campus - Nasr City, Cairo, Egypt.
Tel: 0202 22620373 Fax : 0202 22620373
E.mail:rcmb@azhar.edu.eg
Website: http://www.azhar.edu.eg.htm * http://www.azhar.edu.eg/pages/fungi_center.htm
Facebook : RCMB AZHAR P.O. box mail : 11751 Nasr City Cairo, Egypt.

2. Biological Evaluation

2.1. Anti-proliferative activity against HCT-116 and MCF-7 human cancer cells lines.

The sulforhodamine B (SRB) assays were performed according to Skehan et al. [55]. Briefly, exponentially growing cells were trypsinized, counted and seeded at the appropriate densities (5000 cells/100 μL/ well) into 96-well microtiter plates. Cells were incubated in a humidified atmosphere at 37 °C for 24 h. Then, the cells were exposed to the tested compounds at the desired concentrations (0.01, 0.1, 1, 10, and 100 μM) or to 1% dimethyl sulfoxide (DMSO) for 72 h. At the end of the treatment period, the media were removed, and the cells were fixed with 10% trichloroacetic acid at 4 °C for 1 h. Following, the cells were washed with tap water four times and incubated with SRB 0.4% for 30 min. Excess dye was removed by washing repeatedly with 1% (vol/vol) acetic acid. The protein-bound dye was dissolved in 10 mM Tris base solution for (optical density) OD determination at 510 nm using a Spectra Max plus Microplate Reader (Molecular Devices, CA). Cell viability was expressed relative to the untreated control cells [62].

2.2. VEGFR-2 Inhibitory activity

IC_{50}s of compounds **1** and **11** were evaluated *in vitro* using colorimetric assay of human VEGFR-2 ELISA (Enzyme-Linked Immunosorbent Assay) kits (HTScan® VEGF Receptor-2 Kinase Assay Kit). It includes active VEGFR-2 kinase (a biotinylated peptide substrate and a phospho-tyrosine antibody) for detection of the phosphorylated form of the substrate peptide). On a 96-well plate, a particular VEGFR-2 antibody was seeded and 100 μL of the normal solution or the tested compound tested was applied, incubated at room temperature for 2.5 h and washed. Then, 100 μL of the prepared biotin antibody was added, incubated for an additional 1 h at room temperature and washed. Following, 100 μL of streptavidin solution was added at room temperature, incubated for 45 minutes and then, 100 μL of TMB Substrate solution was applied and incubated at room temperature for 30 minutes. Finally, 50 μL stop solution was added and the absorption was measured at 450 nm instantly. The standard curve, the X-axis concentrations, and the Y-axis absorbance were drawn [14].

2.3. Cell cycle analysis.

HCT-116 and MCF-7 cells were seeded at concentrations of 1 x 10^5 cells per well in a 6-well plate, and then incubated for 24 h. The cells were treated for 24 h with vehicles (0.1 percent DMSO) or 10 μM of compounds **1** or **11**. Using ice-cold, 70 percent ethanol at 4 °C, cells were harvested and fixed for 12 h. Ethanol was removed, and the cells were washed by cold PBS. Then, the cells were incubated in 0.5 mL of PBS containing 1 mg/mL Ranse for 30 min at 37 °C. In the dark, the cells were stained with propidium iodide for 30 min. Flow cytometer was then used to detect contents of DNA [63].

2.4. Annexin V-FITC/PI apoptosis assay.

For this study, annexin V-FITC / PI apoptosis detection kit was used; HCT-116 and MCF-7 cells were stained with annexin V fluorescein isothiocyanate (FITC) and propidium iodide (PI) counter-stained.1 x 10^5 HCT-116 cells were 48 h incubated with compound **1** or **11**, trypsinized, washed with phosphate-buffered saline (PBS), stained in the dark at 37 °C for 15 minutes. Finally, the cells analyzed with a cytometer of FACS Caliber flow [64].

2.5. Impact of compounds 1 and 11 on the level of anti-apoptotic markers (Bcl-2, and Bcl-xL).

Gel electrophoresis and immuno-blot analysis of proteins (**Western Blot**) method was used to examine the effect of compounds **1** and **11** on the level of anti-apoptotic markers (Bcl-2, and Bcl-xL) [63, 64]. The experiment was terminated by lysing the cells in cold lysis buffer [100mM NaCl, 10mM Tris, 25mM ethylenediaminetetraaceticacid (EDTA), 25mM Ethylene glycol bis(2-aminoethyl)tetraacetic acid (EGTA), 1% (v/v) Triton X-100, 1% (v/v) NP-40 (pH 7.4), with 1:300 protease inhibitor cocktail (Sigma) and Phosphatase inhibitor cocktail Tablet (Roche)]. The cells were then immediately frozen at −20 °C for 1 h for further lysis and collected by cell scraper and sonicated 2×10s. Total protein concentrations were determined colorimetrically using Bradford method before proceeding to the western blotting. Equal amounts (20 µg) of protein samples were mixed and boiled with SDS Loading buffer for 10 min, allowed to cool on ice and then loaded into SDS-polyacrylamide gel and separated by Cleaver electrophoresis unit (Cleaver, UK), transferred onto polyvinylidene fluoride (PVDF) membranes (BioRad) for 30 min using a Semi-dry Electroblotter (Biorad, USA) at 2.5 A and 25 V for 30 min. The membrane was blocked with 5% nonfat dry milk in TBS-T for two hours at 37ºC, in order to reduce non-specific protein interactions between the membrane and the antibody. The membrane was incubated overnight at 4°C with each primary antibody at indicated dilution. The primary antibodies against Bcl-2 (1:1500, Cell Signaling Technology), Bcl-xL (1:1000, Cell Signaling Technology), and β-actin (1:5000, Sigma) were used. The blots were then washed for three times (10 min each) with TBS-T. The membrane was then incubated with the corresponding horse radish peroxidase- linked secondary antibodies (Dako) for another hour at room temperature, followed by washing for three times (10 min each) with TBS-T. The chemiluminescent Western ECL substrate (Perkin Elmer, Waltham, MA) was applied to the blot according to the manufacturer's recommendation. Briefly, the membranes were incubated for 1 min with a mixture of equal volumes from ECL solution A and ECL solution B. The chemiluminescent signals were captured using a CCD camera-based imager (Chemi Doc imager, Biorad, USA), and the band intensities were then measured with β-actin as an internal reference protein.

3. Molecular docking

Molecular Docking Simulation studies were performed using Molecular Operating Environment (MOE®) version 2015.10. The crystal structure of Vascular Endothelial Growth Factor Receptor (VEGFR) co-crystallized with Sorafenib (PDB ID: 4ASD) was retrieved from the RCSB Protein Data Bank [74] and was used as a receptor for the docking study and Sorafenib was used as a reference drug.

3.1. Target compounds optimization.

Using the MOE program builder interface, the tested compounds **1-15**, were created into a 3D model. The target structures were checked by 2D depiction and formal charges on atoms, and then a conformational search was conducted for the target compounds. All conformers were subjected to energy minimization done with MOE until an RMSD gradient of 0.01 Kcal / mole and an RMS distance of 0.1 Å with MMFF94X was automatically measured as a force-field and the partial charges. The database of target compounds was then saved as MDB file for use in the calculations for molecular docking.

3.2. Optimization of VEGFR active site.

The VEGFR has been prepared for docking experiments by adding hydrogen atoms and their standard geometry. The atom's connections and types were checked with automatic correction for any errors that existed. Selection of the receptor and its potential atoms has been fixed.MOE Alpha Site Finder used all default items to search for the active site in the receptor structure, and then dummy atoms were created from the alpha spheres obtained.

3.3. Docking of the target compounds to the VEGFR active sites.

Docking of the tested compounds' conformational database was performed using MOE-Dock software. To ensure a reasonable docking accuracy and to determine the effect of the water molecules, the co-crystallized ligand in the VEGFR (PDB ID: 4ASD) was docked to its corresponding protein (in the absence and in the presence of water) and the RMSD values were determined between the co-crystallized ligand and docked pose. The success rates obtained were highly excellent where the active site of the VEGFR was calculated from the binding of co-crystallized ligand and saved as MOE file. The active site file of the VEGFR was then loaded, and the docking tool was used. The program specifications have been adjusted to the dummy atoms as docking site, triangle matcher as placement methodology, london dG as Scoring methodology have been adjusted to its default values. The MDB file of the ligands to be docked (Sorafenib and target compounds) was loaded, and calculations for docking were run automatically. The poses obtained were studied and the poses which had the best ligand-receptor interactions were selected and stored for calculating energy.
